# Scientific Standards and the Regulation of Genetically Modified Insects

**DOI:** 10.1371/journal.pntd.0001502

**Published:** 2012-01-31

**Authors:** R. Guy Reeves, Jai A. Denton, Fiammetta Santucci, Jarosław Bryk, Floyd A. Reed

**Affiliations:** 1 Department of Evolutionary Genetics, Max Planck Institute for Evolutionary Biology, Plön, Germany; 2 Independent Researcher, Hamburg, Germany; Liverpool School of Tropical Medicine, United Kingdom

Experimental releases of genetically modified (GM) insects are reportedly being evaluated in various countries, including Brazil, the Cayman Islands (United Kingdom), France, Guatemala, India, Malaysia, Mexico, Panama, Philippines, Singapore, Thailand, the United States of America, and Vietnam. GM mosquitoes (*Aedes aegypti*) have already been released for field trials into inhabited areas in the Cayman Islands (2009–?), Malaysia (2010–2011), and Brazil (2011–2012). Here, we assess the regulatory process in the first three countries permitting releases (Malaysia, US, and the Cayman Islands) in terms of pre-release transparency and scientific quality. We find that, despite 14 US government–funded field trials over the last 9 years (on a moth pest of cotton), there has been no scientific publication of experimental data, and in only two instances have permit applications been published. The world's first environmental impact statement (EIS) on GM insects, produced by US authorities in 2008, is found to be scientifically deficient on the basis that (1) most consideration of environmental risk is too generic to be scientifically meaningful; (2) it relies on unpublished data to establish central scientific points; and (3) of the approximately 170 scientific publications cited, the endorsement of the majority of novel transgenic approaches is based on just two laboratory studies in only one of the four species covered by the document. We find that it is not possible to determine from documents publically available prior to the start of releases if obvious hazards of the particular GM mosquitoes released in Malaysia, the Cayman Islands, and Brazil received expert examination. Simple regulatory measures are proposed that would build public confidence and stimulate the independent experimental studies that environmental risk assessments require. Finally, a checklist is provided to assist the general public, journalists, and lawmakers in determining, from documents issued by regulators prior to the start of releases, whether permit approval is likely to have a scientifically high quality basis.

Over the last 2 years there has been a dramatic increase in activities relating to the experimental release of GM insects into the environment. It is reported that commercially generated male GM mosquitoes were experimentally released into populated areas in the Cayman Islands starting in November 2009 [1], [2]. A small-scale release of the same GM mosquitoes was granted approval to take place from December 2010 to January 2011 at two inhabited sites in Malaysia (Pahang and Melaka [3]); however, it was retrospectively announced that only a single release had taken place at an uninhabited location (Bentong, Pahang). A large-scale release of the same mosquitoes is reported to have started in the Brazilian city of Juazeiro in February 2011 and will continue into 2012, resulting in the release of a total of more than 3 million mosquitoes ([4]; February 2011 was also the same month this manuscript was originally submitted for peer review). Other countries that are reported to be evaluating the release of GM insects include France [5], [6], Guatemala [7], India [8], Mexico [2], Panama [9], Philippines [10], Singapore [9], Thailand [5], the US [11], and Vietnam [5]. In September 2010, the European Food Safety Authority (EFSA) published a document entitled *Defining Environmental Risk Assessment Criteria for Genetically Modified (GM) Insects to Be Placed on the EU Market*, further illustrating the global extent of interest in releasing GM insects [12].

The first generation of GM insect technologies discussed here has been developed for use in sterile insect technique (SIT) programs designed to suppress insect populations that spread human disease or are agricultural pests (see Supporting File S1 for an extended glossary to assist non-specialist readers with most abbreviated terms used in this manuscript). SIT is a pest eradication and suppression technique employed widely across the world [13]–[15], and for the last 60 years it has been based on radiation sterilization of males (see Supporting File S1 for a brief explanation of SIT). SIT programs generally involve releasing large numbers of sterile males (which are generally innocuous) of the same pest species at a high enough frequency that the probability of wild females mating with wild fertile males is greatly lowered. If the frequency of matings with sterile males is sufficiently high over successive generations, a dramatic reduction in the pest population size can result. SIT is species specific and does not involve the dispersion of any chemicals or the release of any novel species into the environment. Transgenic GM approaches to SIT may potentially increase the efficiency and flexibility of SIT control programs compared to radiation-based SIT [16], although this awaits an empirical demonstration. While both radiation and GM-based SIT can be argued to be environmentally friendly ways to control insect pests (including insect disease vectors [17]), it is also recognized that GM insect technologies can “potentially provoke serious public mistrust and resistance to their implementation” [18]. Furthermore, it has been noted that “[GM insects] are not likely to be viewed in isolation, but as a part of a wider debate over developments in biotechnology (i.e. GM plants, stem cell research, animal cloning, etc.) and the perception of these, fuelling scepticism and/or antagonism” [19]. Consequently, companies focused on commercial GM insects and independent scientists who think that GM insect applications warrant evaluation have repeatedly identified the need for high quality multi-disciplinary scientific research in this area. This is in addition to the need for transparency and public involvement in the regulatory approval process for any environmental releases [18]–[25]. When considering GM insects, it is important to appreciate that genetic modification/engineering is a highly flexible technology capable of generating an almost unlimited variety of genetic changes, creating organisms with a broad range of novel properties. Consequently, discussion is generally only informative if it is clearly defined what type of genetic modification is being considered, rather than attempting to regard multiple GM approaches as a single unified entity. The properties of transgenic constructs that have so far been considered in regulatory documents can usefully be separated into two classes:

1. **Fluorescent markers.** Genetic modifications that express only transgenic fluorescent proteins. Using a microscope with epi-fluorescence optics [26], it is possible to distinguish fluorescent individuals released from rearing facilities (or their progeny) from wild non-florescent individuals. Fluorescent markers are of particular value in monitoring the size of the wild pest population during SIT releases. An additional elaboration is the monitoring of the mating success of sterile released males through the presence of fluorescent sperm in wild females [27].
2. **Repressible dominant lethal (RDL).** Genetic modifications that are designed to conditionally kill offspring inheriting them. Such constructs can be engineered to have very distinct properties: (a) *sterilising constructs;* engineered to kill all the offspring of individuals released into the environment and intended to replace the use of radiation for sterilization [28]–[32]; (b) *sexing constructs;* engineered to kill females prior to releases in SIT programs, where only males are required for release [33]–[36]; and (c) *female-killing constructs;* engineered for use in SIT programs, to kill all daughters of individuals released into the environment but not their sons [34], [35]. Theoretically, the use of female-killing constructs can be more efficient than the use of fully sterile constructs where lethality is not sex selective [16].

While properties of the particular constructs so far considered in regulatory documents can be reduced to the above two classes, many can also be combined to generate a desired set of joint properties (e.g., fluorescently marked, female-killing constructs). It is important to note that all the transgenic constructs discussed here in the context of existing field trials are not intended to become stably established in the environment (i.e., in the absence of continued release of GM insects, the introduced transgenes are likely to ultimately be lost from a wild fertile population). Consequently, they are considered to be a type of genetic modification with the lowest environmental concern (Table 2 on page 19 and section 5.3.4.2 in [21]). While currently premature, it is in the authors' opinion quite conceivable that given sufficient experimental evidence (see discussion), constructs within these classes could at some point in the future justifiably be subject to minimal regulation.

Given the recent increase in regulatory activities involving GM insects across the world, we have chosen to examine the pre-release regulatory processes in the first three countries known to have authorized free releases of GM insects into the environment: the US, the Cayman Islands, and Malaysia. This is done from the perspective of (1) the transparency of the regulatory process; and (2) identifying the scope of the scientific evidence on which regulatory decisions were made. There is a particular focus on the US regulatory experience as the US has by far the longest record of regulatory decisions on GM insects and it is currently being promoted as a global regulatory model [22], [37]. This article does not seek to directly evaluate the outcome of the regulatory decisions made, but is intended to provide a generally understandable analysis of how scientifically relevant aspects of the decision-making process were conducted.

## A Short History of US GM Insect Regulation 2001–2010

The US has approved 14 field trials since 2001 with pink bollworm (*Pectinophora gossypiella*), a moth that is an agricultural pest of the cotton plant. The federal US authority responsible for the regulation of GM insects that are potential plant pests is the Animal and Plant Health Inspection Service (APHIS), an agency of the United States Department of Agriculture (USDA). The APHIS unit responsible for granting field trial permits is Biotechnology Regulatory Services (BRS-APHIS), which acts in consultation with state regulators. Permit applications for field trials of GM insects are submitted to BRS-APHIS and contain a comprehensive description of the proposed experiment(s) [38]. Applications have only been made publically available for two of the 14 granted permits (and none of the five withdrawn ones or the one pending application [39]). With the exception of the two published permits, all available application information is limited to the notification details published on the APHIS website, a 2010 example of which is copied below [39].

*10-074-102r* [application ID]

*United States Department of Agriculture/Animal and Plant Health Inspection Service* [Name of permit applicant]

*Reg article: Pink bollworm* [species]

*MG-Dsred Fluorescent Protein Expression* [description of genetic modification]

*Releases in: AZ* [state]

*Release* [type of permit: release or inter-state movement]

*Received: 15-MAR-2010*

*Status: Issued 13-APR-2010*

Both of the publically available permits were published as parts of Environmental Assessment documents (EA, a concise environmental impact assessment document in the US regulatory system). The first EA was published in 2001 (2001-EA, [40]) for a caged field trial with fluorescently marked pink bollworms, and a second one for a free release field trial of the same stock in 2005 (2005-EA, [41]). All permits granted by BRS-APHIS have been to other research units within the same US government agency, APHIS. To date no granted permits have been directly applied for by commercial companies, though it is clear that some permits are being applied for by US government agencies to provide information on commercial stocks (see below).

In 2008, APHIS published an EIS entitled *Use of Genetically Engineered Fruit Fly and Pink Bollworm in APHIS Plant Pest Control Programs* (2008-EIS, [42]). The document considers the potential integration of GM insects into ongoing SIT programs run by APHIS in the following four agricultural pest species: pink bollworm moth (*P. gossypiella*), Mediterranean fruit fly (*Ceratitis capitata*), Mexican fruit fly (*Anastrepha ludens*), and oriental fruit fly (*Bactrocera dorsalis*). The 2008-EIS formed the basis for an announcement in May 2009 that APHIS will “permit integration of genetically engineered insects into its plant pest control and eradication programs” ([11]; see Supporting File S2 for a more extended summary of US regulatory history). EIS documents are described as the most comprehensive documents produced in the US regulatory system; however, they are not necessarily scientifically more rigorous than the alternative EA documents, which are considered “concise” [43], [44]. This is because EIS documents can have a very broad scope, as they are principally intended to evaluate the impact of proposed agency policy changes on a broad “programmatic” basis at a national level. EA documents instead are generally focused on specific actions in a single species at named locations (contrast §372.5 sections a and b [43]).

## The 2008-EIS as a Regulatory Document of Global Scientific Value

The 2008-EIS is currently actively promoted as a document to establish global precedents [22], [37] and was recently described by one of its principal authors (an independent biotechnology regulatory consultant/agent) in the following terms:

*Use of genetically engineered fruit fly and pink bollworm in APHIS plant pest control programs* is the title of the world's first environmental impact statement (EIS) on any kind of transgenic organism, either plant or animal, prokaryote or eukaryote. This programmatic EIS is also a major part of the world's first official government regulatory process on any transgenic insect […]

This EIS is of major value for genetic markers and *Aedes*, possibly *Anopheles*, sterile insect technique (SIT) population suppression using repressible lethal genetic constructs instead of radiation to sterilize the insects. This EIS also has some applicability for population replacement strategies for *Aedes* spp. or *Anopheles* spp. using gene introgression/driver mechanism strategies. (Page 42 [22])

The value of the 2008-EIS to environmental risk assessments of tropical mosquitoes andor insect population replacement strategies is difficult to discern, as neither is considered in the 2008-EIS main text (mosquitoes are briefly mentioned in appendices C and D; population replacement is mentioned once in appendixes E and K [42]). Population replacement strategies are intended to stably establish transgenes in wild populations and represent a higher class of environmental concern (see Table 2 on page 19 and section 5.3.4.2 in [21]).

To examine the scientific quality of the analysis of environmental impact in the 2008-EIS, we seek here to establish the scope of the scientific evidence cited for the five example applications given in its executive summary (page vi in [42]):

1. Use of fluorescent marker constructs in radiation sterilized fruit fly species.
2. Use of sterilizing RDL constructs in fruit fly species.
3. Use of female-killing RDL constructs in fruit fly species.
4. Use of fluorescent marker constructs in radiation sterilized pink bollworm.
5. Use of sterilizing RDL constructs in pink bollworm.

## What Constitutes a “Substantial Body of Scientific Evidence” in the 2008-EIS?

The first mention of GM insects in a published US regulatory document that we can find is in a 2001 document entitled *Fruit Fly Cooperative Control Program, Final Environmental Impact Statement—2001* [45], which states that:

Biotechnological control methods are currently under development and are not available for program use at this time. Because the circumstances surrounding their uses are uncertain, information on their potential effects upon land, water, or air resources and quality cannot be determined at this time. (Page 82)

In general, detailed information relative to the environmental impacts of those other forms of biotechnological control are unavailable. No substantial body of scientific evidence relative to evaluating the impacts of this control method exists, nor can it be summarized within this document. (Page 31)

The value of this document, which dealt with fruit fly suppression techniques, is to establish a time period when the integration of biotechnological techniques in fruit fly programs was, in the opinion of APHIS, not tenable based on the then available evidence. It also implies that a “substantial body of scientific evidence” encompassing information on “land, water, or air resources” would be necessary to revise this opinion. In the intervening period between the drafting of the above document ([45] started in 1999) and the 2008-EIS ([42] started in 2006), there was a diametric change in the conclusions reached with respect to the impact of biotechnological/GM pest management. If this change was not based on an increased tolerance to risk, examination of the references cited in the 2008-EIS (there is no data presented in the document) should allow identification of the new body of evidence that was considered sufficient to reverse the previous position; there are approximately 170 scientific publications cited in the 2008-EIS (not including government reports, etc.). Supporting File S3 shows all the published written studies cited in the 2008-EIS that could have been in any way reasonably related to each of the five example applications (regardless of whether or not they were in fact cited in this context). From this table it can be determined that there was a substantial, relevant, and diverse body of scientific evidence with which to assess the likely environmental impact of the use of fluorescent markers in insects (example uses 1 and 4). This includes laboratory studies in all four of the target species and a wide range of other insect species, although no published field studies are cited.

However, for all experimental techniques involving the release of sterile or semi-sterile individuals with RDL constructs (example uses 2, 3, and 5 in the executive summary of the 2008-EIS), discussion could only have been limited to two laboratory studies in one of the four species covered by the document [28], [35], with an additional five studies in the related fruit fly *Drosophila melanogaster* [29], [32], [33], [35], [36] and one in a mosquito [30]. No published field trial data was cited. The small number of published experimental studies cited in the 334 page 2008-EIS, upon which endorsement of these GM techniques was based (sterilizing constructs and female-killing constructs), can very easily be missed even by specialists. The principal reason for this lack of clarity is the consideration within a single document of a range of genetic techniques in four different species, with a large number of potential applications across an entire nation (and potentially internationally, page 21 [42]).

In this light, it is instructive to consider how EIS documents could have looked in 2008 if separate EISs had been drafted for each of the RDL constructs. This would have permitted a much more comprehensible consideration of the extensive experimental literature relating to the use of fluorescent markers in the four species covered by the 2008-EIS (Mediterranean fruit fly, Mexican fruit fly, oriental fruit fly, and pink bollworm; see Supporting File S3). At the same time, this would have permitted clearer identification of which combination of the seven cited laboratory-based publications ([28]–[30], [32], [33], [35], [36], investigating at least four distinct transgenic constructs) were considered a significant body of environmental evidence for each of the transgenic approaches mentioned in the 2008-EIS executive summary. Furthermore, of the seven cited publications, none included any information on RDL constructs in the Mexican fruit fly, oriental fruit fly, or pink bollworm (two described RDL constructs in the Mediterranean fruit fly [28], [35]).

## The Use of Unpublished Evidence in Regulatory Documents around the World

The 2008-EIS is presented as a scientific document of global value ([37] and page 42 in [22]). Generally, scientific documents require that statements made within them should conform to accepted norms of scientific publications. Fundamentally, this requires that statements made within it should be established either through the presentation of primary data or unambiguous citations of relevant written publications. Where unpublished citations are used, it should not be to establish points of central importance, and in all instances where unpublished data is used, it should be stated why it is justified (e.g., [46]). The selective use of unpublished or non-peer reviewed evidence to support contentious conclusions has been repeatedly questioned (e.g., [47]). An example of unpublished evidence referred to in the 2008-EIS is a field trial conducted in 2007 that involved the release of >20 million fluorescently marked pink bollworm moths onto 100 acres of isolated cotton plants in Arizona. Despite being probably the most referred to experiment in the 2008-EIS (e.g., pages 40, 41, 55–57, and D-16 [42]), all data generated during this publically funded study remain unpublished. The inclusion in the 2008-EIS of some selected results and conclusions cannot substitute for publication of the data and a detailed description of the experimental procedures in a manner that would enable independent critical evaluation. Notably, US federal regulations for the drafting of EIS documents state:

No material may be incorporated by reference unless it is reasonably available for inspection by potentially interested persons within the time allowed for comment. Material based on proprietary data which is itself not available for review and comment shall not be incorporated by reference. (§1502.21 [48])

Of the 14 granted permits, all to APHIS units, no written experimental descriptions and data have been published in 9 years of field trials (though see [49]–[51], which provide selected summaries of some results, but with insufficient experimental descriptions and data to permit critical evaluation). A peer-reviewed publication by APHIS scientists is reportedly in preparation [52]; however, after such a long delay, even this welcome step could not fully address the failure to release experimental data in a timely manner (e.g., before it is cited in regulatory documents or when it is requested under the Freedom of Information Act, see below). If, for example, experimental results had been submitted as part of a permit application by a commercial or independent third party, there is a well-established precedent that such data cannot be released by government agencies without consent [53]. However, these experiments may reasonably be viewed as a form of publicly funded government testing, which commonly involves routine publication of data, e.g., the US National Toxicology Program [54]. Additionally, the failure of US regulators to publish data (in this case their own data) prior to their inclusion in regulatory documents represents a worrying precedent for the scientific quality and transparency of future environmental impact assessments.

The failure to cite published experiments is also a feature of various documents issued by Malaysian regulatory authorities relating to the release of *A. aegypti* mosquitoes in 2010–2011. This includes the failure of pre-release documents to cite published data for semi-field trials (point 2 [3]) and predation toxicity experiments, both of which are referred to in released documents (point 5 [3]). The Cayman Island regulatory authorities did not, as far as we can determine, publish any regulatory documents prior to the release of transgenic mosquitoes in 2009. However, subsequent to the scientific presentations made about the releases on 3–7 November 2010 [55], [56], limited details of the release have become available [9], [57] and have become the focus of local (see reader comments to [58], [59]) and international controversy [2], [9].

## Do Restrictions to Public Scrutiny of Regulatory Documents Facilitate Practices that Undermine Public Confidence?

As detailed above, only two of the 14 permit applications granted to APHIS research units have been published. It was apparently the original intention of the APHIS regulatory unit to immediately publish all received permit applications, based on a 2001 document issued by APHIS as a part of its “expanding efforts of APHIS to communicate with interested entities and to make the permitting process as transparent and understandable as possible” (the original document is no longer available on the APHIS website, point 2c [60]). However, it appears that at some point this practice was abandoned.

In an effort to obtain experimental details we wrote to BRS-APHIS asking for information on a specific permit granted in 2005 (permit 05-118-01r), but were informed in writing that information was “only available through a FOIA request” (a request under a Freedom of Information Act). One of the authors (FAR, a US citizen) filed an FOIA request with APHIS in July 2008, which took 1 year and 11 months to be processed. It resulted in a copy of the permit (which was not previously publicly available) and correspondence up to and including the granting of the permit (Supporting File S4), but crucially, no experimental data was forthcoming. This is despite the regulator (BRS-APHIS) stating it contained no commercially sensitive information (28/6/05 letter from BRS-APHIS to the Arizona Department of Agriculture, page 2 Supporting File S4).

At least a partial explanation for the lack of publically available information lies with the use of an administrative procedure called a “categorical exclusion” (CE), which allows US regulators to rely on earlier similar EAs rather than draft a new one. CEs are the most frequently employed method of permit approval used by US federal agencies. CEs speed up the application process by removing the need to regenerate largely redundant EA documents. As the time taken by APHIS to grant FOIA requests greatly exceeds the 2 months it takes to approve most field trial permits by applying CEs, the use of CEs effectively removes all statutory requirements to make permits publically available (despite the fact that permits are explicitly subject to FOIA requests, page 8 [38]).

In a letter from the federal regulator (BRS-APHIS) to the Arizona State Department of Agriculture (obtained thorough the FOIA request, page 2 Supporting File S4), it is argued that the use of a CE is appropriate for the research proposed in newly obtained 2005 permit application because it was “similar and equivalently contained” to experiments assessed in the earlier published 2001-EA [40]. It was argued that it was unnecessary to generate a new EA as this is only necessary “When a confined field release of genetically engineered organisms or products involves new species or organisms or novel modifications that raise new issues” (§372.5 d4 [43]). While it is reasonable to argue that the degree of physical containment described in the 2001-EA (pages 6–10 [40], which is made available as Supporting File S5) and in the newly obtained 2005 permit (pages 8–19 Supporting File S4) were similar, as both used cages of the same design and the same mode of moth transportation to the experimental site, the degree of biological containment differs.

The 2001-EA permit was granted for work on a particular pink bollworm stock with a simple fluorescent construct where all released individuals had been sterilized prior to the start of the experiment using a dose of radiation established to be sufficient to cause 99.9% sterility; in addition, the wings of all females had been manually removed [40]. The description in the newly obtained 2005 permit detailed experiments involving individuals with transgenic RDL constructs that had not been radiation sterilized and where all individuals were fully winged. Most of the proposed study is on two pink bollworm stocks that possess a RDL construct based on the RIDL system (Release of Insects carrying a Dominant Lethal, a group of diverse constructs developed by Oxitec Ltd., one configuration of which is discussed here) in addition to a fluorescent marker. The stocks are described in the application as having sterility “as high as 100% with a range of 60%–100%”, though the applicants speculate that sterility could be much higher under field conditions (page 17 Supporting File S4). Despite the fact that the degree of biological containment built into the 2005 experiment was potentially dramatically reduced compared to the earlier 2001-EA experiment by up to a 40% increase in fertility and females being capable of flight, this did not, in the opinion of BRS-APHIS or Arizona state officials, raise any significant new issues.

This poses the question of whether US regulators consider all transgenic constructs in a given species equivalent. For example, in the case of pink bollworm, has the publication of the 2001-EA (assessing caged experiments) and the 2005-EA (assessing free release experiments), both involving transgenic constructs of lowest environmental concern, ensured that no information on any subsequent applications involving more complex transgenic constructs is disclosed? The Malaysian national safety board have made a prodigious effort to engage the public prior to regulatory approval [61] and subsequent to approval [3], [62]–[66]. However, it is noteworthy that the permit application (made by the Malaysian Institute of Medical Research) was not made publically available, and this is also the case for the Cayman Islands trial, where even the identity of the permit applicant is unclear [57].

## Established Precedents in US Regulation of GM Insects and Their Global Impact on Building Confidence in GM Insect Regulation

At the US federal level, the National Environmental Policy Act (NEPA) regulates protection of the US environment. This act includes numerous statutory and voluntary provisions for publishing information and facilitating citizen engagement in environmental decision-making processes (outlined in *The Citizen's Guide to NEPA: Having Your Voice Heard* [44]). However, despite these advantages, the flow of information to the public and scientific domain over the last 9 years has been limited. In fact, it can be argued that dissemination of relevant information is so restricted that it undermines the value of public consultation exercises, as comments will almost by definition be ill-informed and readily dismissed as such.

If APHIS hopes to realize the reduced costs and increased effectiveness that GM insects may offer to its pest control programs, without engendering public mistrust, it would appear prudent to reverse some of the precedents that have become established in some of its units. In this light, the regulation of GM insects in the US would be greatly strengthened by APHIS making the following procedural changes (if necessary, being restricted to experiments conducted at federal research facilities):

1. Permit applications should be published immediately upon determining that the application is substantially complete (as was the policy in 2001 [60]), without the deletion of scientifically important information (e.g., page 9 [38]). This change should be made retrospectively, with all previously granted permit applications being published immediately.
2. Complete experimental data from field trials substantially conducted at federally funded research facilities should be routinely placed into the public domain and this should be done retrospectively for field trials that have already been completed (the USDA website http://www.usda.gov/open, which already hosts 148 datasets unconnected to GM insects, would appear to be well suited for depositing raw data).

Both actions would be very much in line with the advice of the USDA reform advisory committee AC21 (Advisory Committee on Biotechnology and 21st Century Agriculture, points 6 and 12 [67]). Finally, given the deficiencies of the 2008-EIS detailed above, it would appear advisable that this document should not play any role in justifying the future use of CEs or permit approvals in any jurisdiction.

The fact that government agencies—APHIS in the US and the Malaysian Institute of Medical Research—are visibly taking the lead in experimentally evaluating a very new technology, which has already attracted some controversy, is a very positive development. This should make it much easier to ensure the free flow of impartial information into the public and scientific domain. Furthermore, APHIS' role, as not only the US regulator, but its global position as the largest generator of field data on GM insects and the biggest potential contractor of commercial GM insect services, places it in an unparalleled position to establish positive regulatory precedents for this developing technology. For example, if APHIS were to announce that it would prioritize the evaluation of GM insect technologies where proprietary considerations did not impede free exchange of scientific information (see Supporting File S2), this would build domestic confidence in this technology and globally stimulate the independent and interdisciplinary experimental studies that high quality environmental impact assessments (EIAs) require in order to minimize the risk of unacceptable harm to the environment. Decisive steps by APHIS policymakers could provide a boost to the efforts of APHIS to transparently demonstrate the scientific quality of its decision-making processes and would also act as a powerful positive global role model in this respect.

## When Considering the Potential Environmental Impacts of Complex Biotechnological Approaches, How Useful Are Highly Generic Discussions?

Given our assertion that the discussion of environmental impact in the available regulatory documents is, in at least some instances, insufficiently case specific to be meaningful, it seems reasonable to attempt to elucidate what level of specificity is necessary for meaningful EIAs of GM insects.

The discussion of well-understood and very simple transgenic constructs (e.g., those expressing only a fluorescent marker) could arguably be adequately considered through generic discussions. However, this is almost certainly not the case for the vast majority of other types of transgenic constructs, with diverse and potentially complex properties when released into wild populations. Here, we give one relevant example to illustrate how outwardly small differences in the technical engineering of transgenic constructs, the species they are placed in, and the application they are used for can result in important differences in the environmental hazards that should reasonably be expected to receive expert consideration.

### Meaningful Consideration Should Be Specific to a Transgenic Construct in a Specific Species

The RIDL (Oxitec Ltd.) configuration described in [28], [30] and the Horn & Wimmer system ([29], [31] Georg-August-Universität Göttingen) are different types of RDL constructs cited in the 2008-EIS [42]. Both systems rely on expression of a synthetic protein called tTA (tetracycline-controlled transactivator [68]). In the Horn & Wimmer system, low levels of tTA expression are restricted to the first few hours of embryonic development in the progeny of released males, which then die as embryos. In the RIDL system, tTA is highly expressed both throughout the body of the released males and in their progeny until death at late larval stage [28], [30]. If ingestion of tTA by insectivores in the environment warrants expert risk assessments (the merits of doing this are debated in the 2008-EIS, pages C-21, E-4 to E5, and E-24, and E-Handler letter [42]), this concern clearly varies with the type of system used. For instance, in the Horn & Wimmer system only predation of very early embryos needs to be considered, while in the RIDL system [28], [30] predation at all life stages would need to be considered (embryonic, larval, and adult). Furthermore, it is important for risk assessments to be specific to a species, so that the role it plays as a food source for insectivores at various life stages can be accurately assessed. This illustrates how analysis of the environmental risks of one transgene may not be applicable to a different transgene even if there are substantial underlying structural similarities in the way they were engineered. Finally, in many instances it may also be necessary to discuss the impact of specified transgene insertion sites, as the same transgenic construct inserted at different locations in the genome can significantly vary the expressivity or penetrance of phenotypic traits that could impact risk assessments (e.g., the degree of sterility [31] or effectiveness of female killing [35]).

### Meaningful Consideration Should Be Specific to an Application

SIT can be used in a wide range of situations, the extremes of which can be represented at one end of the spectrum as preventative release programs (PRPs) and the long-term suppression of large populations at the other. PRP releases occur into areas where there are few or even no fertile females and are intended to prevent the establishment of pest populations in areas at risk of infestation. The probability of selecting for resistance genes present in the wild population (which could allow the unintended transfer of transgenes to the fertile wild population) is proportional to the number of transgenic fertilized eggs generated. In PRP releases there may be few if any of such eggs; however, in long-term suppression programs there may be billions of such eggs. Consequently, it can be seen that the type of application has a different probability of selecting for RDL resistance.

This question of selection for RDL resistance was specifically raised during the public comment period of the 2008-EIS (Jorge Hendrichs FAO/IAEA, page E-24, and also by Alfred Handler USDA-ARS, E-Handler letter page 269–270 [42]) and in prior literature [13]. This concern is far from being a speculative hypothesis; insensitive mutations to the VP16 domain that forms half of the tTA protein have been repeatedly generated in yeast (though they are reported as recessive [69], [70]). In response to this concern, a rebuttal is given on pages E-9 to E-13 of the 2008-EIS that gives the unsubstantiated impression that the probability of RDL resistance evolving is equivalently low for all types of SIT applications. However, the fact that resistance has arisen to some extent to almost all widely used insecticides provides a striking illustration of the practical difference between events that cannot occur (i.e., zero probability) from those that can occur but with low probabilities. Despite the establishment of standard laboratory protocols to rapidly estimate the likelihood of resistance arising to novel insect suppression techniques [71]–[74], no such experimental study has been published for RDL techniques.

## Generic Considerations of GM Insects Are Often of Limited Scientific Value

The value of EIAs largely composed of generic or incomplete discussions, which are neither technique nor species specific, are as a consequence of very limited value and prone to generating conclusions that can be misleading. The degree of specificity necessary for meaningful EIAs is explicitly addressed in the 2007 North American Plant Protection Organization (NAPPO) guidelines on confined field releases of transgenic arthropods [75] and the 2006 Food and Agriculture Organization and International Atomic Energy Agency/(FAO/IAEA) report entitled *Status and Risk Assessment of the Use of Transgenic Arthropods in Plant Protection* (section 5.3.4.2, pages 22–24 [21]). Both of these documents are cited in the 2008-EIS [42]. The FAO/IAEA report has a non-exhaustive list of approximately 60 questions focused on hazard identification, and this list is reproduced in full in the 2008-EIS (page D-5 to D-8 [42]). The first two questions are addressed in the 2008-EIS in the format of a question followed by a clear discussion of relevant information. However, this approach is subsequently abandoned, resulting in a large proportion of relevant questions from the FAO/IAEA [21], EFSA [12], and NAPPO (which is legally binding [75]) documents not being recognizably addressed anywhere in the 2008-EIS (though it should be noted that the EFSA document did not exist at the time of the drafting of the 2008-EIS). Finally, while it is generally accepted that biotechnology regulation should be on a “case-by-case basis”, this clearly requires that regulatory documents be specific enough to a case to be scientifically meaningful.

## The Release of GM Mosquitoes in the Cayman Islands and Malaysia (2009–2011)

**Figure 1:**
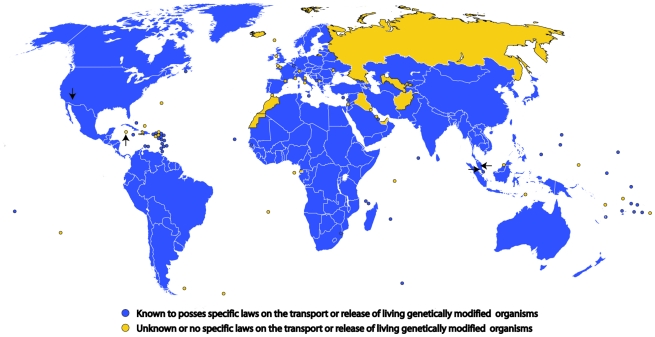
Global extent of legislation relating to living genetically modified organisms by 2010. One hundred and sixty governments are parties to the Cartagena Protocol on Biosafety, and ten that are not parties have chosen to submit documents to the Biosafety Clearing-House (an instrument set up under the Cartagena Protocol) relating to “National Laws, Regulations and Guidelines” governing the release or transportation of living GM organisms. Of the remaining countries, the US, Israel, and Singapore are known to have specific laws regulating living GM organisms (all the above countries and categories are colored in blue). Consequently, countries and territories colored blue have at least some specific laws governing the release or transportation of living GM organisms. For the remaining 21 countries that are not parties to the Cartagena Protocol on Biosafety and some overseas or disputed territories, it is unclear if they have any relevant laws (colored yellow). The locations of field trials mentioned in the text are indicated by arrows. Malaysia and the US both have comprehensive, specific legislation, and Malaysia is also a party to the Cartagena Protocol on Biosafety. While the UK is a party to the Cartagena Protocol on Biosafety, the Cayman Islands (which are a UK overseas territory) has not become a party to the protocol despite encouragement to do so by the UK government [78], [112]. A quote from a senior researcher of the research institute conducting the field trial in 2009 [9] confirms that the Cayman Islands had no enacted legislation relating to living GM organisms (only draft legislation is mentioned [113]). See Supporting File S6 for additional map details.

The *A. aegypti* mosquito is the principal vector of dengue fever, a human disease that is of pressing concern in many countries around the world [76]. The field trials discussed below are focused on developing a novel approach to control this disease, through the release of genetically sterilized *A. aegypti* males of a stock called OX513A (which contains a configuration of the RIDL system developed by Oxitec Ltd., UK). The field trial(s) on the Caribbean Cayman Islands (an overseas territory of the UK), which appear to have commenced in 2009 and have been reported to be ongoing (in October 2010, point 7 [63]), involved the large-scale release of OX513A mosquitoes (which carry the configuration of the RIDL system discussed above [28], [30]). In 2010, we searched the web pages of the Cayman Mosquito Research and Control Unit (http:/www.gov.ky/mrcu), which reportedly conducted the trial, and other pages of the government of the Cayman Islands, but were unable to find any regulatory documents on the trial. However, on 13 January 2011, over a year after releases commenced [1], a document was uploaded to the UK Parliament website entitled “Risk Analysis – OX513A *Aedes aegypti* Mosquito for Potential Release on the Cayman Islands (Grand Cayman)” [57]. This deposition (apparently by a UK government department) occurred in response to written parliamentary questions by Countess Mar of the Lords Chamber [1], [77]. Unfortunately, key details about the document are omitted, including the identity of its authors, and as a result the origin and purpose of the document is unclear. This is perhaps unsurprising, as the Cayman Islands were at the time of the releases (and remain) one of the few areas in the world without any enacted specific legislation regulating the release of living GM organisms into the environment (see Figure 1 and [78]). From answers to UK parliamentary questions it can be deduced that the document was part of the notification to the UK government from Oxitec Ltd. that they had started exporting mosquito eggs on 4 November 2009 [1] that were intended for release in the Cayman Islands. This notification, required under European Community regulation 1946/2003, was received by the UK government on 1 December 2010 [77], [79]. The document is dated October 2009, which is the same month as the earliest public pre-release written notification of the releases we can find [80]. Based on the date and the content of the document, it appears that it formed at least part of the basis for the regulatory approval of the very first intentional free release of GM mosquitoes in the world. Consequently, it is interesting to examine how it conforms to scientific expectations for an EIA; it is, however, important to note that the document is not complete (for example, it includes drafting notes [81] and does not include experimental details of the releases).

Despite a number of unambiguous biological misunderstandings, collectively these probably have few practical consequences for identifying potential hazards of OX513A mosquitoes (e.g., page 2 [57], horizontal gene transfer between eukaryotes [82], [83] and fertile hybrids between insects species [84]). However, of greater importance is the very poor referencing of the document [57], particularly of key points. For example:

The characteristics of the OX513A *Aedes aegypti* have been thoroughly evaluated by several institutions worldwide, e.g. in France, Malaysia (Lee et al, 2008) and Thailand (Khongtak et al, 2009).

Both of the above citations are from reports that are not publically available. Of the three citations supporting another questionable pivotal scientific assertion—

However, classical SIT has hitherto not been successful with mosquitoes such as Aedes in spite of much effort by the International Atomic Energy Agency (IAEA) and others because gamma radiation, used in classical SIT to sterilise the insects, renders the mosquitoes very weak and unfit to compete with the wild male mosquitoes. However, this problem seems to have been overcome because OX513A uses genetic methods instead of radiation to achieve sterility, therefore the genetically sterile insects have been reported to be fitter and competitive (Jain 2006; Lee et al. 2006; Phuc et al. 2007;).

—Jain 2006 is a newspaper article, Lee et al. 2006 is missing from the bibliography, and Phuc et al. 2007 includes no data on the relative fitness of male mosquitoes. Regardless of any potential shortcomings, this document does, however, give the first complete listing (that we are aware of) of all the donor organisms from which the OX513A construct is built.

However, the most troubling aspect of this risk assessment document is the absence of any discussion of potential environmental or health hazards that are specific to the released OX513A stock (distinct from potential hazards that are shared with a non-transgenic mosquito stock). This striking omission appears to be justified in the last section of the document, which is reproduced below.

**3. Statement on the overall risk analysis**

Risk analysis has been conducted on the hypothetical release of genetically modified *Aedes aegypti* mosquitoes expressing a self-limiting trait and a marker gene, as present in strain OX513A. The independent risk assessment was conducted by over 70 Malaysian scientists as part of a UNDP/University of Malaya sponsored workshop on the Risk Assessment of Transgenic Insects in Nov 2008. The proceedings of the workshop have been written up and are available (Beech et al, 2009).

The conclusions of the risk assessment were that a potential release of OX513A male mosquitoes would have a negligible risk to human health or the environment, although certain risks were identified as low risk from the trial:

[This is followed by the repetition of three of the four potential hazards listed in Beech et al. 2009 [85] as having been assigned “low” or “medium” risk (as opposed to all the other listed hazards which were assigned “negligible risk”/“not important to human health or the environment”). The reasons of the omission of the fourth potential hazard is unclear.]

While it should be kept in mind that the purpose of the document is unclear, the suggestion that Beech et al. (2009) [85] played any role at all in approving the world's first intentional release of a transgenic mosquito (into an inhabited town) is very surprising. Primarily, because Beech et al. (2009) is: (1) a report on a teaching exercise conducted as part of a capacity building project; and (2) a generic risk assessment. While the hypothetical teaching exercise is described as being a mass release of RIDL (a trademark applied to a number of differently configured constructs, one of which is present in OX513A) mosquitoes, it is not identified as being specific to any named stock (see discussion of the limited scientific value of generic discussions). Finally, the “independence” of the Beech et al. (2009) risk assessment, as stated in the passage reproduced above, is valid in the sense that no Cayman Islands scientists are listed as being participants of the workshop session.

The field trial approved by the Malaysian National Biosafety Board was applied for by the Malaysian Institute for Medical Research and releases occurred from December 2010 to January 2011. It involved the temporary small-scale releases of GM *A. aegypti* mosquitoes at two locations in mainland Malaysia (Pahang and Melaka). The Malaysian government has made impressive efforts to establish a clear regulatory framework (Biosafety Act 2007, which came into effect in December 2009) and is a party to the Cartagena Protocol on Biosafety. The decision to approve the trial was taken with a substantial degree of public and political consultation (e.g., question 1 [63]; [61]). A significant amount of comprehensible information was made available prior to release approval. However, the permit application is not publically available. A document entitled “risk assessment report” by the Malaysian Genetic Advisory Committee provides an abbreviated summary in its 14 pages of the process of risk assessment in permit approval [64]. The failure to reproduce the full risk assessment matrix they used to identify the potential hazards they considered (unlike the report that appears to have been used as a model [86]) weakens the scientific value of the document and undermines its ability to transparently demonstrate what plausible hazards have received adequate expert consideration (see discussion below). Significantly, the document highlights three references that were particularly valuable sources of information in their risk assessment. The first was the 2008-EIS, clearly establishing that this scientifically deficient document is being used as the basis for regulatory approvals around the world. The second was the risk assessment and results of the Cayman Islands field trial (no part of which was made publically available until after the Malaysian release had commenced), establishing that despite the unanswered ethical questions raised by this trial [2], it too is being used as the basis for regulatory approvals around the world. These significant omissions in the information made publically available prior to releases in the Cayman Islands, Malaysia, and the US leaves external scientists in the uncomfortable position of having to rely on media reports, oral presentations, meeting minutes, and patent documents as their only timely means to obtain some scientific details (e.g., [4]).

## Summaries of EIAs Can Be of Limited Scientific Value and May Not Build Public Confidence

Without the pre-release publication of complete risk assessment documents detailing all the potential hazards analyzed, it is often impossible to establish which have been considered (and by whom) and if any obvious hazards have been overlooked for rigorous consideration. It is anticipated that failure of regulatory authorities to convincingly advertise the scientific quality of their decision making, prior to releases (when public interest is high), risks eroding public confidence even when experiments proceed without incident. Below is an illustrative discussion of an obvious potential hazard specific to OX513A about which we are unable to find any written evidence in pre-release documents that it has received regulatory consideration.

The particular RIDL construct in OX513A is engineered to express the synthetic protein called tTA (NCBI accession number CAI26306.1) at very high levels in all cells at all life stages and in both sexes when released into the wild [28], [30]. If this RIDL construct is placed into blood feeding mosquito species, there is the plausible concern that females could inject tTA into humans along with mosquito salivary gland fluids that are transferred as part of a normal bite. Importantly, this concern arises even if only male mosquitoes (which never bite) are released. This is because tTA-expressing females would occur in the environment in at least three circumstances: firstly, if heritable resistance to the RIDL construct was to arise in the wild; secondly, while the mechanical removal of females prior to release is highly effective, it is not 100%; and thirdly, when RIDL stocks are only partially sterile under field conditions. In fact, OX513A males are only partially sterile, and when they mate with wild females they will produce 2.8%–4.2% the normal number of eggs, half of which will be biting daughters [30], [63], [64]. If these laboratory estimates of fertility prove representative of field conditions, then even if only males are released, it is probable that some human inhabitants will be bitten by transgenic females.

The probable presence of transgenic females in the environment requires that a more complex series of potential hazards would need to be considered in a credible EIA than would be necessary if the presence of females in the environment was highly improbable. For example, the assumption that the transgenic tTA protein is not expected to be secreted into the salivary fluid (which is injected as part of a normal bite) because it does not have a secretory signal peptide sequence is questionable based on the fact that (1) not all proteins found in the salivary fluid of *A. aegypti* have identifiable secretory signal sequences [87]; and (2) levels of expression of tTA proteins are anticipated to be extremely high in all cells ([28], [30] even in heterozygotes). Therefore, it may not be reasonable to assume that physiologically significant amounts of tTA will not be found in the salivary fluid. While it is well established that almost any substance the human body is exposed to has the potential to cause an undesirable allergic response, the probability that a given compound elicits such a response is extremely low [88]. However, the hazard to sensitive humans is sufficiently great that all GM plants intended for human consumption are assessed for allergenicity [88]. The desirability to assess the allergenicity of transgenes in GM insects is specifically mentioned in a recent European Union (EU) document that recommends using the food safety framework established for GM plants (pages 97–99 and 135 [12]). The hazard associated with transgene expression in the salivary glands is specifically mentioned (page 135 [12]).

The question of whether or not the illustrative concern outlined above represents in reality a genuine allergen hazard to some humans (it quite possibly does not, though this needs to be experimentally tested) is in our opinion not the only question of importance raised by the pre-release regulatory response to field trial applications involving OX513A. A more generally important question is, how could field testing of OX513A progress to the point of large-scale releases into human populated areas with there being any doubt that this fairly obvious hazard had received rigorous scientific consideration? The failure of the regulatory authorities involved to transparently communicate what scientific consideration this hazard received raises the question of how more complex hazards have been dealt with. This lack of clarity is particularly perplexing, as many pertinent facts relating to the above hazard could have easily been established using standard laboratory techniques or caged field trials (and would have been of sufficient interest to warrant publication). The specific concern about partial fertility of OX513A was reportedly submitted to the Malaysian regulatory authority in the pre-approval public consultation period by at least one organization (Third World Network point 2 [89], and presumably in the meetings they had with the Genetic Modification Advisory Committee [64]). However, it is not possible to discern from any pre-release documents that it received any attention.

It is noteworthy that the risk assessment conducted by Beech et al. (2009) clearly makes the assumption that the hypothetical RIDL stocks they are considering are 100% sterile and that no transgenic females are accidentally released (see consideration of potential hazards 20 and 24, respectively, in appendix 1 of [85]). While this may be a reasonable simplifying assumption for a basic teaching exercise, it is not a reasonable assumption for a ground-breaking real-world analysis (particularly where the proposed release stock, OX513A, is reported to be partially fertile and females are to be removed prior to release using a manual method). The impact of this assumption can clearly be seen in one of the two listed potential hazards that mention a deleterious allergic response to being bitten (potential hazard 20, [85]), which only notes that “Males don't bite and take in blood, only females bite”. Ignoring the probable presence of transgenic females in the environment results in all the potential hazards stemming from injection during blood feeding being ignored (the same simplifying assumption is also made in appendix I [90]). Consequently, allergic responses of humans to mosquito bites is implausibly given a lower risk assessment (“very negligible”) than the possibility that the RIDL transgene may cause male mosquitoes to acquire the ability to blood feed (“negligible” potential hazard 1, [85]). This is despite the fact that all males of the over 3,000 known species of mosquitoes are incapable of blood feeding.

Finally, it should be noted that even if it is accepted that there is a pressing need for new approaches to stop insects from spreading human disease, it cannot be reasonably argued that the particular configuration of the RIDL system in OX513A represents the only feasible SIT approach (e.g., [14], [91]) or indeed the only transgenic SIT approach (e.g., [29], [31] or [34], which is an alternative configuration of the RIDL system where tTA expression is limited to flight muscles).

## Credible Risk Assessments of GM Insects Require a Documented, Multi-Disciplinary Approach

The consideration of even the single hypothetical concern above illustrates how important a multi-disciplinary approach can be in assessing environmental risk. While it is essential to consult unbiased molecular biologists to elucidate the properties of the transgenic constructs, it is also necessary to include and document the involvement of immunologists, medical entomologists, and ecologists working in disease-endemic areas. This breadth of skills and knowledge is unlikely to be present in the offices of regulatory agencies and may not even be present in small expert panels.

Admittedly, a more inclusive conventional scientific process is likely to be initially slower than proceeding to field trials with significant voids in understanding. It may, however, ultimately allow more rapid progress by avoiding delays resulting from unnecessary controversy. We consider it self-evident that only through high levels of scientific and public confidence will it ever be possible to create a situation where the granting of GM insect permits can occur in a timely and uncontroversial manner. Furthermore, following the standard publication-based scientific process will enable the large number of developing nations that are reported to be interested in this technology more opportunity to develop systems that are not only suited to their requirements but also under their direct control.

## A Checklist for Assessing the Probable Scientific Quality of Regulatory Release Approvals, Based on Publically Available Pre-Release Documents

Informed consent can provide a basis for trust provided that those who are to consent are not offered a flood of uncheckable information, but rather information whose accuracy they can check and assess for themselves. This is demanding. […] Increasing transparency can produce a flood of unsorted information and misinformation that provides little but confusion unless it can be sorted and assessed. It may add to uncertainty rather than to trust. ([92], see also [93])

Assessing the scientific quality of regulatory decisions can be a daunting prospect even for specialist scientists, and this is also often the case for the public, journalists, and lawmakers. Therefore, we present a checklist in Table 1 to provide a comprehensible starting point with which non-specialists can assess whether regulatory decisions are likely to be of (1) demonstrably high scientific quality, or (2) indeterminate or low scientific quality.

**Table 1:** Checklist for assessing the scientific quality of approvals for un-caged field trials, based on the examination of documents made publically available by regulators prior to the start of releases.

	Likely Features of a Demonstrably High Scientific Quality Release Approval		Likely Features of an Indeterminate or Low Scientific Quality Release Approval	
	-Suitable for less restricted field trials and de-regulation.		-Potentially suitable for limited field trials (but probably not in circumstances likely to involve transgenic insects biting humans)	
□	Complete scientific details of the proposed field trial can be made available during pre-approval public consultations and notifications (e.g., §1500.1.b. [48], article 23 [94], sections 52 & 54 [114], page 18 [115]).	□	Significant scientific details of the proposed field trial cannot be made available during pre-approval public consultations and notifications (most likely at the request of the permit applicant or their collaborators, sections 184 & 185 [114], article 21 [94], pages 8–9 [38]).	
□	Complete list of all potential hazards considered by regulators is published (e.g., sections 52 & 54 [114]), along with their determined risk classification by named individuals.	□	No complete list of potential hazards considered by regulators is published by them, or only a summary is made available (recognizing that publication of only a post-release summary is legally required by the 161 parties to the Cartagena Protocol on Biosafety, article 20.3.c [94]).	
□	A substantial body of relevant interdisciplinary research is cited from multiple independent groups with no serious gaps in areas of importance for assessing potential environmental impact and human health (e.g., pages 43–44 [115], article 15 [94]).	□	Scientific points of importance for assessing potential environmental impact and human health are based on no evidence (e.g., §1502.22. [48]), or a small number of publications from a single laboratory or commercial company.	
□	Documents concentrate on the issues that are truly significant and specific to the case under consideration, rather than the amassing of needless detail. (e.g., page 159 [20], §1500.1.b. [48], page 19 and table 6.3 [115]).	□	Documents are overly generic, use obscure language (e.g., page 160 [20], §1502.8 [48], page 72 [115]), unnecessarily long, or fail to adequately address points of importance for environmental protection or human health.	
□	The majority of data cited in regulatory documents is published, ideally in peer-reviewed journals (e.g., pages 43–44 [115]). No scientific points of fundamental importance for environmental protection or human health rely on unpublished data or no data at all (unless explicitly stated, e.g., §1502.22. [48]).	□	Substantial reference is made to data that is unpublished at the time of the permit application or is published in a form that cannot be publically accessed (e.g., during the time period allowed for public consultations §1502. 21 [48]).	
□	Any prior data, obtained from field trials in other countries, cited in support of permit approval is widely recognized as having been conducted in an ethical manner (e.g., [103],[25],[104],[96], article 23 [94]).	□	Prior data, obtained from field trials in other countries, cited in support of permit approval does not conform to minimal international norms in terms of ethics, public notification, and environmental protection, and was not within an appropriate legal framework.	
□	A protocol is given by regulators that would allow the unique identification of the stocks that have received authorization for release (e.g., integration site sequence section, 2.1.2.4 [75], annex III 9.c [94]).	□	No protocols are provided that would allow the unique identification of the stocks that have received authorization for release; a prerequisite to independent verification that unauthorized stocks were not accidentally released or that transgene integration sites remained stable.	
□	Evidence of a history of access to relevant biological material by independent researchers is apparent or it is indicated that transgenic stocks were deposited at a stock center.	□	No evidence of provisions having been made for allowing access to biological material by independent researchers.	
□	Where a trial involves probable biting of humans by insects expressing transgenic proteins that could be transferred to humans during biting (e.g., due to expression in salivary glands), it is unambiguously and publically documented prior to releases commencing that appropriate experimental allergenicity data has been considered by regulators (e.g., pages 97–99 & 135 [12], [88]).	□	Where a trial involves probable biting of humans by insects expressing transgenic proteins that could be transferred to humans during biting (e.g., due to expression in salivary glands), there is no pre-release documentation that adequate experimental allergenicity data has been considered by regulators.	
□	Information in documents provided by the regulator is clear, understandable, and accurate with respects to all points of fundamental importance for environmental protection and human health (e.g., §1500.1.b. [48]).	□	Information in documents provided by the regulator is unclear (e.g., page 160 [20], §1502.8 [48], page 72 [115]) or inaccurate on points of fundamental importance for environmental protection and human health.	

Citations given in the above checklist are intended to provide for non-specialist readers a small number of relevant passages from national laws, international agreements, scientific literature, or regulatory guidelines.

It is crucial to note that regulatory decisions are never undertaken with complete scientific evidence being available, and this is just as true for GM insects as it is for considerations of new insecticides or building practices. Consequently, a degree of uncertainty in the conclusions of EIAs is inherent and cannot reasonably be used as an absolute argument against granting permission for experimental field trials or applied uses of novel techniques (article 10.6 [94]). Regulators attempt to balance the risks and likely value of novel techniques or proposed experiments through evidence-based analysis of risk.

The checklist in Table 1 is based on the assumption that “risk assessment [of GM insects] should be carried out in a scientifically sound and transparent manner” (annex III [94]), and where regulatory decisions are of high scientific quality it is in the regulators' strategic interest to advertise this prior to releases commencing (assuming they are allowed to do so by permit applicants, see below). Many of the criteria on the checklist relating directly to scientific quality are dealt with in existing international guidelines and statutes (e.g., NAPPO, EFSA, FAO/IAEA, [20], see review [95]). However, those criteria relating to scientific transparency are rarely codified except in domestic legislation (though see [20], [96] and at least five working papers in [22], and see article 23 [94]). Recognizing that a simple dichotomous non-exhaustive checklist may not capture the complexity of every situation, the checklist does provide a starting point with which non-specialists can identify approvals, which most scientists would consider to have a demonstrably high quality scientific basis. It should be emphasized that the checklist is intended to provide an overall assessment of quality, and the inability to check a single box in the left column should not necessarily be seen as a good reason for rejection of a sound scientific basis for approval. Furthermore, it should be noted that many of the criteria in the checklist relate to universal scientific principles, and where there is a degree of subjectivity the public should be able to contact and receive advice from impartial local or international academics in almost any scientific discipline.

If most boxes in the right hand column can be checked, it is reasonable to assume that the scientific basis of release approval is likely to be widely questioned, either on the basis that inadequate pre-release information is being made available, or that available information reflects a genuinely low quality scientific basis for approval. With respect to the former possibility, it is important to note that restrictions on the publication of information is often, quite legally, imposed upon regulators by permit applicants or their collaborators (e.g., [97], see pages 7 and 8 [98]). The extent to which this can interfere with the ability of regulators to meaningfully communicate with the public can be illustrated using US guidelines (page 9 [38]). Permit applicants to the US regulator (BRS-APHIS) can request that fundamental scientific information be excluded from publication by the regulatory authority on the basis that it is confidential business information. Excluded information can “often reasonably [be] justified” to include the identity of transgene donor organisms, transgene names, transgene descriptions, genetic transformation methods, and even the phenotype of the release organism (in some circumstances, even the species name of the proposed release organism and its “phenotypic category” can be withheld, page 9 [38]). Given the breadth of information that can legally be excluded from publication, it can be very difficult for regulators to publish scientifically meaningful information on permit applications. This can fundamentally undermine efforts of regulators to build public confidence, which is often a statutory and strategic objective of regulators around the globe (e.g., points 6 and 12 [67], article 23 [94]). Furthermore, because the configurations of the transgenic constructs that have so far been released are already widely known (e.g., mosquitoes, see [28], [30] and pending patent application PCT/GB2004/003263; pink bollworm, see [99] and Supporting File S4), it is difficult to identify the desired consequences of such restrictions.

Finally, while it appears a minor technical issue, the importance of access to relevant biological material should not be underestimated in stimulating diverse and independent scientific study (Table 1, eighth point on checklist). In the absence of a concerted effort to secure access without onerous restrictions, independent researchers will likely suffer the same restrictions that have limited the unbiased study of commercialized GM crops [100].

## Conclusions

From an historical perspective, it is interesting to note that this is not the first time controversy has arisen around field trials of a genetic pest-control technique (as has previously been pointed out in [15], [19], page 46 [23], and page 12 [24]). In 1975, a World Health Organization (WHO) sponsored field trial in India of an earlier form of non-transgenic genetic pest control collapsed due to a failure to rebut false accusations of exploitative motives by an international funder of the trial [101]. This ultimately led to the abandonment of the entire international WHO program, which had been developed over more than a decade. An article written at the time entitled “Germ-War Allegations Force WHO out of Indian Mosquito Project” makes sobering reading [102] and illustrates that the power of public opinion in the adoption or rejection of new technologies should not be underestimated (pages 11–14 [24]). This was particularly unfortunate, as earlier field trials had shown that the techniques under evaluation had considerable potential and may have played a role in improving or saving an unknown number of lives [15]. In retrospect, the capacity of the WHO to rebut the allegations would have been enhanced by adhering to those principles and benchmarks applied in contemporary clinical trials that are specifically intended to avoid the appearance of exploitative behavior [96], [103], particularly when operating in developing countries [104].

In 2004 it was noted, in the context of GM insects, by professor Paul Thompson (page 12 [24]) that erosion of public confidence in regulators tends to lead to individuals or groups asserting rights to withhold consent for technological development. While this may not initially be recognized by regulatory bodies and permit applicants as a serious strategic problem, assertion of legal opt-out rights can severely hamper technology adoption, particularly where it is difficult or impossible to precisely physically constrain (as is the case for flying GM insects; see also controversy about dispersion of pollen from GM plants). It appears that such a situation may already have arisen, with plans by APHIS to use a transgenic pink bollworm stock expressing a fluorescent marker (OX1138B [105]) in their ongoing suppression programs in Arizona, New Mexico, and California, where the opt-out rights of organic cotton farmers may prevent this (see discussion on pages 17–22 [106]). This is despite 9 years of large-scale publically funded experiments by APHIS, where units within APHIS have acted as both regulator and the sole permit holder. Even though, opt-out rights probably have limited scientific merit (though this still remains to be established in a clear and concise publically available regulatory document) with a well understood fluorescent marker (Ds-red; see the large amount of laboratory and field experimental data in Supporting File S3) in a non-biting insect, on a crop that is not indented for human consumption.

For GM insect technologies as a whole to avoid abandonment before it is possible to determine what value they possess, the perception that accurate and informed public engagement is a means to delay technological development [107] must be rejected (prompt, bold steps by APHIS could prove pivotal). Not least because public acceptance of particular biotechnological techniques can be high when they are perceived to provide advances of real value (e.g., [108], [109]). While it may appear naïve to argue for pre-release access to accurate scientific information and a high quality multi-disciplinary approach, it is in our opinion even more naïve to expect that the development of GM insect technologies will progress far in its absence.

Subsequent to the acceptance of this article, the first peer-reviewed studies about GM insect field trials were published [110], [111].

## Supporting Information

Supporting File S1 Extended glossary to assist non-specialist readers.
(DOC)

Supporting File S2 US regulatory experience 1996–2010.
(DOC)

Supporting File S3 Table of cited literature in 2008-EIS.
(DOC)

Supporting File S4 Documents obtained through Freedom of Information Act request.
(PDF)

Supporting File S5 US Environmental Assessment 2001-EA.
(PDF)

Supporting File S6 Figure 1 data.
(XLS)
