# Supplementary material for: Scientific Standards and the Regulation of Genetically Modified Insects
Source: PLoS Negl Trop Dis. 2012 Jan 31;6(1):e1502. doi: 10.1371/journal.pntd.0001502 (PMC3269433; doi:10.1371/journal.pntd.0001502)
Supplement: Supporting File S1 — Extended glossary to assist non-specialist readers. (DOC) [file pntd.0001502.s001.doc]

**Glossary and abbreviations**

**Allele =** either of a pair (or series) of alternative forms of a gene that can occupy the same locus on a particular chromosome.

**APHIS= Animal and Plant Health Inspection Service (APHIS)** is an agency of the [United States Department of Agriculture](http://en.wikipedia.org/wiki/United_States_Department_of_Agriculture) (USDA)

**Construct =** a synthetic DNA sequence introduced using recombinant molecular DNA techniques into the chromosomes of an organism. Once a genetic construct has been inserted into an individuals or stock it is considered transgeneic.

**Dominant** = A genetic term used to identify traits which are apparent if inherited from just one parent.

**EA**= **Environmental assessment**. A concise public document prepared by a US Federal agency in which the anticipated environmental impact of a proposed action (field trials in the context of this paper) is scientifically considered. These actions are usually of *“limited scope (particular sites, species, or activities) and potential effect (impacting relatively few environmental values or systems).”*. (§372.5b [1])

**EIA= Environmental Impact Assessment(s)**. A generic term for documents prepared by regulatory authorities around the world, which presents evidence-based scientific analysis of the environmental impact of proposed novel actions (also often called environmental risk assessments). The process of environmental impact assessment is commonly divided into four stages: hazard identification, hazard assessment (describing the hazards), exposure assessment (e.g. assessing the probability, likelihood, exposure or frequency of a hazard) and assessment of consequences. In some regulatory process the environmental impacts can be expanded beyond biological impacts to include economic, cultural and social ones. Both EIS and EA documents are types of EIA documents in the US regulatory system.

**EIS** = **Environmental Impact Statement**. A comprehensive public document prepared by a US Federal agency in which anticipated environmental effects of a proposed change in policy or rules is considered. These *“typically involve the agency, an entire program, or a substantial program component and are characterized by their broad scope (often global or nationwide) and potential effect (impacting a wide range of environmental quality values or indicators, whether or not affected individuals or systems may be completely identified at the time).”*. The EIS must be supported by evidence that the agency has made the necessary environmental analyses. The draft EIS is mandated by law to be available for public comment. (§372.5a [1]))

**FOIA= Freedom of Information Act**

**GM= Genetically modified**. Modified in genotype using recombinant molecular DNA techniques.

**OX513A=** a transgenic stock of *Aedes aegypti* mosquitoes which has been transformed with genetic construct of the RIDL system described in [2,3]

**PRP= preventative release program** . Prophylactic use of the sterile insect

technique that serves to limit the probability of rare immigrant females establishing economically significant populations. Very few if any fertile females are encountered by sterile males.

**RDL constructs** = repressible dominant lethal **construct**. Inheritance of an unrepressed dominant lethal transgene from one parent results in death regardless of the genotype of the other parent (genetically **dominant**). Lethality can however be suppressed (i.e. is conditional) through a specific modification of rearing conditions which would not be encountered by the offspring of released males in the environment (e.g. by the provision of dietary tetracycline e.g. [2-5]). With the environmental modification, RDL **transgene** carrying stocks can be stably maintained in the laboratory or mass rearing facilities and still have a lethal effect on the progeny of released individuals in the environment. Where RDL transgenes are engineered so that released males are fully sterile (i.e. released males have no viable offspring) this is a type of SIT. However, RDL **transgenes** can also be engineered to be semi-sterile e.g. female-killing constructs .

**Recessive** = A genetic term indicating that a trait will only be apparent in offspring if it is inherited from both parents.

**SIT= Sterile insect Technique**. Is a species specific pest eradication and suppression technique employed widely across the world. SIT programs generally release large numbers of sterile males (which are innoxious as it is generally only females that are pestiferous) of a target pest species over successive generations at a high enough frequency that the probability of wild females mating with wild fertile males is greatly lowered. If the frequency of matings with sterile males is sufficiently high in the wild target population this can result in a dramatic reduction in the number of viable offspring generated each generation. SIT is a pest management technique that does not involve the dispersion of any chemicals into the environment or the release of any novel species into the environment. Conventional Sterile Insect Technique (SIT), employing ionizing radiation to sterilize male insects, was developed in the 1950s in response to increasing rates of insecticide resistance. In 60 years of application across a diverse range of species there has been a complete absence of reports of the evolution of heritable resistance to the genetic damage caused by radiation based SIT. This absence of resistance is largely explicable in that each sperm has unique combinations of genetic damage caused by high doses of ionizing radiation which makes the evolution of resistance challenging.

**Transgene =** A foreign gene that is stably inserted into the genome of a cell via recombinant DNA techniques.

**US =United States of America.**

**USDA= United States Department of Agriculture.**

1. USDA (2007) APHIS National Environmental Policy Act Implementing Procedures. Federal Register 71.

2. Gong P, Epton MJ, Fu G, Scaife S, Hiscox A, et al., others (2005) A dominant lethal genetic system for autocidal control of the Mediterranean fruitfly. Nature Biotechnology 23: 453–456. doi:10.1038/nbt1071

3. Phuc HK, Andreasen MH, Burton RS, Vass C, Epton MJ, et al. (2007) Late-acting dominant lethal genetic systems and mosquito control. BMC biology 5: 11. doi:10.1186/1741-7007-5-11

4. Horn C, Wimmer EA (2002) A transgene-based, embryo-specific lethality system for insect pest management. Nature Biotechnology 21: 64–70. doi:10.1038/nbt769

5. Horn C, Wimmer EA (2000) A versatile vector set for animal transgenesis. Development Genes and Evolution 210: 630–637.
