# Supplementary material for: Scientific Standards and the Regulation of Genetically Modified Insects
Source: PLoS Negl Trop Dis. 2012 Jan 31;6(1):e1502. doi: 10.1371/journal.pntd.0001502 (PMC3269433; doi:10.1371/journal.pntd.0001502)
Supplement: Supporting File S2 — US regulatory experience 1996–2010. (DOC) [file pntd.0001502.s002.doc]

**US GM insect regulatory experience 1996-2010.**

Recognising the limited of amount of publicly available information we attempt to give a concise summary of the regulatory process GM insects have been subject to in the US.

***1996.*** The first application for release of GM insects was submitted in June 1996 by the University of California, however this was subsequently withdrawn (96-159-01r).

***2001-EIS***. The first mention of GM insects in published regulatory documents that we can find was in a 2001 environmental impact statement (EIS) entitled “*Fruit Fly Cooperative Control Program, Final Environmental Impact Statement—2001*”[1] which stated that *“Biotechnological control methods are currently under development and are not available for program use at this time. Because the circumstances surrounding their uses are uncertain, information on their potential effects upon land, water, or air resources and quality cannot be determined at this time*.” Page 82

***2001-EA.*** *Confined Field Study of a Transgenic Pink Bollworm, Pectinophora gossypiella* [2]. The first permit for a GM insect field trial granted by the regulator BRS-APHIS was in October 2001 (01-029-01r) and was awarded jointly to two other APHIS units, Plant Protection and Quarantine (PPQ-APHIS) and the Center for Plant Health Science and Technology (CPHST-APHIS) at their Decision Support and Pest Management Systems Laboratory in Phoenix. To date all granted permits by the regulator BRS-APHIS have been to other units within APHIS (though it is not possible in most cases to confirm which units from publicly available information) and have been conducted in Arizona. This experiment involved the release of radiation sterilized pink bollworm moths (*Pectinophora gossypiella*, a moth pest of cotton), which expressed a green fluorescent marker, into cages at a site 30km from the Decision Support and Pest Management Systems Laboratory in Phoenix. The released stock had been generated at the University of California and had been described in detail in a peer reviewed article , [3]. Due to the novelty of this, the first caged field experiment with GM insects, an Environmental Assessment (EA) document was prepared by two academics from the University of California and a USDA scientist. The process of drafting involved a 1 month public comment period (Federal register Docket No. 01-024-1). It is interesting to note that the 2001-EA included a section entitled *“Likelihood of controversy of effects on environmental quality”*, while all subsequent regulatory documents did not consider the impact of controversy (page 13 [2]).

***2002.*** The application for the first field trial caused some public controversy and media interest. Two months after granting the permit, APHIS announced in February 2002 its intention to draft a comprehensive EIS document on this subject (Federal register Docket No. 01-124-1).

***2003 & 2004.*** In 2003 and 2004 additional permits were applied for and granted (03-104-01r & 04-118-01r) but this time without the requirement of EA preparation. This is presumably due to the use of a “*categorical exclusion*” (CEs) which allows regulators to rely on an earlier similar EA rather than draft a new one (see discussion in the main text).

***2005-EA.*** *Field Study of Genetically Modified Pink Bollworm, Pectinophora gossypiella* [4]*.* A permit application in 2005 for the free release of radiation-sterilized pink bollworms expressing a green fluorescent marker onto isolated cotton fields was applied for (05-115-01r). This free release experiment was sufficiently different from the caged one described in the 2001-EA so that the use of a CE was not considered appropriate. Consequently, a new EA was written by 2 USDA scientists, with multiple public comment periods being allowed.

***2005-2008.*** Seven further permits were granted between 2005 and October 2008 all using CEs presumably based on the 2001-EA or the 2005-EA documents, with no significant details being made public (see details from example 2010 permit notification in the main text, [5]).

**2008-EIS**. *Use of Genetically Engineered Fruit Fly and Pink Bollworm in APHIS Plant Pest Control Programs* [6]. In December 2006 it was again announced that APHIS was to prepare an EIS on GM insects (almost 4 years after the 2002 announcement). The document was prepared by 9 US government scientists and one Biotechnology Regulatory Consultant/Agent. In October 2008 the EIS was published after a mandated 1 month public comment period. The document covers the potential integration of GM insects into on-going SIT programs run by APHIS in the following 4 agricultural pest species: pink bollworm, the Mediterranean fruit fly (*Ceratitis capitata*), the Mexican fruit fly (*Anastrepha ludens*), and oriental fruit fly (*Bactrocera dorsalis*). The 2008-EIS formed the basis for the announcement in May 2009 that APHIS will “*permit integration of genetically engineered insects into its plant pest control and eradication programs*” (Federal register Docket No. APHIS-2006-0166). Both the 2001-EA and the 2005-EA documents provide detailed environmental impact assessments (EIA) for the fluorescent construct used in the experiments and cite the scientific literature that relates to it, the ecology of the single species involved and the precise experimental details. However, in the 334 pages of the 2008-EIS it is hard to identify any discussion specific enough to indicate what genetic technique is being considered in which species and for what application (4 species and a range of developed and hypothetical GM approaches are considered). The reduced specificity of EIS documents is allowed for in the US regulatory system by the fact that future experimental releases of GM insects (which are considered sufficiently different from those described in the 2001-EA and the 2005-EA) will still require the drafting and publication of new EA documents. This would also presumably be the case for all the techniques which received apparent endorsement in the executive summary of the 2008-EIS (page VI executive summery [6]).

**2008-2010.** No additional regulatory documents have been published. A further 3 permits have been granted using CEs , two permits were applied for and then subsequently withdrawn. At the time of writing one application has remained pending for last 4 months (10-074-102r, [5]*)*.

As far as we can discern there is no impediment to a return to routine publication of permit applications and it is explicitly stated in the BRS-APHIS permit drafting guidelines that all documents submitted to the BRS-APHIS as part of a permit application are subject to the Freedom Of Information Act (FOIA, page 8 [7]). It is also stated that “*BRS voluntarily makes many submitted documents freely available on its website*” (page 8 [7]); however, as far as we can determine, this has not been the case for GM insect applications. Which would appear to contrast with the general commitment of APHIS to creating ”*a period of unprecedented openness in government”* (e.g. FOIA reading room [8]).

1. USDA-APHIS (2001) Fruit Fly Cooperative Control Program, Final Environmental Impact Statement. 385. Available: http://www.aphis.usda.gov/plant_health/ea/downloads/fffeis.pdf.

2. USDA-APHIS (2001) Confined Field Study of a Transgenic Pink Bollworm, Pectinophora gossypiella, Environmental assessment 01-029-01r. 20 p.

3. Peloquin JJ, Thibault ST, Staten R, Miller TA, San S, et al. (2000) Germ-line transformation of pink bollworm ( Lepidoptera : Gelechiidae ) mediated by the piggyBac transposable element. Insect Molecular Biology 9: 323-333.

4. USDA-APHIS (2005) Field Study of Genetically Modified Pink Bollworm, Pectinophora gossypiella. Environmental assessment, 05-115-01r. 70. Available: http://www.aphis.usda.gov/brs/aphisdocs/05_09801r_ea.pdf.

5. BRS-APHIS (2010) BRS-APHIS all release permit applications.. Available: http://www.aphis.usda.gov/brs/status/relday.html. Accessed 1 Dec 2010.

6. USDA-APHIS (2008) Use of Genetically Engineered Fruit Fly and Pink Bollworm in APHIS Plant Pest Control Programs. Final Environmental Impact Statement. 334. Available: //www.aphis.usda.gov/plant_health/ea/downloads/eis-gen-pbw-ff.pdf.

7. USDA-APHIS (2008) General Document Preparation Guidelines For Submission to BRS. 20737: 11. Available: www.aphis.usda.gov/brs/pdf/Doc_Prep_Guidance.pdf.

8. USDA-APHIS (2010) APHIS Makes Redacted FOIA Responses Available on Agency Website. 1. Available: http://www.aphis.usda.gov/publications/aphis_general/content/printable_version/sa_foia_requests.pdf.

**The impact of Intellectual property**

Of the transgenic approaches mentioned in the 2008-EIS the only two RDL construct approaches are the explicit subject of patent applications. The first patent by the Georg-August-Universität Göttingen (PCT/EP2009/053240) makes limited claims [1,2]. It is possible that the second patent by OXITEC Ltd. for a specific configuration the RIDL® system [3] will ultimately have more limited claims than in the original 2003 patent application (PCT/GB2004/003263). This is because the tetracycline controlled positive feedback-loop which forms its core has been previously described in *E. coli* (W0/2001/59088), though this is still a matter of ongoing correspondence between Oxitec Ltd. and the patenting authority (see letter from EPO 14/11/08 and subsequent correspondence https://register.epo.org/espacenet/application?number=EP04743590&lng=en&tab=doclist).

It is however noteworthy that the United States Department of Agriculture (of which APHIS is a division) employs international leaders in the development of GM insect pest control (with numerous important breakthroughs having occurred in their laboratories) and consequently maintains a considerable in-house capacity to regenerate or optimise any techniques that are not patent protected. This capacity could be important if dealing with a third party results in an unacceptable restriction to the flow of scientific information.

Furthermore, on an international level, the prospects for avoiding proprietary impediments to the free flow of scientific data on GM insect techniques at this early stage in its development are still good. For example, the extent to which proprietary considerations could impact the use of fluorescent constructs is limited (even though most fluorescent proteins are patented). There is an increasing ease with which any government or organization could, for a very modest one-off cost, have GM insects developed in an ever expanding range of species (*e.g*. a non-profit insect transformation service at the University of Maryland, College Park, has the capacity to both apply currently developed transformation protocols and develop ones for new species).

1. Horn C, Wimmer EA (2002) A transgene-based, embryo-specific lethality system for insect pest management. Nature Biotechnology 21: 64–70. doi:10.1038/nbt769

2. Schetelig MF, Caceres C, Zacharopoulou A, Franz G, Wimmer E a (2009) Conditional embryonic lethality to improve the sterile insect technique in Ceratitis capitata (Diptera: Tephritidae). BMC biology 7: 4. doi:10.1186/1741-7007-7-4

3. Fu G, Scaife S, Hiscox A (2005) A dominant lethal genetic system for autocidal control of the Mediterranean fruitfly. Nature 23: 453–456. doi:10.1038/nbt1071
