# Supplementary material for: Scientific Standards and the Regulation of Genetically Modified Insects
Source: PLoS Negl Trop Dis. 2012 Jan 31;6(1):e1502. doi: 10.1371/journal.pntd.0001502 (PMC3269433; doi:10.1371/journal.pntd.0001502)
Supplement: Supporting File S3 — Table of cited literature in 2008-EIS. (DOC) [file pntd.0001502.s003.doc]

Table 1

**Published experimental literature cited in the 2008-FEIS [1] grouped by the 5 examples uses described in the executive summary.**

Note that the vast majority of citations are not actually made in the context of discussions of potential environmental impact. Most are cited as a part of technical descriptions of transgenic constructs in appendix C. Consequently the table can be viewed as the most inclusive listing of the cited published experimental literature that could have been used in discussions of environmental impact. Publications which do not present experimental data are not listed e.g. reviews, conference abstracts and regulatory documents.

In example uses 2, 3 & 5 where RDL-transgenic constructs are intended for release, cited studies experimentally examining fitness are identified as this is essential to assessing the value of transgenic approaches relative to established radiation based sterilization.

Note that publications are not uniquely listed and the same publication can be listed as being potentially relevant to more than one example application.

See 2008-EIS for full citations [1].

**( 1) Use of fluorescent marker constructs in radiation sterilized fruit flies.**

*“mass-rearing of either male and female or male-only fruit flies with a marker gene and that are sterilized by radiation exposure and produce practically no offspring,”*

The analysis of published literature on the possible environmental impact of release of transgenic fluorescent markers in insects, is limited in the main body of the text to the citation of a single experimental study (Richards et al 2003 page 121) and the citation of an earlier EA (USDA–APHIS, 2005) which in its appendix V address directly the environmental impact of all cnidarian fluorescent proteins

. Additionally , in response to a question raised during the public comment period (appendix E-6 [1]) a section of the earlier USDA–APHIS, 2005 EA was quoted which describes a literature search in May 2005 that found over 15,000 publications using search terms for green and red fluorescent proteins (2005-EA , page 45 appendix V [2]) “*demonstrating their widespread use as biological markers in organisms from all kingdoms”*. Furthermore, the approval of 58 requests for field testing organisms (mainly plants) with green fluorescent protein since 1997 is quoted as having occurred without incident (2005-EA , page 46 appendix V [2])

**CITED LABORATORY BASED STUDIES INVOLVING FLUORESCENTLY MARKED ORGANISMS.**

**Non-insect**

Richards, H.A., Han, C.-T., Hopkins, R.G., Failla, M.L., Ward, W.W., and Stewart, N., Jr., 2003.

**Insect**

*Order:Lepidoptera*

Tamura, T., Thibert, C., Royer, C., Kanda, T., Eappen, A., Kamba, M., Komoto, N., Thomas, J.- L., Mauchamp, B., and Chavancy, G., 2000.

Peloquin, J.J., Thibault, S.T., Staten, R., and Miller, T.A., 2000.

*Order: Coleoptera*

Berghammer, A.J., Klingler, M., and Wimmer, E.A., 1999

*Order: Orthoptera*

Shinmyo, Y., Mito, T., Matsushita, T., Sarashina, I., Miyawaki, K., Ohuchi, H., and Noji, S., 2004.

*Order:Diptera*

Coates, C., Jasinskiene, N., Miyashiro, L., and James, A., 1998.

Grossman, G., Rafferty, C., Clayton, J., Stevens, T., Mukabayire, O., and Benedict, M., 2001.

Kokoza, V., Ahmed, A., Wimmer, E., and Raikhel, A., 2001.

Allen, M.L., O”Brochta, D.A., Atkinson P.W., and Levesque, C.S., 2001

Grossman, G., Rafferty, C., Fraser, M., and Benedict, M., 2002.

Nolan, T., Bower, T., Brown, A., Crisanti, A., and Catteruccia, F., 2002

Perera, O., Harrell, R., and Handler, A., 2002.

Catteruccia, F., Charles, H., Godfray, J., Crisanti, A., 2003

Allen, M.L., Berkebile, D.R., and Skoda, S.R., 2004

Allen, M.L., and Christensen, B.M., 2004.

Allen, M.L., Handler, A.M., Berkebile, D.R., and Skoda, S.R., 2004

Allen, M.L., and Scholl, P.J., 2005

Koukidou, M., Klinakis, A., Reboulakis, C., Zagoraiou, L., Tavernarakis, N., Livadaras, I., Economopoulos, A., and Savakis, C., 2006.

Marrelli, M.T., Li, C,, Rasgon, J.L., Jacobs-Lorena, M., 2007.

Phuc, H., Andreasen, M., Burton, R., Vass, C., Epton, M., Pape, G., Fu, G., Condon, K., Scaife, S., Donnelly, C.*,* 2007

*Family: Tephritidae*

Brand, A., Manoukian, A., and Perrimon, N., 1994

Brand, A., 1995.

Berghammer, A.J., Klingler, M., and Wimmer, E.A., 1999

**Target Tephritid fruit fly species**

*Ceratitis capitata*

Pane, A., Salvemini, M., Delli Bovi, P., Polito, C., and Saccone, G., 2002.

Dafa”alla, T.H., Condon, G.C., Condon, K.C., Phillips, C.E., Morrison, N.I., Jin, L., Epton, M.J., Fu, G., and Alphey, L., 2006.

Gong, P., Epton, M., Fu, G., Scaife, S., Hiscox, A., Condon, K., Condon, G., Morrison, N., Kelly, D., and Dafa”alla, T., Coleman, P., Alphey, L., 2005.

Fu, G., Condon, K., Epton, M., Gong, P., Jin, L., Condon, G., Morrison, N., Dafa”alla, T., and Alphey, L., 2007.

*Anastrepha ludens*

Condon, K., Condon, G., Dafa”alla, T., Forrester, O., Phillips, C., Scaife, S., and Alphey, L., 2007.

*Bactroceria dorsalis*

Handler, A.M., and McCombs, S.D., 2000

**CITED FIELD TRIALS INVOLVING FLUORESCENTLY MARKED ORGANISMS.**

**non-insect**

“58 approved field permits” listed in USDA–APHIS, 2005

**Insect**

no published studies cited*

**(2) Use of sterilizing RDL constructs in fruit flies.**

*“genetically sterilized male-only fruit flies that have a marker gene, that compete more effectively for mates than radiation-sterilized male insects, and that produce practically no offspring;”*

**Laboratory based studies of transgenic male sterilization**

**Non-Dipteran insects**

no published studies cited

*Order: Diptera*

Phuc, H., Andreasen, M., Burton, R., Vass, C., Epton, M., Pape, G., Fu, G., Condon, K., Scaife, S., Donnelly, C.*,* 2007

*Family: Tephritidae*

**construct description**

Horn, C., and Wimmer, E., 2003.

Scott, M., Heinrich, J., and Li, X., 2004.

**Estimates of fitness**

Horn, C., and Wimmer, E., 2003.

**Target Tephritid fruit fly species**

*Ceratitis capitata*

**construct description**

Gong, P., Epton, M., Fu, G., Scaife, S., Hiscox, A., Condon, K., Condon, G., Morrison, N., Kelly, D., and Dafa”alla, T., Coleman, P., Alphey, L., 2005.

Fu, G., Condon, K., Epton, M., Gong, P., Jin, L., Condon, G., Morrison, N., Dafa”alla, T., and Alphey, L., 2007.

**Estimates of fitness**

no published studies cited

*Anastrepha ludens*

**construct description**

no published studies cited

**Estimates of fitness**

no published studies cited

*Bactroceria dorsalis*

**construct description**

no published studies cited

**Estimates of fitness**

no published studies cited

**(3) Use of female-killing RDL constructs in fruit flies.**

*“ fruit flies that produce only male offspring, which carry a heritable sterility gene resulting in only males with that trait and no female offspring in the field;”*

**Cited laboratory based studies of transgenic sexing construct**

**Non-Dipteran insects**

no published studies cited

*Order: Diptera*

*Family:Tephritidae*

**construct description**

Fryxell, K., and Miller, T., 1995.

Heinrich, J., and Scott, M., 2000.

Scott, M., Heinrich, J., and Li, X., 2004.

**Target Tephritid fruit fly species**

*Ceratitis capitata*

**construct description**

Fu, G., Condon, K., Epton, M., Gong, P., Jin, L., Condon, G., Morrison, N., Dafa”alla, T., and Alphey, L., 2007.

**Estimates of fitness**

no published studies cited

*Anastrepha ludens*

**construct description**

no published studies cited

**Estimates of fitness**

no published studies cited

*Bactroceria dorsalis*

**construct description**

no published studies cited

**Estimates of fitness**

no published studies cited

**Cited laboratory based studies of combined transgenic female-killing and male sterilizing construct**

*Non-Dipteran insects*

no published studies cited

*Order: Diptera*

*Family: Tephritidae*

Fu, G., Condon, K., Epton, M., Gong, P., Jin, L., Condon, G., Morrison, N., Dafa”alla, T., and Alphey, L., 2007.

*Family:Tephritidae*

**construct description**

**no published studies cited**

**Target Tephritid fruit fly species**

*Ceratitis capitata*

**construct description**

Fu, G., Condon, K., Epton, M., Gong, P., Jin, L., Condon, G., Morrison, N., Dafa”alla, T., and Alphey, L., 2007.

**Estimates of fitness**

no published studies cited

*Anastrepha ludens*

**construct description**

no published studies cited

**Estimates of fitness**

no published studies cited

*Bactroceria dorsalis*

**construct description**

no published studies cited

**Estimates of fitness**

no published studies cited

**Cited field studies of combined female-killing and male sterilizing construct**

*Non-Dipteran insects*

no published studies cited

*Order: Diptera*

*Family: Tephritidae*

no published studies cited

*Target Tephritid fruit fly species*

*Ceratitis capitata*

no published studies cited

*Anastrepha ludens*

no published studies cited

*Bactroceria dorsalis*

no published studies cited

**(4) Use of fluorescent marker constructs in radiation sterilized pink bollworm.**

*“mass-rearing of male and female pink bollworm that have a marker gene and that are sterilized by radiation before field release;”*

See example 1 for full details of cited experimental studies using fluorescent markers.

**cited experimental laboratory studies of fluorescent markers in Lepidoptera**

*Order:Lepidoptera*

Tamura, T., Thibert, C., Royer, C., Kanda, T., Eappen, A., Kamba, M., Komoto, N., Thomas, J.- L., Mauchamp, B., and Chavancy, G., 2000.

**Cited studies in target species**

*Pectinophora gossypiella*

Peloquin, J.J., Thibault, S.T., Staten, R., and Miller, T.A., 2000.

Miller, E., Staten, R.T., Claus, J., Sledge, M., Peloquin, J., and Miller, T., 2001.

**cited field studies of fluorescent markers in Lepidoptera**

*Order:Lepidoptera*

no published studies cited

**Cited studies in target species**

*Pectinophora gossypiella*

no published studies cited*

**(5) Use of sterilizing RDL constructs in pink bollworms.**

*“ mass-rearing of male and female pink bollworms that are genetically sterile without radiation exposure and that results in males that are more competitive in mating with wild female bollworms than radiation-sterilized male bollworms.”*

see example 2 for cited studies involving male sterilizing transgenes in Dipteran flies

**cited experimental laboratory studies of male sterilizing transgenes in Lepidoptera**

*Order:Lepidoptera*

**construct description**

no published studies cited

**Estimates of fitness**

no published studies cited

**Cited studies in target species**

*Pectinophora gossypiella*

**construct description**

no published studies cited

**Estimates of fitness**

no published studies cited

**cited field studies of male sterilizing transgenes in Lepidoptera**

*Order:Lepidoptera*

no published studies cited

**Cited studies in target species**

*Pectinophora gossypiella*

no published studies cited*

- See discussion of the unpublished field trial in main body text

1. USDA-APHIS (2008) Use of Genetically Engineered Fruit Fly and Pink Bollworm in APHIS Plant Pest Control Programs. Final Environmental Impact Statement. U.S Department of Agriculture, Animal and Plant Health Inspection Service. 334 p.

2. USDA-APHIS (2005) Field Study of Genetically Modified Pink Bollworm, Pectinophora gossypiella. Environmental assessment, 05-115-01r. U.S Department of Agriculture, Animal and Plant Health Inspection Service. 70 p.
