# Supplementary material for: Scientific Standards and the Regulation of Genetically Modified Insects
Source: PLoS Negl Trop Dis. 2012 Jan 31;6(1):e1502. doi: 10.1371/journal.pntd.0001502 (PMC3269433; doi:10.1371/journal.pntd.0001502)
Supplement: Supporting File S4 — Documents obtained through Freedom of Information Act request. (PDF) [file pntd.0001502.s004.pdf]

S4 Documents obtained through freedom of information request (.pdf)

This documents were received on the 2nd of June 2010 as a single un-editable file in response to the FOIA request copied below.

From: "Floyd A. Reed" [redacted]
Date: 30 June, 2008 18:38:51 GMT+02:00
To: foia.officer@aphis.usda.gov
Subject: FOIA Request

Hello,

We are trying to locate documentation on permit 05-118-01r regarding the following release:

05-118-01r United States Department of Agriculture/Animal and Plant Health Inspection Service
Reg article: Pink bollworm
OO-Autocidal
OO-Tetracycline Responsive
Releases in: AZ
Release
Received: 28-APR-2005
Status: Issued 02-AUG-2005

Thanks for any info,

Floyd

Floyd A. Reed, Ph.D.
Max-Planck-Institut fuer Evolutionsbiologie
Abteilung Evolutionsgenetik
August-Thienemannstrasse 2
24306 Ploen (Germany)

June 28, 2005

Mr. David Madison
Arizona Department of Agriculture
1688 W. Adams St.
Phoenix, AZ 85007

Dear Mr. Madison:

This letter accompanies permit application No. 05-118-01r submitted by Dr. Gregory S. Simmons, USDA, AHIPS, PPQ, Center for Plant Health Science and Technology, DSPMSL, Phoenix Plant Protection Center, 3645 East Wiser Avenue, Phoenix, Arizona, in collaboration with (b)(6) of the Center for the purpose of doing cage-contained studies of transgenic pink bollworms (PBW) on cotton. This work is similar to the work done under USDA APHIS permits No. 03-104-01r and 01-029-01r. An Environmental Assessment (EA) under the national Environmental Policy Act was conducted and a Finding of No Significant Impact (FONSI) was reached regarding to make the decision to issue APHIS permits No. 03-104-01r and 01-029-01r. Because an EA has already been conducted for these preceding permit applications, and the permit research proposed in application No. 05-118-01r submitted by Dr. Simmons is similar and equivalently contained, criteria for an EA are no longer met under 7 CFR 372.5 (d) (4) “When a confined field release of genetically engineered organisms or products involves new species or organisms or novel modifications that raise new issues”. It is therefore not required to conduct another EA for this current permit application. There are no claims of confidential business information in any of the documentation.

PBW is one of the most destructive pests of cotton in the world. It was first found in the United States in 1917 and has become a pest in Texas, New Mexico, Arizona and California. Costs relating to prevention, control and yield losses have been estimated by the National Cotton Council to exceed $24 million annually. The San Joaquin Valley of California remains the last cotton growing area in the Southwest that is not generally infested with PBW. Prevention of its establishment in this valley is attributed primarily to the ongoing Sterile-Insect Technique (SIT) program established jointly in 1968 by APHIS, California Department of Food and Agriculture, and the California cotton growers.

An objective of the proposed research of this permit application is to develop a strain of PBW expressing coelenterate-derived Fluorescent Protein (EGFP or DsRed) marker genes and an autocidal effector gene construct (from Oxitec, Oxford, UK). The latter transgene will fatally disrupt the development of insects carrying this gene (particularly the progeny of mating between transgenic insects and wild type insects) when these insects are not supplied with a specific small molecule repressor (a tetracycline derivative). These cage studies are designed to test the function and effectiveness of autocidal transgenes and to determine the effectiveness of these autocidal insects in reducing experimental populations in a fully-contained experiment. Also, this experiment will compare the mating biology of these fluorescent-protein-producing, autocidal insects

to that of wild type colony insects and irradiated wild type colony insects in a fully contained experimental environment. Implementation of genetically marked autocidal insects (with fluorescent proteins) into a PBW mass-rearing SIT program could provide a more effective alternative to irradiation or could reduce the necessary radiation dose to implement SIT, thereby increasing the effectiveness of an already demonstrably successful control program for the exotic pest, PBW. Finally, a genetically marked insect will be a useful monitoring tool for field managers to determine the distribution of treated PBW and to gauge the most efficient means of doing so.

Initial studies with males and females will be conducted in 3.6 x 7.3 x 1.8 m screen field cages placed over cotton plants at the CPHST rearing facility. The site is surrounded by an 8 foot chain link fence, topped with razor wire with locked gates, video surveillance and limited entry authorization. It is at least 3 miles from the nearest cultivated cotton. Adults, though capable of flight, will be contained in field cages. The structure of the field cages is 2.54 cm galvanized pipe covered with Lumite™ Saran™ 20 x 20 mesh fiberglass screen with reinforced corners to prevent tears. This mesh is tighter than mesh used in previously contained studies and as such is even less likely to allow escapes of contained moths than the materials used in USDA APHIS permits No. 03-104-01R and 01-029-01R. Though the adult moths cannot burrow, the cages also have a 30.5 cm plastic skirt running along the bottom that is buried in the soil to prevent moth escapes through soil cracks. An alternative site for confined studies is the same site used under permit 01-029-01R. This alternative site is surrounded by a 6 foot chain link fence.

Escape from such field cages is highly unlikely barring a major weather catastrophe, which itself is likely to destroy the contained insects. Eight pheromone traps baited with 2 mg of Gossyplure™ will be strategically distributed around the cage area to capture any males that might escape from the cages. All cotton plants in the area will be contained in the cages. Once the experimental work is done, all fruiting forms on the cotton will be removed and frozen at – 20°C for 24 hrs to eliminate all PBW life stages.

PBW control strategies will be in place and ready for deployment. They include pesticides and application equipment that have been used or are currently being used to contain and/or control PBW populations. The implementation of these strategies around the field cages and pheromone monitoring and control traps will make the risks negligible for the transgenic strain of PBW to transfer its genetic components to a field population of PBW or of this strain becoming established in the field. Risk is further minimized by research which has established that laboratory rearing of over 74 generations of the transgenic PBW strain give no indication that a transgenic EGFP strain has any competitive advantage over the strains currently maintained in the pink bollworm rearing facility.

Transgenic PBW that are no longer needed will be disposed of by freezing at -20° C for 48 hours. This will destroy any life stage of this insect. All plant fruiting forms in the release cages

will be frozen at -20° C for 24 hours when the study is completed. This treatment will destroy PBW life stages that may infest the fruiting forms.

Previously conducted experiments demonstrated there are no transposases in the PBW genome that mobilize *piggyBac* transposon. This fact is addressed in the EA that has already been conducted resulting in the decision to issue APHIS permits No. 03-104-01R and 01-029-01R. Since there is no identifiable direct effect of this field test on any wild plant or animal species, there is no apparent risk to any threatened or endangered species. The proposed experiments are not expected to cause any adverse environmental effects due to their physical and biological containment. PWB also has no sexually compatible relatives in the United States with which it could reproduce or hybridize.

The application was submitted pursuant to regulations found in 7 CFR Part 340 which regulate the importation, interstate movement, or release into the environment of geneticat1y engineered plant pests. The regulations require that a person obtain a permit from APHIS prior to introducing a regulated article. This letter serves to give notice to and affords the State of Arizona the opportunity to indicate concurrence or non-concurrence with APHIS' assessment that contained field testing of these genetically modified insects does not pose a plant risk. You may also provide any conditions that may be mandated by your State. Please review the enclosed documents and return acknowledgement, associated comments, or reasons for non-concurrence (if applicable) to APHIS within 30 days from the date of this letter or preferably sooner (please use the enclosed form; use additional sheets for response, if needed).

Please refer to permit No. 05-118-01r in your correspondence regarding this application. If you have any questions about this application, please contact me at (301) 734-5720, facsimile (301) 734-8669, or e-mail: john.j.peloquin@aphis.usda.gov.

APHIS hopes to maintain its excellent working relationship with your State and encourages your participation and comments prior to our final decision regarding this permit application.

Sincerely,

John J. Peloquin, Ph.D.
Supervisory Biotechnologist/Entomologist
Animals Branch Chief
Biotechnology Regulatory Services

Enclosures:
Permit Application No. 05-118-01r
State Response Form

Cc:
S. Wellstood, Compliance Branch, Rivderdale, MD 20737
File 05-118-01r

APHIS:BRS:JP:hll:x8231:6/27/2005:0511801r

SUPPLEMENTAL PERMIT CONDITIONS
05-118-01R

Reviewed/Approved: 7/29/05 JPP
REVISED/CORRECTED: 8/6/2005 JPP

1. Studies with males and females will be conducted in 3.6 x 7.3 x 1.8m screen field cages placed over cotton plants at the CPHST rearing facility.

2. The site will be surrounded by an 8 foot chain link fence, topped with razor wire with locked gates, video surveillance and limited entry authorization. It is at least 3 miles from the nearest cultivated cotton. The structure of the field cages is 2.54 cm galvanized pipe covered with a 16 x 16 mesh (256 openings per square inch) fiberglass screen with reinforced corners to prevent tears. The cages also have a 30.5 cm plastic skirt running along the bottom that is buried in the soil to prevent moth escapes.

3. An alternative site for confined studies is the same site used under permit 01-029-01r. This alternative site is surrounded by a 6 foot chain link fence.

4. Eight pheromone traps baited with 2 mg of Gossyplure™ will be strategically distributed around the cage area to capture any males that might escape from the cages. All cotton plants exposed to transgenic moths will be contained in the cages. Once the experimental work is done, all fruiting forms on the cotton will be removed and frozen at -20◦ C for 24 hours to eliminate all PBW life stages.

5. PBW control strategies should be in place and ready for deployment. They will include pesticides and application equipment that have been used or are currently being used to contain and/or control PBW populations.

6. Transgenic PBW that are no longer needed will be disposed of by freezing at -20◦ C for 24 hours. This will not destroy any life stage of this insect. All plant fruiting forms in the release cages will be frozen at -20◦ C for 24 hours when the study is completed. This treatment will destroy PBW life stages that may infest the fruiting forms.

4|28

This application is authorized by the Federal Plant Pest Act (7 U.S.C. 150aa et seq. and the Plant Quarantine Act (7 U.S.C. 151 et seq.)). The information will be used to determine eligibility to receive all types of permits. No permit shall be issued until this application has been approved.

See reverse side for additional information

FORM APPROVED OMB NO. -579-0085

U.S. DEPARTMENT OF AGRICULTURE
BIOTECHNOLOGY, BIOLOGICS, AND ENVIRONMENTAL PROTECTION

APPLICATION FOR PERMIT OR COURTESY PERMIT UNDER 7 CFR 340

(Genetically Engineered Organisms or Products)

INSTRUCTIONS: Complete this form and enclose the supporting materials listed on the reverse side. See page 3 for detailed instructions.

1. NAME AND ADDRESS OF APPLICANT

Gregory S. Simmons, Ph.D.
USDA-APHIS-PPQ-CPHST-DSPMSL
3645 E. Wier Ave
Phoenix, AZ 85040

2. PERMIT REQUESTED ("X" one)

- [ ] Limited - Interstate Movement
- [ ] Limited - Importation
- [x] Release into the Environment
- [ ] Courtesy Permit

3. THIS REQUEST IS ("X" one)

- [x] New
- [ ] Renewal
- [ ] Supplemental

4. TELEPHONE NUMBER

Area Code (602) 437-1295 05-118-01r

5. MEANS OF MOVEMENT

- [ ] Mail
- [ ] Common Carrier (FedEx)
- [ ] Baggage or Handcarried

By whom ______

6. GIVE THE FOLLOWING (IF APPLICABLE) (IF MORE SPACE IS NEEDED ATTACH ADDITIONAL SHEET)

	Scientific Name	Common Name	Trade Name	Other Designation
a. Donor Organism:	Aequorea victoria (jellyfish), Discosoma sp. (coral) Drosophila melanogaster (vinegar fly), Escherichia coli (bacterium), Herpes simplex (virus); OR, alternatively: Oryctolagus cuniculus (rabbit), Bombyx mori (silk moth), Aequorea victoria (jellyfish), Discosoma sp. (coral) Drosophila melanogaster (vinegar fly), Esherichia coli (bacterium), Simaian virus 40 (SV40)-(virus), Cytomegalovirus (CMV),(virus), Herpes simplex (virus), Synthetic.			
b. Recipient Organism:	Pectinophora gossypiella	(pink bollworm)	N/A	N/A
c. Vector or Vector Agent:	piggyBac	N/A	N/A	N/A
d. Regulated Organism or Product:	Pectinophora gossypiella	(pink bollworm)	N/A	N/A
e. If product, list names of constituents:	N/A	N/A	N/A	N/A

7. QUANTITY OF REGULATED ARTICLE TO BE INTRODUCED AND PROPOSED SCHECULE AND NUMBER OF INTRODUCTIONS

Multiple releases of 3,600 adults/wk for up to 20 wks for a total of 72,000 insects.

8. DATE (or inclusive dates of period) OF IMPORTATION, INTERSTATE MOVEMENT, OR RELEASE

For one year, intermittent year approval of permit

9. COUNTRY OR POINT OF ORIGIN OF THE REGULATED ARTICLE

Parent stock USDA-Phoenix,AZ transformed at the University of California or at Oxitech LTD, Oxford, UK.

10. PORT OF ARRIVAL, DESTINATION OF MOVEMENT, OR SPECIFIC LOCATION OF RELEASE

Contained Field Cage Trial in Phoenix, AZ

11. ANY BIOLOGICAL MATERIAL *(e.g., culture medium, or host material)* **ACCOMPANYING THE REGULATED ARTICLE DURING MOVEMENT**

N/A.

12. APPLICANTS FOR A COURTESY PERMIT - STATE WHY YOU BELIEVE THE ORGANISM OR PRODUCT DOES NOT COME WITHIN THE DEFINITION OF A REGULATED ARTICLE

N/A.

13. SEE REVERSE SIDE

I hereby certify that the information in the application and all attachments is complete and accurate to the best of my knowledge and belief.

False Statement: Falsification of any item on this application may result in a fine of not more than $10,000 or imprisonment for not more than 5 years or both. (18 U.S.C. 1001)

14. SIGNATURE OF RESPONSIBLE PERSON

[signature]

15. PRINTED NAME AND TITLE

Gregory S. Simmons, Ph.D.

16. DATE

04/27/2005

FOR APHIS USE ONLY

State Notification Letter Sent	State Review Received	Permit Issued: [ ] Yes [ ] No
Date of Determination	Permit No. 05-118-01r	No. of Permit Labels Issued
		Supplemental Conditions Enclosed: [ ] Yes [ ] No
Signature of BBEP Official	Date	Expiration Date

ENCLOSURES	ENCLOSED ("X")	IF PREVIOUSLY SUBMITTED, LIST DATE & PERMIT NO.
a. Names, addresses, and telephone numbers of the persons who developed and/or supplied the regulated article.	X	
b. A description of the anticipated or actual expression of the altered genetic material in the regulated article and how that expression differs from the expression in the nonmodified parental organism (e.g., morphological or structural characteristics, physiological activities and processes, number of copies of inserted genetic material and the physical state of this material inside the recipient organism (integrated or extrachromosomal), products and secretions, growth characteristics).	X	
c. A detailed description of the molecular biology of the system (e.g., donor-recipient-vector) which is or will be used to produce the regulated article.	X	
d. Country and locality where the donor organism, recipient organism, and vector or vector agent were collected, developed and produced.	X	
e. A detailed description of the purpose of the introduction of the regulated article including a detailed description of the proposed experimental and/or production design.	X	
f. A detailed description of the processes, procedures, and safeguards which have been used or will be used in the country of origin and in the United States toprevent contamination, release, and dissemination in the production of the: donor organism; recipient organism; vector or vector agent; constituent of each reglated article which is a product; and regulated article.	X	
g. A detailed description of the intended destination (including final and all intermediate destinations), uses, and/or distribution of the regulated article (e.g., greenhouses, laboratory, or growth chamber location; field trial location, pilot project location; production, propagation, and manufacture location; proposed sale and distribution location).	X	
h. A detailed description of the proposed procedures, processes, and safeguards which will be used to prevent escape and dissemination of the regulated article at each of the intended destinations.	X	
i. A detailed description of the proposed method of final disposition of the regulated article.	X	

Public reporting burden for this collection of information is estimated to average 5 hours per response, including the time for reviewing instructions, searching existing data souces, gathering and maintaining the data needed, and completing and reviewing the collection of information. Send comments regarding this burden estimate or any other aspect of this collection of information, including suggestions for reducing this burden, to Department of Agriculture, Clearance Officer, OIRM, Room 404-W, Washington, D.C. 20250; and to the Office of Information and Regulatory Affairs, Office of Management and Budget, Washington, D C. 20503.

ENCLOSURE A

Names, addresses, and telephone numbers of the persons who developed and/or supplied the regulated article.

(b)(6)
Department of Entomology
University of California
Riverside, CA 92521
(b)(6)

(b)(6)
Oxitec Ltd
71 Milton Park
Abingdon
Oxford, OX14 4RX
UK
(b)(6)

(b)(6)
Decision Support & Pest Management Systems Laboratory
3645 E Wier Avenue
Phoenix, AZ 85040
(b)(6)

Dr. Gregory Simmons
Entomologist
Decision Support & Pest Management Systems Laboratory
3645 E Wier Avenue
Phoenix, AZ 85040
602–437-1295 x223

(b)(6)
Pink Bollworm Rearing Facility
3645 East Chipman
Phoenix, AZ 85040
(b)(6)

ENCLOSURE B

A description of the anticipated or actual expression of the altered genetic material in the regulated article and how that expression differs from the expression in the nonmodified parental organism.

The additional genetic material in the pink bollworm comprises several protein coding regions:

1. The marker.

This allows the expression of a fluorescent protein (e.g. GFP, DsRed) originally derived from the jellyfish *Aequoria victoria* or from a coral (e.g. *Discosoma* sp.). The transgenic pink bollworm with the marker gene fluoresces when excited by illumination of the appropriate wavelength. These fluorescent proteins, which have been used as markers in a wide range of vertebrate and invertebrate species, confer no known competitive advantage or disadvantage to the recipient, and no ecological or other consequences resulting from incorporation of these markers into the transgenic pink bollworm can be envisioned. The unmodified pink bollworm is not strongly fluorescent, expression of a fluorescent protein therefore allows the modified pink bollworm to be distinguished from unmodified.

2. Tetracycline-repressible transcriptional activator (tTA).

tTA protein binds to and activates expression from the tetracycline response element (tRE), which includes multiple copies of the specific DNA sequence to which tTA binds (tetO). tTA also binds tetracycline with high affinity; the tetracycline bound form of tTA does not bind DNA. tTA therefore acts as a tetracycline regulated switch - in the absence of tetracycline it will induce expression from tRE, whereas in the presence of tetracycline it will not. High level expression of tTA is thought to be deleterious to cells as it can repress their normal transcription; low level expression has no known effect other than activation of tRE. tTA is a synthetic fusion of the tetR protein from *Escherichia coli* with VP16 from herpes simplex virus. TetR provides the tetracycline-repressible sequence-specific DNA binding property, while VP16 is a eukaryotic transcriptional activator. tTA has been used in fungi, plants, mice, mammalian culture cells, the vinegar fly *Drosophila melanogaster*, and the Mediterranean fruit fly *Ceratitis capitata*, with no known adverse effects on the environment or on human health. Unmodified pink bollworm do not have a tTA gene or similar activity.

3. Effector gene.

The effector gene encodes an insect protein or RNA, or fragment thereof, expression of which is predicted to be deleterious to the insect. For example, in the case of the LA476 construct Nipper is the central domain of the Drosophila melanogaster Nipp1Dm gene. This binds to and inhibits the catalytic subunit of type 1 serine/threonine protein phosphatase (PP1c). PP1c is an essential enzyme, therefore high level expression of Nipper (or Nipp1Dm) kills the cell. In the modified pink bollworm, Nipper is under the transcriptional control of tRE, and so is expressed when tTA is present and tetracycline is not. Unmodified pink bollworm are thought to have a Nipp1-like gene, as this protein is present in other insects, the nematode *C. elegans* and mammals.

In the case of the LA1124 construct, tTA is placed under the transcriptional control of tRE, here tTA may itself act as an effector protein. Basal expression of tTA in the modified pink bollworm is predicted to have no visible effect on the modified pink bollworm under normal laboratory rearing conditions in the presence of tetracycline. High level expression of tTA in the absence of tetracycline is predicted to be deleterious to the moth, leading to a competitive disadvantage.

No piggyBac transposase activity nor any antibiotic or pesticide resistance is conferred to the transgenic pink bollworm by the introduced genetic material.

ENCLOSURE C

A detailed description of the molecular biology of the system that was used to produce the regulated article.

PiggyBac is a DNA (deoxyribonucleic acid) transposable element that, only when its ITRs (Inverted Terminal Repeats) are intact, is capable of integrating DNA flanked by element specific DNA into other DNA through mediation of a transposase encoded by an ORF (Open Reading Frame) within the element. In the construct used for transformation of the pink bollworm, the transposase gene of the piggyBac element was irreversibly destroyed by deletion of a section of the transposase gene. Transformation was effected by introducing with the transforming construct a helper plasmid that supplied transposase activity but was itself unable to transpose into other DNA. This transposition defective helper plasmid has an ORF (Open Reading Frame) encoding piggyBac transposase under the control of the *Drosophila melanogaster* hsp70 promoter. One of the inverted terminal repeats that flank the wild type piggyBac transposase in piggyBac has been removed in the helper plasmid so that the helper plasmid cannot, itself, integrate even though it encodes for active piggyBac transposase.
The potential for instability and unwanted mobilization of piggyBac derived transforming constructs must be addressed as follows. It could be argued that if there were endogenous, piggyBac-like elements in pink bollworm, they might provide a source of transposase that could mobilize transgenes flanked by piggyBac derived ITRs. Demonstration of elements homologous to piggyBac in the recipient organism, pink bollworm, might then suggest caution regarding stability of the transgene. However, the DNA mediated element, Hermes, has been used to successfully transform *Aedes aegypti* with little or no evidence of instability of the transgenes over at least 10 generations, even though there are in *Aedes aegypti* endogenous elements (presumably hAt-like as is Hermes) with close enough homology to Hermes so that these endogenous hAt and Hermes-like elements are detected even in higher stringency Southern blots with a Hermes probe. Similarly, piggyBac has been used successfully to transform the Oriental fruit fly, *Bactrocera dorsalis*, with no evidence of instability of the transgenes, even though closely related piggyBac-like elements were later found to be present in that species.

In the case of pink bollworm, low stringency Southern blot experiments on pink bollworm DNA with radio labeled DNA probes derived from piggyBac, which would be even more likely to detect elements with low homology to piggyBac than the higher stringency methods used in Jasinskiene, et al., 1998, were unable to detect any endogenous piggyBac-like elements. This suggests that there are no elements in pink bollworm that might reasonably be expected to mobilize a piggyBac derived transgene. In addition, excision and transposition assays were performed in pink bollworm embryos with piggyBac. This was primarily to determine if piggyBac could integrate into the pink bollworm genome. However, our results showed no transposition of piggyBac in the absence of exogenous piggyBac transposase in these transposition assays, strongly suggesting there were no unknown piggyBac-like elements in the pink bollworm genome capable of mobilizing non-autonomous piggyBac elements. We can thus be reasonably certain there would not be unexpected interactions between the components of the pink bollworm genome and the transforming construct that would result in instability of the transgenes. In any event, experiments to be performed in Phoenix after transfer of the transformed pink bollworm strains will further demonstrate the stability of the transgenes.

REFERENCES

Jasinskiene, N., Coates, C.J., Benedict, M.Q., Conrel, A.J., Rafferty, C.S., James, A.A., Collins, F.H.: Stable transformation of the Yellow Fever Mosquito, *Aedes aegypti*, with the hermes element from the housefly. Proceedings of the National Academy of Sciences of the United States of America, 1998 March 31, 95(7):3743-7.

ENCLOSURE D

Country and locality where the donor organism, recipient organism, and vector or agent were collected, developed and produced:

The United Kingdom, Oxford, the University of Oxford is where all final engineering of the transforming constructs were performed. The genes used from the donor organism and the piggyBac derived portions of the vectors used to build the transforming construct were cloned at this location.
The recipient organism—the pink bollworm, *Pectinophora gossypiella*—is an invasive insect whose origin is uncertain. It is not a native species of the Western Hemisphere though it is now endemic to the southwestern United States and Mexico, associated with commercial cotton production. Introduction of the pink bollworm into the United States appears to have been via infected cottonseed. The pink bollworm appeared in Hearn, TX in 1917, and within a decade it had spread across western Texas, New Mexico, and into Arizona by 1929. The colonies transformed at the University of California Riverside and at Oxford University, Oxford UK originated from the Pink Bollworm Rearing Facility in Phoenix, Arizona.

ENCLOSURE E

A detailed description of the purpose for the introduction of the regulated article including a detailed description of the proposed experimental design.

Pink bollworm (PBW) infestations cost U.S. cotton producers $47 million per year for direct losses and control measures (National Cotton Council, March 2005, unpublished brief). APHIS is involved in two PBW control projects using the release of sterile (SIT) PBW, *Suppression* in the Central Valley of California, and *Eradication* along with *B.t.* cotton, pheromones, and pesticides. The use of SIT will expand to 90,000 acres in 2005 when sterile releases are made in eradication program areas in Texas, New Mexico and the Juarez Valley in Mexico.

The SIT suppression program has been effective and kept the San Joaquin Valley free of PBW for 30 years at low cost. However, increased cotton production costs, worldwide competition and the increasing demands for the expanded PBW eradication program requires a more effective and lower cost program. A major limit on the efficacy of SIT as a control measure is the effect of sterilizing radiation on insect performance. Radiation has a great effect on the competitiveness and effectiveness of lepidoptera used in SIT programs and has been associated with decreased quality, competitiveness, and dispersal ability in many species (North 1975, Carpenter 1997, Bloem 1999) and lower dose radiation is associated with increased mating ability and superior sperm competitiveness (Carpenter 1997). In pink bollworm, the effects of radiation include reduced longevity, decreased sperm transfer by males, decreased sperm

receptivity, decreased female attractiveness and decreased control efficacy (Graham et al. 1972, Flint et al. 1973, Flint et al. 1974, Flint et al. 1977, Bartlett 1978, Miller et al. 1994).

Using genetic engineering to improve PBW control technology could achieve savings and greater program efficacy with the development of a PBW strain with an autocidal or conditionally lethal gene (Fryxell 1995, Miller et al. 1997), which would eliminate the need for irradiating released insects and greatly improve the performance and longevity of released moths.

The goal of this project is to develop a pink bollworm with a conditionally lethal gene using RIDL technology (**R**elease of **I**nsects with a **D**ominant **L**ethal gene, see Thomas 2000, Peng 2005) to make use in an innovative genetic control technique known as autocidal biological control. Progeny carrying a RIDL gene die when the antibiotic chlortetracycline (CTC) is absent. CTC is a normal ingredient in the PBW mass-rearing so a PBW strain with conditionally lethality controlled by CTC is a good choice for this rearing system. This is one of the most promising autocidal control systems in development.

The purpose of this experiment is to test the function of a conditionally lethal pink bollworm based on the RIDL technology in the more realistic conditions of the cotton plant within a quarantine field cage. Current RIDL strain pink bollworm express lethal phenotypes of 60-100% when reared without tetracycline on artificial diet in laboratory rearing conditions, this rate of mortality is expected to increase under the more challenging conditions of a real plant exposed to the stress of a changing environment with the extremes in temperature in the field that are unlike laboratory conditions.

The other goal of this experiment is to estimate the reduction of a wild pink bollworm population caused by release of a RIDL pink bollworm. This treatment will be compared to the reduction in wild pink bollworm caused by release of the standard APHIS strain moth irradiated at 20 kilorads.

Data from these experiments are needed for the next phase of development of a conditionally lethal pink bollworm moth. It is critical to determine if differences between the standard SIT release and RIDL release exist. If the RIDL insect is not as good as the SIT moth, is the difference within the range of improvement possible through outcrossing and strain improvement? If the two treatments have similar control efficacy, or if the RIDL insect is more efficacious, could RIDL release rates be reduced? Lastly, data from these experiments will provide key information about the differences between the two kinds of control technology (RIDL and irradiation) that will be needed for environmental analysis of the project that may be required under the National Environmental Policy Act (NEPA).

There are three experiments planned for testing the function of RIDL pink bollworm by releasing in quarantine field cages:

Experiment 1

This experiment is designed to simulate the season long release of RIDL and 20 KR irradiated moths against a native pink bollworm population within the range of densities that would be encountered within the eradication program.

There are three treatments:

1) Release of RIDL pink bollworm adults with the LA1124 construct, as heterozygotes, homozygotes or doubly homozygous for two separate insertions of the LA1124 construct (e.g., LA1124A & LA1124B).
2) Release of APHIS strain pink bollworm adults irradiated at the standard dose of 20 kilorads.
3) A no release control.

There are five replicates of treatments 1 and 2 and 4 replicates of treatment 3 arrayed in randomized incomplete blocks. Each replicate consists of a cotton plot of four rows of cotton grown in a plot measuring 7 m x 3.5 m wide. Cotton will be planted in mid to late April when soil temperatures are appropriate for germination. After the cotton has reached the 4-8 leaf stage, each plot will be covered with a 3.6 x 7.3 x 1.8 m tall screen cage (Lumite Saran 20 x 20 mesh) placed over a one inch diameter galvanized steel pipe frame. Each cage is fitted with a double door entry and each door is secured with both a heavy duty brass zipper and Velcro flap. The side of the cages are secured to the ground by burying 0.5 m of the side flap within a 0.5 m deep trench.

When the cotton reaches the pin square stage, 20 mating pairs of wild pink bollworm moths will be released into each cage to establish the test target population. Two days later, the first release of RIDL moths and 20KR APHIS moths will be released at the rate of 600 moths (1:1 sex ratio) per cage. No further releases will be made until just before the first generation (F1) of wild PBW moths emerges, this will be estimated by a degree day model and by sampling cotton flowers to determine developmental status. Upon emergence of the F1 generation releases of 600 moths per cage per week will be made until the end season.

Releases of moths will be made early in the morning before the sun is up. RIDL moths will be supplied by the quarantine rearing facility at the CPHST laboratory in Phoenix. For each release, adult moths or pupae will be carried from the quarantine building to quarantine field cages in a plastic vial within a closed small ice chest. The chest and the vials with the moths will be opened once inside the cage with the doors sealed.

Sampling will take place every three weeks, sampling either flowers or fully developed green cotton bolls. On each sample date, a random sample of 50 flowers or bolls will be collected from each cage. Samples will be placed inside plastic boll emergence boxes (37 x 25 x 16.5 cm high) fitted with tight sealing lids, which will then be sealed with tape. These will be brought into the laboratory to allow any larvae to cut out from the bolls to pupate onto hexcel material in the bottom of the boll-box. Once pupation has occurred, the boll boxes will be opened inside the quarantine laboratory and the collected pupae will be examined with fluorescence microscopy to determine if they are RIDL or wild pink bollworm, and to score for mortality. Collected data

will be used to estimate RIDL mortality and infestation rates. A sample of non-fluorescent moths will be collected for PCR screening to test for possible dissociation of the fluorescent marker from the RIDL construct. All transgenic insects collected from cages will be destroyed by freezing at 20 ± 5° C for 48 hrs.

At the conclusion of the experiment, all plant material and insects from transgenic release cages will be destroyed by either heat treatment at 65 ± 5° C for 48 hours or by freezing at 20 ± 5° C for 48 hrs.

Experiment 2
The purpose of this experiment is to compare RIDL mortality rates obtained from the small scale experiments conducted on single cotton plants in quarantine field cages to laboratory tests conducted with artificial diet. The data will be used to estimate the percent mortality of RIDL progeny after a RIDL moth mates with a wild PBW moth. Because of the harsher and more variable conditions of the outdoor environment and the differences between cotton plants and artificial diet, RIDL progeny mortality rates may be higher when reared on a cotton plant than on artificial diet. Total mortality from all sources will need to be evaluated carefully before progress on implementing RIDL system insect can be fully developed.

This experiment will take place within one large quarantine field cage (3.6 x 7.3 x 1.8 m tall) placed over 4 rows of cotton as described above. Each experimental unit will consist of a mesh sleeve cage that fits on a 0.5 m branch of one cotton plant. At the start of an experiment a branch with several bolls will be covered with the sleeve. Release of five male moths of LA1124 (tTa effector gene only) or five male moths produced by a laboratory cross of LA476 (Nipper effector gene) by LA1124 will be placed in the cage with ten APHIS or wild collected female moths and allowed to mate and oviposit on the plants. This experiment will be repeated four times with 20 replicates per experiment.

This cross will result in the production of two genotypes, heterozygote RIDL moths and homozygous wild type moths. The progeny of these moths will be allowed to develop on the cotton plant for approximately 16 d (exact time determined by degree day model) and then all bolls in the cage will be collected and placed in boll boxes as described above.

Experiment 3
This experiment is similar to Experiment 1, but will be conducted on a much smaller scale allowing greater replication. Instead of multiple releases, only a single release of RIDL and 20 KR irradiated moths will be released against a native pink bollworm population. Lastly, release rates will vary in a geometric series to allow better estimation of the shape of the pest population under the two different control techniques.

This experiment will be conducted on a small scale in sleeve cages within a larger cage as described in experiment 2.

There are three treatments:

1) Release of RIDL pink bollworm adults with the LA1124 construct , as heterozygotes, homozygotes or doubly homozygous for two separate insertions of the LA1124 construct (e.g., LA1124A & LA1124B). Release rates will range in geometric progression from 2-64, there will be four replicates of each release rate.

2) Release of APHIS strain pink bollworm adults irradiated at the standard dose of 20 kilorads. Release rates will range in geometric progression from 2-64, there will be four replicates of each release rate.

3) There will be six replicates of a no release control.

For each experiment, 30 cotton branches at pin square or first bloom will be covered with a sleeve cage. Into each cage, 4 mating pairs of wild PBW and RIDL moths or 20KR APHIS at release rates of 2, 4, 8, 16, 32, and 64 moths will be made into each release cage. The progeny of these moths will be allowed to develop on the cotton plant for approximately 16 d (exact time determined by degree day model) and then all bolls in the cage will be collected and placed in boll boxes and brought into the laboratory as described above.

REFERENCES

Bartlett, A. C. 1978. Radiation-induced sterility in the pink bollworm. U. S. Dept. Agric., Sci. Edu. Admin., Agric. Rev. Manuals, ARM-W-1, 25 pp.

Bloem, S., Bloem,K. A., Carpenter, .J.,E., Calkins, C., O. 1999. Inherited sterility in codling moth (Lepidoptera: Tortricidae): effect of substerilizing doses of radiation on insect fecundity, fertility, and control. Annals-of-the-Entomological-Society-of-America. 1999; 92(2): 222-229 92: 222-229.

Carpenter, J. E., Hidrayani, N. Nelley,and B.G. Mullinix. 1997. Effect of substerilizing doses of radiation on sperm precedence in fall armyworm (Lepidoptera: Noctuidae). Journal of economic entomology. J. econ. entomol. Apr 90: 444-448.

Flint, H. M., R. T. Staten, and B. Wright. 1977. Irradiation of pink bollworm with substerilizing doses: Production of F1 progeny. Southwest. Entomol. 2: 16-19.

Flint, H. M., R. T. Staten, L. A. Bariola, and D. L. Palmer. 1973. Gamma-irradiated pink bollworms: Attractiveness, mating, and longevity of females. Environ. Entomol. 2: 97-100.

Flint, H. M., D. L. Palmer, L. A. Bariola, and B. Horn. 1974. Suppression of populations of native pink bollworm in field cages by the release of irradiated moths. J. Econ. Entomol. 67: 55-57.

Fryxell, K. J., Miller, T.A. 1995. Autocidal biological control: A general strategy for insect control based on genetic transformation with a highly conserved gene. Journal of economic entomology. 88: 1221-1232.

Graham, H. M., M. T. Ouye, R. D. Garcia, and H. H. de la Rosa. 1972. Dosages of gamma irradiation for full and inherited sterility in adult pink bollworms. J. Econ. Entomol. 65: 645-650.

Miller, E., D. Keaveny, R. T. Staten, A. Lowe, and J. Bomberg. 1994. Changes in pink bollworm (Lepidoptera: Gelechiidae) sooty mutant under animal and plant health inspection service mass-rearing methodology. J. Econ. Entomol. 87: 1659-1664.

Miller, T. A., M. Robertson, and S. Thibault. 1997. Conditional lethal genes to control cotton pests, pp. 1340-1341. National Cotton Council, Memphis, TN.
North, D. T. 1975. Inherited sterility in Lepidoptera. Annual Review of Entomology 20: 167-182.
Peng, G., Epton, J.E., Fu, G., Scaife, S., Hiscox, A., Condon, K.C, Condon, G.C, Morrison, I.N., Kelly, D.W., Dafa'alla, T., Coleman, P.G. & Alphey, L. 2005. A dominant lethal genetic system for autocidal control of the Mediterranean fruitfly. Nature Biotechnology.
Thomas, D. T., Donnelly, C.A., Wood, R.J. & Alphey, L.S. 2000. Insect Population control using a dominant repressible, lethal genetic system. Science 287: 2474-2476.

Enclosure F
A detailed description of the processes, procedures and safeguards which have been used or will be used in the country of origin and in the US to prevent contamination, release, and dissemination in the production of the donor organism; recipient organism; vector or vector agent; constituent of each regulated article which is a product and regulated article.

Movement of transgenic pink bollworm from the quarantine rearing facility to the field quarantine release cages will be in 40 dram molded plastic vials fitted with plastic snap caps or in 1/2 liter paper food serving cartons fitted with a tight sealing plastic lid. Insects will be loaded into these containers inside the quarantine facility then placed into an insulated Styrofoam or plastic ice chest with a tight sealing lid to make a double sealed system for transport of the transgenic insects.

The containers holding the insects will only be opened inside the sealed quarantine field cages over the cotton plots. The cages are placed over 4 rows of cotton and measure 3.6 x 7.3 x 1.8 m tall made of Lumite Saran 20 x 20 mesh. Cage covers are supported by a one inch diameter galvanized steel pipe frame. Each cage is fitted with a double door entry and each door is secured with both a heavy duty brass zipper and Velcro flap. The side of the cages are secured to the ground by burying 0.5 m of the side flap within a 0.5 m deep trench.

Only authorized personnel are allowed entry to a cage. Entry will be through the double door system, which will be operated to eliminate accidental escape by sealing the first entry door (or exit) before opening the next door. Before opening the second door to enter or leave the caged cotton, the door will be inspected to ensure that there are no moths resting on or near the cage door. Personnel will inspect and shake their clothing before leaving to the cotton area to make sure no moths hitchhike on clothing. The same procedures will be performed before leaving the cage through the door to the outside, inspecting the flap of the door, cage walls, roof and the space around the door before exiting.

The site will be surrounded by a 9-foot chain link fence, topped with razor wire with locked gates, limited entry authorization, and televised security monitoring. Besides the physical containment and security, biological containment will include the procedures outlined below to minimize escape or dispersal of EGFP-altered PBW.

The probability of escape from field cages will be negligible, barring a major weather catastrophe. Eight pheromone traps (sticky Delta™ traps) baited with 2 mg of gossyplure will be

strategically distributed around the cage area to capture any males that might escape from the cages. Huber et al. 1979 using 11 traps/hectare, reported mass trapping an effective tool for suppressing the adult male population of PBW in a cotton field. All cotton plants will be contained inside the cages. Once the experimental work is completed, all fruiting forms on the cotton plants will be removed and frozen for 48 h at -20 ± 5° C to eliminate all PBW life forms.

In place will be several PBW control strategies including pesticides and their application equipment that have been used or are currently being used to contain and/or control PBW populations. The implementation of these strategies around our field release cages and pheromone monitoring and control traps will make the risks negligible for the transgenic strain of PBW to transfer its genetic components to a field population of PBW or becoming established in the field.

Finally, RIDL insects are designed to die without tetracycline. Laboratory testing of over 12,000 individuals has shown that mortality of RIDL insects when reared without tetracycline express lethality as high as 100% with a range of 60-100%. These are rates of mortality seen in the favorable environment of the laboratory, it is expected that mortality rates will be much higher under field cage conditions. In the unlikely event of an escape from a cage, the probability of a fertile adult female moth finding a cotton plant, and any of the progeny surviving to reproduce, is very low.

REFERENCES

Huber, R. T., L. Moore, and M. P. Hoffman. 1979. Feasibility study of area-wide pheromone trapping of male pink bollworm moths in a cotton insect pest management program. J. Econ. Entomol. 72: 222-227.

ENCLOSURE G

A detailed description of the intended destination (including final and all intermediate destinations), uses, and/or distribution of the regulated article (e.g., greenhouses, laboratory, or growth chamber location; field trial location; pilot project location; production, propagation, and manufacture location; proposed sale and distribution location).

The LA1124 strains are currently reared in a quarantine laboratory in Phoenix AZ., which is operated under APHIS permit 98–244–02m.

Transgenic insects reared in quarantine facility for field release are released in quarantine field cages placed over four rows of cotton plants. The field is located within the city of Phoenix Arizona. Each plot will be covered with a 3.6 x 7.3 x 1.8 m tall screen cage (Lumite Saran 20 x 20 mesh) placed over a one inch diameter galvanized steel pipe frame. Each cage is fitted with a double door entry and each door is secured with both a heavy duty brass zipper and Velcro flap. The side of the cages are secured to the ground by burying 0.5 m of the side flap within a 0.5 m deep trench. Access to the test plots is controlled by a locked 9 foot high security fence. The test plot area is monitored by closed circuit security cameras.

At the conclusion of the test, all transgenic pink bollworm life forms will be destroyed.

ENCLOSURE H

A detailed description of the proposed procedures, processes, and safeguards which will be used to prevent escape and dissemination of the regulated article at each of the intended destinations.

Movement of transgenic pink bollworm from the quarantine rearing facility to the field quarantine release cages will be in 40 dram molded plastic vials fitted with plastic snap caps or in 1/2 liter paper food serving cartons fitted with a tight sealing plastic lid. Insects will be loaded into these containers inside the quarantine facility then placed into an insulated Styrofoam or plastic ice chest with a tight sealing lid to make a double sealed system for transport of the transgenic insects.

The containers holding the insects will only be opened inside the sealed quarantine field cages over the cotton plots. The cages are placed over 4 rows of cotton and measure 3.6 x 7.3 x 1.8 m tall made of Lumite Saran 20 x 20 mesh. Cage covers are supported by a one inch diameter galvanized steel pipe frame. Each cage is fitted with a double door entry and each door is secured with both a heavy duty brass zipper and Velcro flap. The side of the cages are secured to the ground by burying 0.5 m of the side flap within a 0.5 m deep trench.
Only authorized personnel are allowed entry to a cage. Entry will be through the double door system, which will be operated to eliminate accidental escape by sealing the first entry door (or exit) before opening the next door. Before opening the second door to enter or leave the caged cotton, the door will be inspected to ensure that there are no moths resting on or near the cage door. Personnel will inspect and shake their clothing before leaving to the cotton area to make sure no moths hitchhike on clothing. The same procedures will be performed before leaving the cage through the door to the outside, inspecting the flap of the door, cage walls, roof and the space around the door before exiting.

Cages will be monitored daily to ensure that the structure remains intact and all closures are sealed.

ENCLOSURE I

A detailed description of the proposed method of final disposition of the regulated article.

Transgenic PBW in the laboratory that are no longer needed will be disposed of by freezing at -20° ± 5° C for 48 hours. This will destroy any life stage of this insect. Transgenic PBW recaptured in the field cage trails will be disposed of by freezing at -20° ± 5° C for 48 hours. All plant fruiting forms in the release cages will be disposed of by freezing at –20° ± 5°C for 48 hours when the study is completed. This will destroy life stages that may infest the fruiting forms.

ENCLOSURE J
A description of the field trial location.

Transgenic insects reared in the Phoenix Pink Bollworm Genetic Rearing Facility selected for field release will be released in secure screened cages (3.6 × 7.3 × 1.8 m) placed over cotton plants. This location is in a highly secure security fenced area within an urban area in Phoenix, Arizona under the control of USDA authorities. No commercial cotton fields are within three miles of this field.

05-118-01R
add to permit

USDA

United States Department of Agriculture

Marketing and Regulatory Programs

Animal and Plant Health Inspection Service

4700 River Road
Riverdale, MD 20737

August 2, 2005

Dr. Gregory S. Simmons
USDA, APHIS, PPQ, CPHST, DSPMSL
3645 E. Wier Avenue
Phoenix, AZ 85040

Dear Dr. Simmons:

Subject: Biotechnology Permit Number 05-118-01r to Conduct a Planned Release of Genetically Engineered *Pectinophora gossypiella*

The above permit has been approved and you must adhere to the standard and supplemental conditions enclosed.

This permit should not be taken as any type of efficacy determination of the genetically engineered organisms.

Neil Hoffman for R.B.
Rebecca Bech
Associate Deputy Administrator
Biotechnology Regulatory Services

Enclosures:
Permit 05-118-01r
Supplemental Permit Conditions
Standard Permit Conditions
Map - Regional Biotechnologists

cc:
D. Madison, Arizona Department of Agriculture, Phoenix, AZ 85007
S. Wellstood, Compliance, BRS, Riverdale, MD 20737
File 05-118-01r

APHIS:BRS:JP:hll:8/1/2005:0511801r

APHIS - Protecting American Agriculture
An Equal Opportunity Employer

4/28

This application is authorized by the Federal Plant Pest Act (7 U.S.C. 150aa et seq. and the Plant Quarantine Act (7 U.S.C. 151 et seq.)). The information will be used to determine eligibility to receive all types of permits. No permit shall be issued until this application has been approved.

See reverse side for additional information

FORM APPROVED
OMB NO. -579-0085

U.S. DEPARTMENT OF AGRICULTURE
BIOTECHNOLOGY, BIOLOGICS, AND ENVIRONMENTAL PROTECTION

APPLICATION FOR PERMIT OR COURTESY PERMIT UNDER 7 CFR 340

(Genetically Engineered Organisms or Products)

INSTRUCTIONS: Complete this form and enclose the supporting materials listed on the reverse side. See page 3 for detailed instructions.

1. NAME AND ADDRESS OF APPLICANT

Gregory S. Simmons, Ph.D.
USDA-APHIS-PPQ-CPHST-DSPMSL
3645 E. Wier Ave
Phoenix, AZ 85040

2. PERMIT REQUESTED ("X" one)

- [ ] Limited - Interstate Movement
- [ ] Limited - Importation
- [x] Release into the Environment
- [ ] Courtesy Permit

3. THIS REQUEST IS ("X" one)

- [x] New
- [ ] Renewal
- [ ] Supplemental

4. TELEPHONE NUMBER

Area Code (602) 437-1295 05-118-01r

5. MEANS OF MOVEMENT

- [ ] Mail
- [ ] Common Carrier (FedEx)
- [ ] Baggage or Handcarried By whom

6. GIVE THE FOLLOWING (IF APPLICABLE) (IF MORE SPACE IS NEEDED, ATTACH ADDITIONAL SHEET)

	Scientific Name	Common Name	Trade Name	Other Designation
a. Donor Organism:	Aequorea victoria (jellyfish), Discosoma sp. (coral) Drosophila melanogaster (vinegar fly), Escherichia coli (bacterium), Herpes simplex (virus); OR, alternatively: Oryctolagus cuniculus (rabbit), Bombyx mori (silk moth), Aequorea victoria (jellyfish),Discosoma sp. (coral) Drosophila melanogaster (vinegar fly), Esherichia coli (bacterium), Simaian virus 40 (SV40)-(virus), Cytomegalovirus (CMV),(virus), Herpes simplex (virus), Synthetic.			
b. Recipient Organism:	Pectinophora gossypiella	(pink bollworm)	N/A	N/A
c. Vector or Vector Agent:	piggyBac	N/A	N/A	N/A
d. Regulated Organism or Product:	Pectinophora gossypiella	(pink bollworm)	N/A	N/A
e. If product, list names of constituents:	N/A	N/A	N/A	N/A

7. QUANTITY OF REGULATED ARTICLE TO BE INTRODUCED AND PROPOSED SCHECULE AND NUMBER OF INTRODUCTIONS

Multiple releases of 3,600 adults/wk for up to 20 wks for a total of 72,000 insects.

8. DATE (or inclusive dates of period) **OF IMPORTATION, INTERSTATE MOVEMENT, OR RELEASE**

For one year, intermittent year approval of permit

9. COUNTRY OR POINT OF ORIGIN OF THE REGULATED ARTICLE

Parent stock USDA-Phoenix,AZ transformed at the University of California or at Oxitech LTD, Oxford, UK.

10. PORT OF ARRIVAL, DESTINATION OF MOVEMENT, OR SPECIFIC LOCATION OF RELEASE

Contained Field Cage Trial in Phoenix, AZ

11. ANY BIOLOGICAL MATERIAL *(e.g., culture medium, or host material)* **ACCOMPANYING THE REGULATED ARTICLE DURING MOVEMENT**

N/A.

12. APPLICANTS FOR A COURTESY PERMIT - STATE WHY YOU BELIEVE THE ORGANISM OR PRODUCT DOES NOT COME WITHIN THE DEFINITION OF A REGULATED ARTICLE

N/A.

13. SEE REVERSE SIDE

I hereby certify that the information in the application and all attachments is complete and accurate to the best of my knowledge and belief.

False Statement: Falsification of any item on this application may result in a fine of not more than $10,000 or imprisonment for not more than 5 years or both. (18 U.S.C. 1001)

14. SIGNATURE OF RESPONSIBLE PERSON	15. PRINTED NAME AND TITLE	16. DATE
[signature]	Gregory S. Simmons, Ph.D.	04/27/2005

FOR APHIS USE ONLY

State Notification Letter Sent	State Review Received	Permit Issued
June 28, 2005	July 8, 2005	[X] Yes [ ] No

Date of Determination	Permit No.	No. of Permit Labels Issued	Supplemental Conditions Enclosed
August 2, 2005	05-118-01r	NA	[X] Yes [ ] No

Signature of BBEP Official	Date	Expiration Date
[signature] for RB<br>Rebecca Bech, Associate Deputy Admin., BRS	August 2, 2005	August 2, 2006

APHIS FORM 2000 (JUL 89) *Replaces PPQ Form 1001 which may be used*

Supplemental Conditions for 05-118-01r ând 05-115-01r

Studies with males and females will be conducted in 3.6 x 7.3 x 1.8 m screen field cages placed over cotton plants at the CPHST rearing facility.

The site will be surrounded by an 8 foot chain link fence, topped with razor wire with locked gates, video surveillance and limited entry authorization. It is at least 3 miles from the nearest cultivated cotton. The structure of the field cages is 2.54 cm galvanized pipe covered with a 16 x 16 mesh (256 openings per square inch) fiberglass screen with reinforced corners to prevent tears. The cages also have a 30.5 cm plastic skirt running along the bottom that is buried in the soil to prevent moth escapes.

An alternative site for confined studies is the same site used under permit 01-029-01R. This alternative site is surrounded by a 6 foot chain link fence.

Eight pheromone traps baited with 2 mg of Gossyplure™ will be strategically distributed around the cage area to capture any males that might escape from the cages.
All cotton plants exposed to transgenic moths will be contained in the cages.
Once the experimental work is done, all fruiting forms on the cotton will be removed and frozen at – 20°C for 24 hrs to eliminate all PBW life stages.

Adults, though capable of flight, will be contained in field cages. As an additional layer of containment, caged insects will have been irradiated with at least 10 kr of ^{60}Co gamma irradiation.

PBW control strategies should be in place and ready for deployment. They will include pesticides and application equipment that have been used or are currently being used to contain and/or control PBW populations.

Transgenic PBW that are no longer needed will be disposed of by freezing at -20° C for 24 hours. This will destroy any life stage of this insect. All plant fruiting forms in the release cages will be frozen at -20° C for 24 hours when the study is completed. This treatment will destroy PBW life stages that may infest the fruiting forms.

Reviewed|approved by JP 7|29|05

Standard Permit Conditions For the Introduction of a Regulated Article
(7 CFR 340.4 (f))

Permit Conditions: A person who is issued a permit and his/her employees or agents shall comply with the following conditions, and any supplemental conditions which shall be listed on the permit, as deemed by the Deputy Administrator to be necessary to prevent the dissemination and establishment of plant pests:

(1) The regulated article shall be maintained and disposed of (when necessary) in a manner so as to prevent the dissemination and establishment of plant pests.

(2) All packaging material, shipping containers, and any other material accompanying the regulated article shall be treated or disposed of in such a manner so as to prevent the dissemination and establishment of plant pests.

(3) The regulated article shall be kept separate from other organisms, except as specifically allowed in the permit.

(4) The regulated article shall be maintained only in areas and premises specified in the permit.

(5) An inspector shall be allowed access, during regular business hours, to the place where the regulated article is located and to any records relating to the introduction of a regulated article.

(6) The regulated article shall, when possible, be kept identified with a label showing the name of the regulated article, and the date of importation.

(7) The regulated article shall be subject to the application of measures determined by the Administrator to be necessary to prevent the accidental or unauthorized release of the regulated article.

(8) The regulated article shall be subject to the application of remedial measures (including disposal) determined by the administrator to be necessary to prevent the spread of plant pests.

(9) A person who has been issued a permit shall submit to APHIS a field test report within 6 months after the termination of the field test. A field test report shall include the APHIS reference number, methods of observation, resulting data, and analysis regarding all deleterious effects on plants, nontarget organisms, or the environment.

(10) APHIS shall be notified within the time periods and manner specified below, in the event of the following occurrences:

(i) Orally notified immediately upon discovery and notify in writing and within 24 hours in the event of any accidental or unauthroized release of the regulated article;

(ii) In writing as soon as possible but not later than within 5 working days if the regulated article or associated host organism is found to have characteristcs substantially different from those listed in the application for a permit or suffers any unusual occurrence (excessive mortality or morbidity, or unanticipated effect on non-target organisms).

(11) A permittee or his/her agent and any person who seeks to import a regulated article into the United States shall:

(i) Import or offer the regulated article for entry only at a port of entry which is designated by an asterisk in 7 CFR 319.37-14 (b);

(ii) Notify APHIS promptly upon arrival of any regulated article at a port of entry, or its arrival by such means as a manifest, customs entry document, commercial invoice, waybill, a broker's document, or a notice form provided for such purpose: and

(iii) Mark and identify the regulated article in accordance with 7 CFR 340.7.

Rev. 3/2003

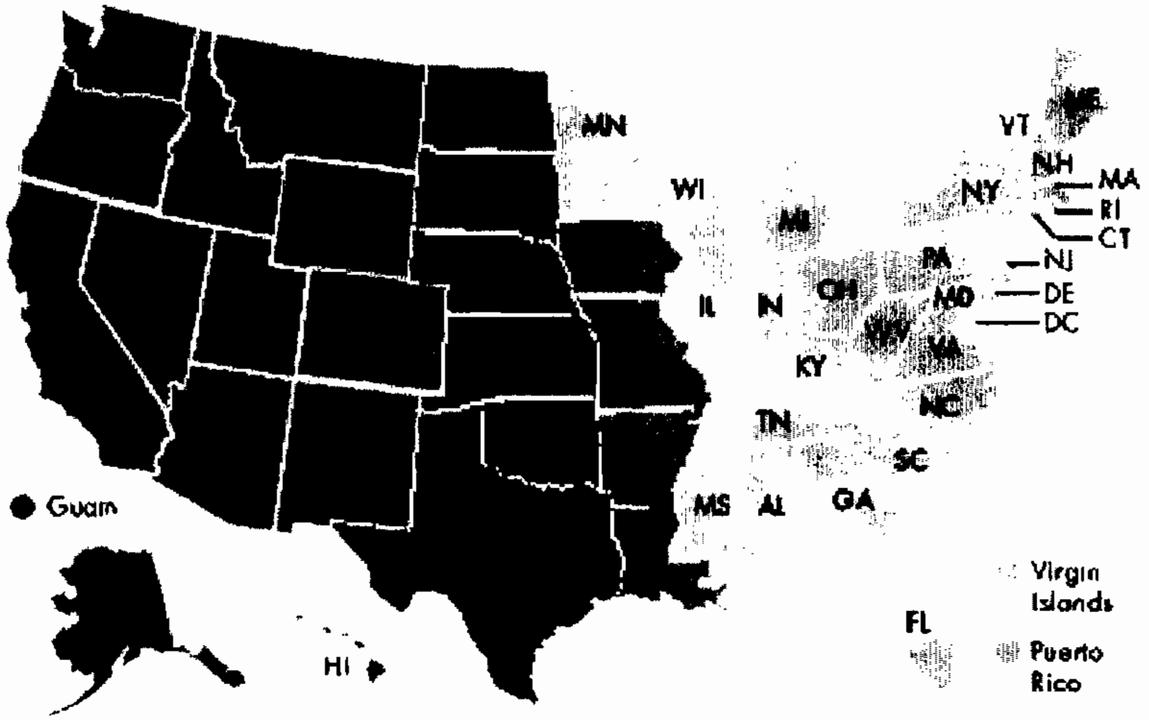

**Western Region**	**Eastern Region**
Ralph Stoaks<br>USDA, APHIS, BRS<br>2150 Centre Avenue<br>Building B, 3E10<br>Fort Collins, CO 80526<br>Phone: 970-494-7573<br>Biotechnology Fax: 970-494-7576<br>Fax: 970-494-7501<br>E-Mail: ralph.d.stoaks@aphis.usda.gov	Ashima Sengupta<br>USDA, APHIS, BRS<br>920 Main Campus Drive<br>Suite 200<br>Raleigh, NC 27606-5213<br>Phone: 919-855-7622<br>Fax: 919-855-7623<br>E-Mail: Ashima.Sengupta@aphis.usda.gov
The western region includes the states shaded in green plus: Alaska, American Samoa, Guam, Hawaii, Mariana Islands, Marshall Islands, Micronesia, and Palau.	*The eastern region includes the states shaded in yellow plus: Virgin Islands and Puerto Rico.*

	ENCLOSURES	ENCLOSED ("X")	IF PREVIOUSLY SUBMITTED, LIST DATE & PERMIT NO.
a.	Names, addresses, and telephone numbers of the persons who developed and/or supplied the regulated article.	X	
b.	A description of the anticipated or actual expression of the altered genetic material in the regulated article and how that expression differs from the expression in the nonmodified parental organism (e.g., morphological or structural characteristics, physiological activities and processes, number of copies of inserted genetic material and the physical state of this material inside the recipient organism (integrated or extrachromosomal), products and secretions, growth characteristics).	X	
c.	A detailed description of the molecular biology of the system (e.g., donor-recipient-vector) which is or will be used to produce the regulated article.	X	
d.	Country and locality where the donor organism, recipient organism, and vector or vector agent were collected, developed and produced.	X	
e.	A detailed description of the purpose of the introduction of the regulated article including a detailed description of the proposed experimental and/or production design.	X	
f	A detailed description of the processes, procedures, and safeguards which have been used or will be used in the country of origin and in the United States toprevent contamination, release, and dissemination in the production of the: donor organism; recipient organism; vector or vector agent; constituent of each reglated article which is a product; and regulated article.	X	
g	A detailed description of the intended destination (including final and all intermediate destinations), uses, and/or distribution of the regulated article (e.g., greenhouses, laboratory, or growth chamber location; field trial location, pilot project location; production, propagation, and manufacture location; proposed sale and distribution location).	X	
h.	A detailed description of the proposed procedures, processes, and safeguards which will be used to prevent escape and dissemination of the regulated article at each of the intended destinations.	X	
i.	A detailed description of the proposed method of final disposition of the regulated article.	X	

Public reporting burden for this collection of information is estimated to average 5 hours per response, including the time for reviewing instructions, searching existing data souces, gathering and maintaining the data needed, and completing and reviewing the collection of information. Send comments regarding this burden estimate or any other aspect of this collection of information, including suggestions for reducing this burden, to Department of Agriculture, Clearance Officer, OIRM, Room 404-W, Washington, D.C 20250; and to the Office of Information and Regulatory Affairs, Office of Management and Budget, Washington, D.C. 20503.

ENCLOSURE A
Names, addresses, and telephone numbers of the persons who developed and/or supplied the regulated article.

(b)(6)
Department of Entomology
University of California
Riverside, CA 92521
(b)(6)

(b)(6)
Oxitec Ltd
71 Milton Park
Abingdon
Oxford, OX14 4RX
UK
(b)(6)

(b)(6)
Decision Support & Pest Management Systems Laboratory
3645 E Wier Avenue
Phoenix, AZ 85040
(b)(6)

Dr. Gregory Simmons
Entomologist
Decision Support & Pest Management Systems Laboratory
3645 E Wier Avenue
Phoenix, AZ 85040
602–437-1295 x223

(b)(6)
Pink Bollworm Rearing Facility
3645 East Chipman
Phoenix, AZ 85040
(b)(6)

ENCLOSURE B
A description of the anticipated or actual expression of the altered genetic material in the regulated article and how that expression differs from the expression in the nonmodified parental organism.

The additional genetic material in the pink bollworm comprises several protein coding regions:

1. The marker.

This allows the expression of a fluorescent protein (e.g. GFP, DsRed) originally derived from the jellyfish *Aequoria victoria* or from a coral (e.g. *Discosoma* sp.). The transgenic pink bollworm with the marker gene fluoresces when excited by illumination of the appropriate wavelength. These fluorescent proteins, which have been used as markers in a wide range of vertebrate and invertebrate species, confer no known competitive advantage or disadvantage to the recipient, and no ecological or other consequences resulting from incorporation of these markers into the transgenic pink bollworm can be envisioned. The unmodified pink bollworm is not strongly fluorescent, expression of a fluorescent protein therefore allows the modified pink bollworm to be distinguished from unmodified.

2. Tetracycline-repressible transcriptional activator (tTA).

tTA protein binds to and activates expression from the tetracycline response element (tRE), which includes multiple copies of the specific DNA sequence to which tTA binds (tetO). tTA also binds tetracycline with high affinity; the tetracycline bound form of tTA does not bind DNA. tTA therefore acts as a tetracycline regulated switch - in the absence of tetracycline it will induce expression from tRE, whereas in the presence of tetracycline it will not. High level expression of tTA is thought to be deleterious to cells as it can repress their normal transcription; low level expression has no known effect other than activation of tRE. tTA is a synthetic fusion of the tetR protein from *Escherichia coli* with VP16 from herpes simplex virus. TetR provides the tetracycline-repressible sequence-specific DNA binding property, while VP16 is a eukaryotic transcriptional activator. tTA has been used in fungi, plants, mice, mammalian culture cells, the vinegar fly *Drosophila melanogaster*, and the Mediterranean fruit fly *Ceratitis capitata*, with no known adverse effects on the environment or on human health. Unmodified pink bollworm do not have a tTA gene or similar activity.

3. Effector gene.

The effector gene encodes an insect protein or RNA, or fragment thereof, expression of which is predicted to be deleterious to the insect. For example, in the case of the LA476 construct Nipper is the central domain of the Drosophila melanogaster Nipp1Dm gene. This binds to and inhibits the catalytic subunit of type 1 serine/threonine protein phosphatase (PP1c). PP1c is an essential enzyme, therefore high level expression of Nipper (or Nipp1Dm) kills the cell. In the modified pink bollworm, Nipper is under the transcriptional control of tRE, and so is expressed when tTA is present and tetracycline is not. Unmodified pink bollworm are thought to have a Nipp1-like gene, as this protein is present in other insects, the nematode *C. elegans* and mammals.

In the case of the LA1124 construct, tTA is placed under the transcriptional control of tRE, here tTA may itself act as an effector protein. Basal expression of tTA in the modified pink bollworm is predicted to have no visible effect on the modified pink bollworm under normal laboratory rearing conditions in the presence of tetracycline. High level expression of tTA in the absence of tetracycline is predicted to be deleterious to the moth, leading to a competitive disadvantage.

No piggyBac transposase activity nor any antibiotic or pesticide resistance is conferred to the transgenic pink bollworm by the introduced genetic material.

ENCLOSURE C
A detailed description of the molecular biology of the system that was used to produce the regulated article.

PiggyBac is a DNA (deoxyribonucleic acid) transposable element that, only when its ITRs (Inverted Terminal Repeats) are intact, is capable of integrating DNA flanked by element specific DNA into other DNA through mediation of a transposase encoded by an ORF (Open Reading Frame) within the element. In the construct used for transformation of the pink bollworm, the transposase gene of the piggyBac element was irreversibly destroyed by deletion of a section of the transposase gene. Transformation was effected by introducing with the transforming construct a helper plasmid that supplied transposase activity but was itself unable to transpose into other DNA. This transposition defective helper plasmid has an ORF (Open Reading Frame) encoding piggyBac transposase under the control of the *Drosophila melanogaster* hsp70 promoter. One of the inverted terminal repeats that flank the wild type piggyBac transposase in piggyBac has been removed in the helper plasmid so that the helper plasmid cannot, itself, integrate even though it encodes for active piggyBac transposase.
The potential for instability and unwanted mobilization of piggyBac derived transforming constructs must be addressed as follows. It could be argued that if there were endogenous, piggyBac-like elements in pink bollworm, they might provide a source of transposase that could mobilize transgenes flanked by piggyBac derived ITRs. Demonstration of elements homologous to piggyBac in the recipient organism, pink bollworm, might then suggest caution regarding stability of the transgene. However, the DNA mediated element, Hermes, has been used to successfully transform *Aedes aegypti* with little or no evidence of instability of the transgenes over at least 10 generations, even though there are in *Aedes aegypti* endogenous elements (presumably hAt-like as is Hermes) with close enough homology to Hermes so that these endogenous hAt and Hermes-like elements are detected even in higher stringency Southern blots with a Hermes probe. Similarly, piggyBac has been used successfully to transform the Oriental fruit fly, *Bactrocera dorsalis*, with no evidence of instability of the transgenes, even though closely related piggyBac-like elements were later found to be present in that species.

In the case of pink bollworm, low stringency Southern blot experiments on pink bollworm DNA with radio labeled DNA probes derived from piggyBac, which would be even more likely to detect elements with low homology to piggyBac than the higher stringency methods used in Jasinskiene, et al., 1998, were unable to detect any endogenous piggyBac-like elements. This suggests that there are no elements in pink bollworm that might reasonably be expected to mobilize a piggyBac derived transgene. In addition, excision and transposition assays were performed in pink bollworm embryos with piggyBac. This was primarily to determine if piggyBac could integrate into the pink bollworm genome. However, our results showed no transposition of piggyBac in the absence of exogenous piggyBac transposase in these transposition assays, strongly suggesting there were no unknown piggyBac-like elements in the pink bollworm genome capable of mobilizing non-autonomous piggyBac elements. We can thus be reasonably certain there would not be unexpected interactions between the components of the pink bollworm genome and the transforming construct that would result in instability of the transgenes. In any event, experiments to be performed in Phoenix after transfer of the transformed pink bollworm strains will further demonstrate the stability of the transgenes.

REFERENCES

Jasinskiene, N., Coates, C.J., Benedict, M.Q., Conrel, A.J., Rafferty, C.S., James, A.A., Collins, F.H.: Stable transformation of the Yellow Fever Mosquito, *Aedes aegypti*, with the hermes element from the housefly. Proceedings of the National Academy of Sciences of the United States of America, 1998 March 31, 95(7):3743-7.

ENCLOSURE D

Country and locality where the donor organism, recipient organism, and vector or agent were collected, developed and produced:

The United Kingdom, Oxford, the University of Oxford is where all final engineering of the transforming constructs were performed. The genes used from the donor organism and the piggyBac derived portions of the vectors used to build the transforming construct were cloned at this location.

The recipient organism—the pink bollworm, *Pectinophora gossypiella*—is an invasive insect whose origin is uncertain. It is not a native species of the Western Hemisphere though it is now endemic to the southwestern United States and Mexico, associated with commercial cotton production. Introduction of the pink bollworm into the United States appears to have been via infected cottonseed. The pink bollworm appeared in Hearn, TX in 1917, and within a decade it had spread across western Texas, New Mexico, and into Arizona by 1929. The colonies transformed at the University of California Riverside and at Oxford University, Oxford UK originated from the Pink Bollworm Rearing Facility in Phoenix, Arizona.

ENCLOSURE E

A detailed description of the purpose for the introduction of the regulated article including a detailed description of the proposed experimental design.

Pink bollworm (PBW) infestations cost U.S. cotton producers $47 million per year for direct losses and control measures (National Cotton Council, March 2005, unpublished brief). APHIS is involved in two PBW control projects using the release of sterile (SIT) PBW, *Suppression* in the Central Valley of California, and *Eradication* along with *B.t.* cotton, pheromones, and pesticides. The use of SIT will expand to 90,000 acres in 2005 when sterile releases are made in eradication program areas in Texas, New Mexico and the Juarez Valley in Mexico.

The SIT suppression program has been effective and kept the San Joaquin Valley free of PBW for 30 years at low cost. However, increased cotton production costs, worldwide competition and the increasing demands for the expanded PBW eradication program requires a more effective and lower cost program. A major limit on the efficacy of SIT as a control measure is the effect of sterilizing radiation on insect performance. Radiation has a great effect on the competitiveness and effectiveness of lepidoptera used in SIT programs and has been associated with decreased quality, competitiveness, and dispersal ability in many species (North 1975, Carpenter 1997, Bloem 1999) and lower dose radiation is associated with increased mating ability and superior sperm competitiveness (Carpenter 1997). In pink bollworm, the effects of radiation include reduced longevity, decreased sperm transfer by males, decreased sperm

receptivity, decreased female attractiveness and decreased control efficacy (Graham et al. 1972, Flint et al. 1973, Flint et al. 1974, Flint et al. 1977, Bartlett 1978, Miller et al. 1994).

Using genetic engineering to improve PBW control technology could achieve savings and greater program efficacy with the development of a PBW strain with an autocidal or conditionally lethal gene (Fryxell 1995, Miller et al. 1997), which would eliminate the need for irradiating released insects and greatly improve the performance and longevity of released moths.

The goal of this project is to develop a pink bollworm with a conditionally lethal gene using RIDL technology (**R**elease of **I**nsects with a **D**ominant **L**ethal gene, see Thomas 2000, Peng 2005) to make use in an innovative genetic control technique known as autocidal biological control. Progeny carrying a RIDL gene die when the antibiotic chlortetracycline (CTC) is absent. CTC is a normal ingredient in the PBW mass-rearing so a PBW strain with conditionally lethality controlled by CTC is a good choice for this rearing system. This is one of the most promising autocidal control systems in development.

The purpose of this experiment is to test the function of a conditionally lethal pink bollworm based on the RIDL technology in the more realistic conditions of the cotton plant within a quarantine field cage. Current RIDL strain pink bollworm express lethal phenotypes of 60-100% when reared without tetracycline on artificial diet in laboratory rearing conditions, this rate of mortality is expected to increase under the more challenging conditions of a real plant exposed to the stress of a changing environment with the extremes in temperature in the field that are unlike laboratory conditions.

The other goal of this experiment is to estimate the reduction of a wild pink bollworm population caused by release of a RIDL pink bollworm. This treatment will be compared to the reduction in wild pink bollworm caused by release of the standard APHIS strain moth irradiated at 20 kilorads.

Data from these experiments are needed for the next phase of development of a conditionally lethal pink bollworm moth. It is critical to determine if differences between the standard SIT release and RIDL release exist. If the RIDL insect is not as good as the SIT moth, is the difference within the range of improvement possible through outcrossing and strain improvement? If the two treatments have similar control efficacy, or if the RIDL insect is more efficacious, could RIDL release rates be reduced? Lastly, data from these experiments will provide key information about the differences between the two kinds of control technology (RIDL and irradiation) that will be needed for environmental analysis of the project that may be required under the National Environmental Policy Act (NEPA).

There are three experiments planned for testing the function of RIDL pink bollworm by releasing in quarantine field cages:

Experiment 1

This experiment is designed to simulate the season long release of RIDL and 20 KR irradiated moths against a native pink bollworm population within the range of densities that would be encountered within the eradication program.

There are three treatments:

1) Release of RIDL pink bollworm adults with the LA1124 construct, as heterozygotes, homozygotes or doubly homozygous for two separate insertions of the LA1124 construct (e.g., LA1124A & LA1124B).
2) Release of APHIS strain pink bollworm adults irradiated at the standard dose of 20 kilorads.
3) A no release control.

There are five replicates of treatments 1 and 2 and 4 replicates of treatment 3 arrayed in randomized incomplete blocks. Each replicate consists of a cotton plot of four rows of cotton grown in a plot measuring 7 m x 3.5 m wide. Cotton will be planted in mid to late April when soil temperatures are appropriate for germination. After the cotton has reached the 4-8 leaf stage, each plot will be covered with a 3.6 x 7.3 x 1.8 m tall screen cage (Lumite Saran 20 x 20 mesh) placed over a one inch diameter galvanized steel pipe frame. Each cage is fitted with a double door entry and each door is secured with both a heavy duty brass zipper and Velcro flap. The side of the cages are secured to the ground by burying 0.5 m of the side flap within a 0.5 m deep trench.

When the cotton reaches the pin square stage, 20 mating pairs of wild pink bollworm moths will be released into each cage to establish the test target population. Two days later, the first release of RIDL moths and 20KR APHIS moths will be released at the rate of 600 moths (1:1 sex ratio) per cage. No further releases will be made until just before the first generation (F1) of wild PBW moths emerges, this will be estimated by a degree day model and by sampling cotton flowers to determine developmental status. Upon emergence of the F1 generation releases of 600 moths per cage per week will be made until the end season.

Releases of moths will be made early in the morning before the sun is up. RIDL moths will be supplied by the quarantine rearing facility at the CPHST laboratory in Phoenix. For each release, adult moths or pupae will be carried from the quarantine building to quarantine field cages in a plastic vial within a closed small ice chest. The chest and the vials with the moths will be opened once inside the cage with the doors sealed.

Sampling will take place every three weeks, sampling either flowers or fully developed green cotton bolls. On each sample date, a random sample of 50 flowers or bolls will be collected from each cage. Samples will be placed inside plastic boll emergence boxes (37 x 25 x 16.5 cm high) fitted with tight sealing lids, which will then be sealed with tape. These will be brought into the laboratory to allow any larvae to cut out from the bolls to pupate onto hexcel material in the bottom of the boll-box. Once pupation has occurred, the boll boxes will be opened inside the quarantine laboratory and the collected pupae will be examined with fluorescence microscopy to determine if they are RIDL or wild pink bollworm, and to score for mortality. Collected data

will be used to estimate RIDL mortality and infestation rates. A sample of non-fluorescent moths will be collected for PCR screening to test for possible dissociation of the fluorescent marker from the RIDL construct. All transgenic insects collected from cages will be destroyed by freezing at 20 ± 5° C for 48 hrs.

At the conclusion of the experiment, all plant material and insects from transgenic release cages will be destroyed by either heat treatment at 65 ± 5° C for 48 hours or by freezing at 20 ± 5° C for 48 hrs.

Experiment 2

The purpose of this experiment is to compare RIDL mortality rates obtained from the small scale experiments conducted on single cotton plants in quarantine field cages to laboratory tests conducted with artificial diet. The data will be used to estimate the percent mortality of RIDL progeny after a RIDL moth mates with a wild PBW moth. Because of the harsher and more variable conditions of the outdoor environment and the differences between cotton plants and artificial diet, RIDL progeny mortality rates may be higher when reared on a cotton plant than on artificial diet. Total mortality from all sources will need to be evaluated carefully before progress on implementing RIDL system insect can be fully developed.

This experiment will take place within one large quarantine field cage (3.6 x 7.3 x 1.8 m tall) placed over 4 rows of cotton as described above. Each experimental unit will consist of a mesh sleeve cage that fits on a 0.5 m branch of one cotton plant. At the start of an experiment a branch with several bolls will be covered with the sleeve. Release of five male moths of LA1124 (tTa effector gene only) or five male moths produced by a laboratory cross of LA476 (Nipper effector gene) by LA1124 will be placed in the cage with ten APHIS or wild collected female moths and allowed to mate and oviposit on the plants. This experiment will be repeated four times with 20 replicates per experiment.

This cross will result in the production of two genotypes, heterozygote RIDL moths and homozygous wild type moths. The progeny of these moths will be allowed to develop on the cotton plant for approximately 16 d (exact time determined by degree day model) and then all bolls in the cage will be collected and placed in boll boxes as described above.

Experiment 3

This experiment is similar to Experiment 1, but will be conducted on a much smaller scale allowing greater replication. Instead of multiple releases, only a single release of RIDL and 20 KR irradiated moths will be released against a native pink bollworm population. Lastly, release rates will vary in a geometric series to allow better estimation of the shape of the pest population under the two different control techniques.

This experiment will be conducted on a small scale in sleeve cages within a larger cage as described in experiment 2.

There are three treatments:

1) Release of RIDL pink bollworm adults with the LA1124 construct , as heterozygotes, homozygotes or doubly homozygous for two separate insertions of the LA1124 construct (e.g., LA1124A & LA1124B). Release rates will range in geometric progression from 2-64, there will be four replicates of each release rate.

2) Release of APHIS strain pink bollworm adults irradiated at the standard dose of 20 kilorads. Release rates will range in geometric progression from 2-64, there will be four replicates of each release rate.

3) There will be six replicates of a no release control.

For each experiment, 30 cotton branches at pin square or first bloom will be covered with a sleeve cage. Into each cage, 4 mating pairs of wild PBW and RIDL moths or 20KR APHIS at release rates of 2, 4, 8, 16, 32, and 64 moths will be made into each release cage. The progeny of these moths will be allowed to develop on the cotton plant for approximately 16 d (exact time determined by degree day model) and then all bolls in the cage will be collected and placed in boll boxes and brought into the laboratory as described above.

REFERENCES

Bartlett, A. C. 1978. Radiation-induced sterility in the pink bollworm. U. S. Dept. Agric., Sci. Edu. Admin., Agric. Rev. Manuals, ARM-W-1, 25 pp.

Bloem, S., Bloem,K. A., Carpenter, .J.,E., Calkins, C., O. 1999. Inherited sterility in codling moth (Lepidoptera: Tortricidae): effect of substerilizing doses of radiation on insect fecundity, fertility, and control. Annals-of-the-Entomological-Society-of-America. 1999; 92(2): 222-229 92: 222-229.

Carpenter, J. E., Hidrayani, N. Nelley,and B.G. Mullinix. 1997. Effect of substerilizing doses of radiation on sperm precedence in fall armyworm (Lepidoptera: Noctuidae). Journal of economic entomology. J. econ. entomol. Apr 90: 444-448.

Flint, H. M., R. T. Staten, and B. Wright. 1977. Irradiation of pink bollworm with substerilizing doses: Production of F1 progeny. Southwest. Entomol. 2: 16-19.

Flint, H. M., R. T. Staten, L. A. Bariola, and D. L. Palmer. 1973. Gamma-irradiated pink bollworms: Attractiveness, mating, and longevity of females. Environ. Entomol. 2: 97-100.

Flint, H. M., D. L. Palmer, L. A. Bariola, and B. Horn. 1974. Suppression of populations of native pink bollworm in field cages by the release of irradiated moths. J. Econ. Entomol. 67: 55-57.

Fryxell, K. J., Miller, T.A. 1995. Autocidal biological control: A general strategy for insect control based on genetic transformation with a highly conserved gene. Journal of economic entomology. 88: 1221-1232.

Graham, H. M., M. T. Ouye, R. D. Garcia, and H. H. de la Rosa. 1972. Dosages of gamma irradiation for full and inherited sterility in adult pink bollworms. J. Econ. Entomol. 65: 645-650.

Miller, E., D. Keaveny, R. T. Staten, A. Lowe, and J. Bomberg. 1994. Changes in pink bollworm (Lepidoptera: Gelechiidae) sooty mutant under animal and plant health inspection service mass-rearing methodology. J. Econ. Entomol. 87: 1659-1664.

Miller, T. A., M. Robertson, and S. Thibault. 1997. Conditional lethal genes to control cotton pests, pp. 1340-1341. National Cotton Council, Memphis, TN.
North, D. T. 1975. Inherited sterility in Lepidoptera. Annual Review of Entomology 20: 167-182.
Peng, G., Epton, J.E., Fu, G., Scaife, S., Hiscox, A., Condon, K.C, Condon, G.C, Morrison, I.N., Kelly, D.W., Dafa'alla, T., Coleman, P.G. & Alphey, L. 2005. A dominant lethal genetic system for autocidal control of the Mediterranean fruitfly. Nature Biotechnology.
Thomas, D. T., Donnelly, C.A., Wood, R.J. & Alphey, L.S. 2000. Insect Population control using a dominant repressible, lethal genetic system. Science 287: 2474-2476.

Enclosure F
A detailed description of the processes, procedures and safeguards which have been used or will be used in the country of origin and in the US to prevent contamination, release, and dissemination in the production of the donor organism; recipient organism; vector or vector agent; constituent of each regulated article which is a product and regulated article.

Movement of transgenic pink bollworm from the quarantine rearing facility to the field quarantine release cages will be in 40 dram molded plastic vials fitted with plastic snap caps or in 1/2 liter paper food serving cartons fitted with a tight sealing plastic lid. Insects will be loaded into these containers inside the quarantine facility then placed into an insulated Styrofoam or plastic ice chest with a tight sealing lid to make a double sealed system for transport of the transgenic insects.

The containers holding the insects will only be opened inside the sealed quarantine field cages over the cotton plots. The cages are placed over 4 rows of cotton and measure 3.6 x 7.3 x 1.8 m tall made of Lumite Saran 20 x 20 mesh. Cage covers are supported by a one inch diameter galvanized steel pipe frame. Each cage is fitted with a double door entry and each door is secured with both a heavy duty brass zipper and Velcro flap. The side of the cages are secured to the ground by burying 0.5 m of the side flap within a 0.5 m deep trench.

Only authorized personnel are allowed entry to a cage. Entry will be through the double door system, which will be operated to eliminate accidental escape by sealing the first entry door (or exit) before opening the next door. Before opening the second door to enter or leave the caged cotton, the door will be inspected to ensure that there are no moths resting on or near the cage door. Personnel will inspect and shake their clothing before leaving to the cotton area to make sure no moths hitchhike on clothing. The same procedures will be performed before leaving the cage through the door to the outside, inspecting the flap of the door, cage walls, roof and the space around the door before exiting.

The site will be surrounded by a 9-foot chain link fence, topped with razor wire with locked gates, limited entry authorization, and televised security monitoring. Besides the physical containment and security, biological containment will include the procedures outlined below to minimize escape or dispersal of EGFP-altered PBW.

The probability of escape from field cages will be negligible, barring a major weather catastrophe. Eight pheromone traps (sticky Delta™ traps) baited with 2 mg of gossyplure will be

strategically distributed around the cage area to capture any males that might escape from the cages. Huber et al. 1979 using 11 traps/hectare, reported mass trapping an effective tool for suppressing the adult male population of PBW in a cotton field. All cotton plants will be contained inside the cages. Once the experimental work is completed, all fruiting forms on the cotton plants will be removed and frozen for 48 h at -20 ± 5° C to eliminate all PBW life forms.

In place will be several PBW control strategies including pesticides and their application equipment that have been used or are currently being used to contain and/or control PBW populations. The implementation of these strategies around our field release cages and pheromone monitoring and control traps will make the risks negligible for the transgenic strain of PBW to transfer its genetic components to a field population of PBW or becoming established in the field.

Finally, RIDL insects are designed to die without tetracycline. Laboratory testing of over 12,000 individuals has shown that mortality of RIDL insects when reared without tetracycline express lethality as high as 100% with a range of 60-100%. These are rates of mortality seen in the favorable environment of the laboratory, it is expected that mortality rates will be much higher under field cage conditions. In the unlikely event of an escape from a cage, the probability of a fertile adult female moth finding a cotton plant, and any of the progeny surviving to reproduce, is very low.

REFERENCES

Huber, R. T., L. Moore, and M. P. Hoffman. 1979. Feasibility study of area-wide pheromone trapping of male pink bollworm moths in a cotton insect pest management program. J. Econ. Entomol. 72: 222-227.

ENCLOSURE G

A detailed description of the intended destination (including final and all intermediate destinations), uses, and/or distribution of the regulated article (e.g., greenhouses, laboratory, or growth chamber location; field trial location; pilot project location; production, propagation, and manufacture location; proposed sale and distribution location).

The LA1124 strains are currently reared in a quarantine laboratory in Phoenix AZ., which is operated under APHIS permit 98–244–02m.

Transgenic insects reared in quarantine facility for field release are released in quarantine field cages placed over four rows of cotton plants. The field is located within the city of Phoenix Arizona. Each plot will be covered with a 3.6 x 7.3 x 1.8 m tall screen cage (Lumite Saran 20 x 20 mesh) placed over a one inch diameter galvanized steel pipe frame. Each cage is fitted with a double door entry and each door is secured with both a heavy duty brass zipper and Velcro flap. The side of the cages are secured to the ground by burying 0.5 m of the side flap within a 0.5 m deep trench. Access to the test plots is controlled by a locked 9 foot high security fence. The test plot area is monitored by closed circuit security cameras.

At the conclusion of the test, all transgenic pink bollworm life forms will be destroyed.

ENCLOSURE H
A detailed description of the proposed procedures, processes, and safeguards which will be used to prevent escape and dissemination of the regulated article at each of the intended destinations.

Movement of transgenic pink bollworm from the quarantine rearing facility to the field quarantine release cages will be in 40 dram molded plastic vials fitted with plastic snap caps or in 1/2 liter paper food serving cartons fitted with a tight sealing plastic lid. Insects will be loaded into these containers inside the quarantine facility then placed into an insulated Styrofoam or plastic ice chest with a tight sealing lid to make a double sealed system for transport of the transgenic insects.

The containers holding the insects will only be opened inside the sealed quarantine field cages over the cotton plots. The cages are placed over 4 rows of cotton and measure 3.6 x 7.3 x 1.8 m tall made of Lumite Saran 20 x 20 mesh. Cage covers are supported by a one inch diameter galvanized steel pipe frame. Each cage is fitted with a double door entry and each door is secured with both a heavy duty brass zipper and Velcro flap. The side of the cages are secured to the ground by burying 0.5 m of the side flap within a 0.5 m deep trench.
Only authorized personnel are allowed entry to a cage. Entry will be through the double door system, which will be operated to eliminate accidental escape by sealing the first entry door (or exit) before opening the next door. Before opening the second door to enter or leave the caged cotton, the door will be inspected to ensure that there are no moths resting on or near the cage door. Personnel will inspect and shake their clothing before leaving to the cotton area to make sure no moths hitchhike on clothing. The same procedures will be performed before leaving the cage through the door to the outside, inspecting the flap of the door, cage walls, roof and the space around the door before exiting.

Cages will be monitored daily to ensure that the structure remains intact and all closures are sealed.

ENCLOSURE I
A detailed description of the proposed method of final disposition of the regulated article.

Transgenic PBW in the laboratory that are no longer needed will be disposed of by freezing at -20° ± 5° C for 48 hours. This will destroy any life stage of this insect. Transgenic PBW recaptured in the field cage trails will be disposed of by freezing at -20° ± 5° C for 48 hours. All plant fruiting forms in the release cages will be disposed of by freezing at –20° ± 5°C for 48 hours when the study is completed. This will destroy life stages that may infest the fruiting forms.

ENCLOSURE J

A description of the field trial location.

Transgenic insects reared in the Phoenix Pink Bollworm Genetic Rearing Facility selected for field release will be released in secure screened cages (3.6 × 7.3 × 1.8 m) placed over cotton plants. This location is in a highly secure security fenced area within an urban area in Phoenix, Arizona under the control of USDA authorities. No commercial cotton fields are within three miles of this field.

05-118-01R

add to permit

Supplemental Conditions for 05 05-118-01r and 05-115-01r

Studies with males and females will be conducted in 3.6 x 7.3 x 1.8 m screen field cages placed over cotton plants at the CPHST rearing facility.

The site will be surrounded by an 8 foot chain link fence, topped with razor wire with locked gates, video surveillance and limited entry authorization. It is at least 3 miles from the nearest cultivated cotton. The structure of the field cages is 2.54 cm galvanized pipe covered with a 16 x 16 mesh (256 openings per square inch) fiberglass screen with reinforced corners to prevent tears. The cages also have a 30.5 cm plastic skirt running along the bottom that is buried in the soil to prevent moth escapes.

An alternative site for confined studies is the same site used under permit 01-029-01R. This alternative site is surrounded by a 6 foot chain link fence.

Eight pheromone traps baited with 2 mg of Gossyplure™ will be strategically distributed around the cage area to capture any males that might escape from the cages.
All cotton plants exposed to transgenic moths will be contained in the cages.
Once the experimental work is done, all fruiting forms on the cotton will be removed and frozen at – 20°C for 24 hrs to eliminate all PBW life stages.

Adults, though capable of flight, will be contained in field cages. As an additional layer of containment, caged insects will have been irradiated with at least 10 kr of ^{60}Co gamma irradiation.

PBW control strategies should be in place and ready for deployment. They will include pesticides and application equipment that have been used or are currently being used to contain and/or control PBW populations.

Transgenic PBW that are no longer needed will be disposed of by freezing at -20° C for 24 hours. This will destroy any life stage of this insect. All plant fruiting forms in the release cages will be frozen at -20° C for 24 hours when the study is completed. This treatment will destroy PBW life stages that may infest the fruiting forms.

Reviewed|approved by JP 7|29|05

APPLICATION NO.: 05-118-01R
DATE: JUNE 28, 2005

APHIS'S RESPONSE TO APHIS' INITIAL REVIEW OF AN APPLICATION FOR THE INTRODUCTION OF A REGULATED ARTICLE UNDER 7 CFR 340

☐ State concurs with APHIS' initial review.

☐ State concurs with APHIS' initial review and offers the following comments (use additional sheets if necessary):

☐ State *does not concur* with APHIS' initial review and offers the following reasons for nonconcurrence (use additional sheets if necessary):

Name of State Official:

Title:

Agency or Department: ARIZONA

City, State, Zip Code:

Date:

PLEASE RETURN THIS FORM WITHIN 30 DAYS OF THE DATE LISTED ABOVE TO:

Linda Lightle
USDA, APHIS, PPQ, PRA
Biotechnology Evaluation
4700 River Road, Unit 147
Riverdale, MD 20737
301/734-5787/8231/Fax: 301/734-8910

June 28, 2005

Mr. David Madison
Arizona Department of Agriculture
1688 W. Adams St.
Phoenix, AZ 85007

Dear Mr. Madison:

This letter accompanies permit application No. 05-118-01r submitted by Dr. Gregory S. Simmons, USDA, AHIPS, PPQ, Center for Plant Health Science and Technology, DSPMSL, Phoenix Plant Protection Center, 3645 East Wiser Avenue, Phoenix, Arizona, in collaboration with [redacted] (b)(6) of the Center for the purpose of doing cage-contained studies of transgenic pink bollworms (PBW) on cotton. This work is similar to the work done under USDA APHIS permits No. 03-104-01r and 01-029-01r. An Environmental Assessment (EA) under the national Environmental Policy Act was conducted and a Finding of No Significant Impact (FONSI) was reached regarding to make the decision to issue APHIS permits No. 03-104-01r and 01-029-01r. Because an EA has already been conducted for these preceding permit applications, and the permit research proposed in application No. 05-118-01r submitted by Dr. Simmons is similar and equivalently contained, criteria for an EA are no longer met under 7 CFR 372.5 (d) (4) “When a confined field release of genetically engineered organisms or products involves new species or organisms or novel modifications that raise new issues”. It is therefore not required to conduct another EA for this current permit application. There are no claims of confidential business information in any of the documentation.

PBW is one of the most destructive pests of cotton in the world. It was first found in the United States in 1917 and has become a pest in Texas, New Mexico, Arizona and California. Costs relating to prevention, control and yield losses have been estimated by the National Cotton Council to exceed $24 million annually. The San Joaquin Valley of California remains the last cotton growing area in the Southwest that is not generally infested with PBW. Prevention of its establishment in this valley is attributed primarily to the ongoing Sterile-Insect Technique (SIT) program established jointly in 1968 by APHIS, California Department of Food and Agriculture, and the California cotton growers.

An objective of the proposed research of this permit application is to develop a strain of PBW expressing coelenterate-derived Fluorescent Protein (EGFP or DsRed) marker genes and an autocidal effector gene construct (from Oxitec, Oxford, UK). The latter transgene will fatally disrupt the development of insects carrying this gene (particularly the progeny of mating between transgenic insects and wild type insects) when these insects are not supplied with a specific small molecule repressor (a tetracycline derivative). These cage studies are designed to test the function and effectiveness of autocidal transgenes and to determine the effectiveness of these autocidal insects in reducing experimental populations in a fully-contained experiment. Also, this experiment will compare the mating biology of these fluorescent-protein-producing, autocidal insects

to that of wild type colony insects and irradiated wild type colony insects in a fully contained experimental environment. Implementation of genetically marked autocidal insects (with fluorescent proteins) into a PBW mass-rearing SIT program could provide a more effective alternative to irradiation or could reduce the necessary radiation dose to implement SIT, thereby increasing the effectiveness of an already demonstrably successful control program for the exotic pest, PBW. Finally, a genetically marked insect will be a useful monitoring tool for field managers to determine the distribution of treated PBW and to gauge the most efficient means of doing so.

Initial studies with males and females will be conducted in 3.6 x 7.3 x 1.8 m screen field cages placed over cotton plants at the CPHST rearing facility. The site is surrounded by an 8 foot chain link fence, topped with razor wire with locked gates, video surveillance and limited entry authorization. It is at least 3 miles from the nearest cultivated cotton. Adults, though capable of flight, will be contained in field cages. The structure of the field cages is 2.54 cm galvanized pipe covered with Lumite™ Saran™ 20 x 20 mesh fiberglass screen with reinforced corners to prevent tears. This mesh is tighter than mesh used in previously contained studies and as such is even less likely to allow escapes of contained moths than the materials used in USDA APHIS permits No. 03-104-01R and 01-029-01R. Though the adult moths cannot burrow, the cages also have a 30.5 cm plastic skirt running along the bottom that is buried in the soil to prevent moth escapes through soil cracks. An alternative site for confined studies is the same site used under permit 01-029-01R. This alternative site is surrounded by a 6 foot chain link fence.

Escape from such field cages is highly unlikely barring a major weather catastrophe, which itself is likely to destroy the contained insects. Eight pheromone traps baited with 2 mg of Gossyplure™ will be strategically distributed around the cage area to capture any males that might escape from the cages. All cotton plants in the area will be contained in the cages. Once the experimental work is done, all fruiting forms on the cotton will be removed and frozen at – 20°C for 24 hrs to eliminate all PBW life stages.

PBW control strategies will be in place and ready for deployment. They include pesticides and application equipment that have been used or are currently being used to contain and/or control PBW populations. The implementation of these strategies around the field cages and pheromone monitoring and control traps will make the risks negligible for the transgenic strain of PBW to transfer its genetic components to a field population of PBW or of this strain becoming established in the field. Risk is further minimized by research which has established that laboratory rearing of over 74 generations of the transgenic PBW strain give no indication that a transgenic EGFP strain has any competitive advantage over the strains currently maintained in the pink bollworm rearing facility.

Transgenic PBW that are no longer needed will be disposed of by freezing at -20° C for 48 hours. This will destroy any life stage of this insect. All plant fruiting forms in the release cages

will be frozen at -20° C for 24 hours when the study is completed. This treatment will destroy PBW life stages that may infest the fruiting forms.

Previously conducted experiments demonstrated there are no transposases in the PBW genome that mobilize *piggyBac* transposon. This fact is addressed in the EA that has already been conducted resulting in the decision to issue APHIS permits No. 03-104-01R and 01-029-01R. Since there is no identifiable direct effect of this field test on any wild plant or animal species, there is no apparent risk to any threatened or endangered species. The proposed experiments are not expected to cause any adverse environmental effects due to their physical and biological containment. PWB also has no sexually compatible relatives in the United States with which it could reproduce or hybridize.

The application was submitted pursuant to regulations found in 7 CFR Part 340 which regulate the importation, interstate movement, or release into the environment of geneticat1y engineered plant pests. The regulations require that a person obtain a permit from APHIS prior to introducing a regulated article. This letter serves to give notice to and affords the State of Arizona the opportunity to indicate concurrence or non-concurrence with APHIS' assessment that contained field testing of these genetically modified insects does not pose a plant risk. You may also provide any conditions that may be mandated by your State. Please review the enclosed documents and return acknowledgement, associated comments, or reasons for non-concurrence (if applicable) to APHIS within 30 days from the date of this letter or preferably sooner (please use the enclosed form; use additional sheets for response, if needed).

Please refer to permit No. 05-118-01r in your correspondence regarding this application. If you have any questions about this application, please contact me at (301) 734-5720, facsimile (301) 734-8669, or e-mail: john.j.peloquin@aphis.usda.gov.

APHIS hopes to maintain its excellent working relationship with your State and encourages your participation and comments prior to our final decision regarding this permit application.

Sincerely,

John J. Peloquin, Ph.D.
Supervisory Biotechnologist/Entomologist
Animals Branch Chief
Biotechnology Regulatory Services

Enclosures:
Permit Application No. 05-118-01r
State Response Form

Cc:
S. Wellstood, Compliance Branch, Rivderdale, MD 20737
File 05-118-01r

APHIS:BRS:JP:hll:x8231:6/27/2005:0511801r

USDA

United States Department of Agriculture

Marketing and Regulatory Programs

Animal and Plant Health Inspection Service

4700 River Road
Riverdale, MD 20737

June 28, 2005

Mr. David Madison
Arizona Department of Agriculture
1688 W. Adams St.
Phoenix, AZ 85007

Dear Mr. Madison:

This letter accompanies permit application No. 05-118-01r submitted by Dr. Gregory S. Simmons, USDA, AHIPS, PPQ, Center for Plant Health Science and Technology, DSPMSL, Phoenix Plant Protection Center, 3645 East Wiser Avenue, Phoenix, Arizona, in collaboration with (b)(6) of the Center for the purpose of doing cage-contained studies of transgenic pink bollworms (PBW) on cotton. This work is similar to the work done under USDA APHIS permits No. 03-104-01r and 01-029-01r. An Environmental Assessment (EA) under the national Environmental Policy Act was conducted and a Finding of No Significant Impact (FONSI) was reached regarding to make the decision to issue APHIS permits No. 03-104-01r and 01-029-01r. Because an EA has already been conducted for these preceding permit applications, and the permit research proposed in application No. 05-118-01r submitted by Dr. Simmons is similar and equivalently contained, criteria for an EA are no longer met under 7 CFR 372.5 (d) (4) "When a confined field release of genetically engineered organisms or products involves new species or organisms or novel modifications that raise new issues". It is therefore not required to conduct another EA for this current permit application. There are no claims of confidential business information in any of the documentation.

PBW is one of the most destructive pests of cotton in the world. It was first found in the United States in 1917 and has become a pest in Texas, New Mexico, Arizona and California. Costs relating to prevention, control and yield losses have been estimated by the National Cotton Council to exceed $24 million annually. The San Joaquin Valley of California remains the last cotton growing area in the Southwest that is not generally infested with PBW. Prevention of its establishment in this valley is attributed primarily to the ongoing Sterile-Insect Technique (SIT) program established jointly in 1968 by APHIS, California Department of Food and Agriculture, and the California cotton growers.

An objective of the proposed research of this permit application is to develop a strain of PBW expressing coelenterate-derived Fluorescent Protein (EGFP or DsRed) marker genes and an autocidal effector gene construct (from Oxitec, Oxford, UK). The latter transgene will fatally disrupt the development of insects carrying this gene (particularly the progeny of mating between transgenic insects and wild type insects) when these insects are not supplied with a specific small molecule repressor (a tetracycline derivative). These cage studies are designed to test the function and effectiveness of autocidal transgenes and to determine the effectiveness of these autocidal insects in reducing experimental populations in a fully-contained experiment. Also, this experiment will compare the mating biology of these fluorescent-protein-producing, autocidal insects

APHIS - Protecting American Agriculture
An Equal Opportunity Employer

to that of wild type colony insects and irradiated wild type colony insects in a fully contained experimental environment. Implementation of genetically marked autocidal insects (with fluorescent proteins) into a PBW mass-rearing SIT program could provide a more effective alternative to irradiation or could reduce the necessary radiation dose to implement SIT, thereby increasing the effectiveness of an already demonstrably successful control program for the exotic pest, PBW. Finally, a genetically marked insect will be a useful monitoring tool for field managers to determine the distribution of treated PBW and to gauge the most efficient means of doing so.

Initial studies with males and females will be conducted in 3.6 x 7.3 x 1.8 m screen field cages placed over cotton plants at the CPHST rearing facility. The site is surrounded by an 8 foot chain link fence, topped with razor wire with locked gates, video surveillance and limited entry authorization. It is at least 3 miles from the nearest cultivated cotton. Adults, though capable of flight, will be contained in field cages. The structure of the field cages is 2.54 cm galvanized pipe covered with Lumite™ Saran™ 20 x 20 mesh fiberglass screen with reinforced corners to prevent tears. This mesh is tighter than mesh used in previously contained studies and as such is even less likely to allow escapes of contained moths than the materials used in USDA APHIS permits No. 03-104-01R and 01-029-01R. Though the adult moths cannot burrow, the cages also have a 30.5 cm plastic skirt running along the bottom that is buried in the soil to prevent moth escapes through soil cracks. An alternative site for confined studies is the same site used under permit 01-029-01R. This alternative site is surrounded by a 6 foot chain link fence.

Escape from such field cages is highly unlikely barring a major weather catastrophe, which itself is likely to destroy the contained insects. Eight pheromone traps baited with 2 mg of Gossyplure™ will be strategically distributed around the cage area to capture any males that might escape from the cages. All cotton plants in the area will be contained in the cages. Once the experimental work is done, all fruiting forms on the cotton will be removed and frozen at – 20°C for 24 hrs to eliminate all PBW life stages.

PBW control strategies will be in place and ready for deployment. They include pesticides and application equipment that have been used or are currently being used to contain and/or control PBW populations. The implementation of these strategies around the field cages and pheromone monitoring and control traps will make the risks negligible for the transgenic strain of PBW to transfer its genetic components to a field population of PBW or of this strain becoming established in the field. Risk is further minimized by research which has established that laboratory rearing of over 74 generations of the transgenic PBW strain give no indication that a transgenic EGFP strain has any competitive advantage over the strains currently maintained in the pink bollworm rearing facility.

Transgenic PBW that are no longer needed will be disposed of by freezing at -20° C for 48 hours. This will destroy any life stage of this insect. All plant fruiting forms in the release cages

will be frozen at -20° C for 24 hours when the study is completed. This treatment will destroy PBW life stages that may infest the fruiting forms.

Previously conducted experiments demonstrated there are no transposases in the PBW genome that mobilize *piggyBac* transposon. This fact is addressed in the EA that has already been conducted resulting in the decision to issue APHIS permits No. 03-104-01R and 01-029-01R. Since there is no identifiable direct effect of this field test on any wild plant or animal species, there is no apparent risk to any threatened or endangered species. The proposed experiments are not expected to cause any adverse environmental effects due to their physical and biological containment. PWB also has no sexually compatible relatives in the United States with which it could reproduce or hybridize.

The application was submitted pursuant to regulations found in 7 CFR Part 340 which regulate the importation, interstate movement, or release into the environment of geneticatl y engineered plant pests. The regulations require that a person obtain a permit from APHIS prior to introducing a regulated article. This letter serves to give notice to and affords the State of Arizona the opportunity to indicate concurrence or non-concurrence with APHIS' assessment that contained field testing of these genetically modified insects does not pose a plant risk. You may also provide any conditions that may be mandated by your State. Please review the enclosed documents and return acknowledgement, associated comments, or reasons for non-concurrence (if applicable) to APHIS within 30 days from the date of this letter or preferably sooner (please use the enclosed form; use additional sheets for response, if needed).

Please refer to permit No. 05-118-01r in your correspondence regarding this application. If you have any questions about this application, please contact me at (301) 734-5720, facsimile (301) 734-8669, or e-mail: john.j.peloquin@aphis.usda.gov.

APHIS hopes to maintain its excellent working relationship with your State and encourages your participation and comments prior to our final decision regarding this permit application.

Sincerely, John Joseph Peloquin

John J. Peloquin, Ph.D.
Supervisory Biotechnologist/Entomologist
Animals Branch Chief
Biotechnology Regulatory Services

Enclosures:
Permit Application No. 05-118-01r
State Response Form

Cc:
S. Wellstood, Compliance Branch, Rivderdale, MD 20737
File 05-118-01r

APHIS:BRS:JP:hll:x8231:6/27/2005:0511801r

USDA

United States Department of Agriculture

Marketing and Regulatory Programs

Animal and Plant Health Inspection Service

4700 River Road
Riverdale, MD 20737

August 2, 2005

Dr. Gregory S. Simmons
USDA, APHIS, PPQ, CPHST, DSPMSL
3645 E. Wier Avenue
Phoenix, AZ 85040

Dear Dr. Simmons:

Subject: Biotechnology Permit Number 05-118-01r to Conduct a Planned Release of Genetically Engineered *Pectinophora gossypiella*

The above permit has been approved and you must adhere to the standard and supplemental conditions enclosed.

This permit should not be taken as any type of efficacy determination of the genetically engineered organisms.

Neil Hoffman for R.B

Rebecca Bech
Associate Deputy Administrator
Biotechnology Regulatory Services

Enclosures:
Permit 05-118-01r
Supplemental Permit Conditions
Standard Permit Conditions
Map - Regional Biotechnologists

cc:
D. Madison, Arizona Department of Agriculture, Phoenix, AZ 85007
S. Wellstood, Compliance, BRS, Riverdale, MD 20737
File 05-118-01r

APHIS:BRS:JP:hll:8/1/2005:0511801r

APHIS - Protecting American Agriculture
An Equal Opportunity Employer

Sent 8/3/05

SUPPLEMENTAL PERMIT CONDITIONS
05-118-01R

Reviewed/Approved: 7/29/05 JPP
REVISED/CORRECTED: 8/6/2005 JPP

1. Studies with males and females will be conducted in 3.6 x 7.3 x 1.8m screen field cages placed over cotton plants at the CPHST rearing facility.

2. The site will be surrounded by an 8 foot chain link fence, topped with razor wire with locked gates, video surveillance and limited entry authorization. It is at least 3 miles from the nearest cultivated cotton. The structure of the field cages is 2.54 cm galvanized pipe covered with a 16 x 16 mesh (256 openings per square inch) fiberglass screen with reinforced corners to prevent tears. The cages also have a 30.5 cm plastic skirt running along the bottom that is buried in the soil to prevent moth escapes.

3. An alternative site for confined studies is the same site used under permit 01-029-01r. This alternative site is surrounded by a 6 foot chain link fence.

4. Eight pheromone traps baited with 2 mg of Gossyplure™ will be strategically distributed around the cage area to capture any males that might escape from the cages. All cotton plants exposed to transgenic moths will be contained in the cages. Once the experimental work is done, all fruiting forms on the cotton will be removed and frozen at -20° C for 24 hours to eliminate all PBW life stages.

5. PBW control strategies should be in place and ready for deployment. They will include pesticides and application equipment that have been used or are currently being used to contain and/or control PBW populations.

6. Transgenic PBW that are no longer needed will be disposed of by freezing at -20° C for 24 hours. This will not destroy any life stage of this insect. All plant fruiting forms in the release cages will be frozen at -20° C for 24 hours when the study is completed. This treatment will destroy PBW life stages that may infest the fruiting forms.

4/28

This application is authorized by the Federal Plant Pest Act (7 U.S.C. 150aa et seq. and the Plant Quarantine Act (7 U.S.C. 151 et seq.)). The information will be used to determine eligibility to receive all types of permits. No permit shall be issued until this application has been approved.

See reverse side for additional information

FORM APPROVED
OMB NO. -579-0085

U.S. DEPARTMENT OF AGRICULTURE
BIOTECHNOLOGY, BIOLOGICS, AND ENVIRONMENTAL PROTECTION

APPLICATION FOR PERMIT OR COURTESY PERMIT UNDER 7 CFR 340

(Genetically Engineered Organisms or Products)

INSTRUCTIONS: Complete this form and enclose the supporting materials listed on the reverse side. See page 3 for detailed instructions.

1. NAME AND ADDRESS OF APPLICANT

Gregory S. Simmons, Ph.D.
USDA-APHIS-PPQ-CPHST-DSPMSL
3645 E. Wier Ave
Phoenix, AZ 85040

2. PERMIIT REQUESTED ("X" one)

- [ ] Limited - Interstate Movement
- [ ] Limited - Importation
- [x] Release into the Environment
- [ ] Courtesy Permit

3. THIS REQUEST IS ("X" one)

- [x] New
- [ ] Renewal
- [ ] Supplemental

4. TELEPHONE NUMBER

Area Code (602) 437-1295 05-118-01r

5. MEANS OF MOVEMENT

- [ ] Mail
- [ ] Common Carrier (FedEx)
- [ ] Baggage or Handcarried

By whom ______

6. GIVE THE FOLLOWING (IF APPLICABLE) (IF MORE SPACE IS NEEDED, ATTACH ADDITIONAL SHEET)

	Scientific Name	Common Name	Trade Name	Other Designation
a. Donor Organism:	Aequorea victoria (jellyfish), Discosoma sp. (coral) Drosophila melanogaster (vinegar fly), Escherichia coli (bacterium), Herpes simplex (virus); OR, alternatively: Oryctolagus cuniculus (rabbit), Bombyx mori (silk moth), Aequorea victoria (jellyfish),Discosoma sp. (coral) Drosophila melanogaster (vinegar fly), Esherichia coli (bacterium), Simaian virus 40 (SV40)-(virus), Cytomegalovirus (CMV),(virus), Herpes simplex (virus), Synthetic.			
b. Recipient Organism:	Pectinophora gossypiella	(pink bollworm)	N/A	N/A
c. Vector or Vector Agent:	piggyBac	N/A	N/A	N/A
d. Regulated Organism or Product:	Pectinophora gossypiella	(pink bollworm)	N/A	N/A
e. If product, list names of constituents:	N/A	N/A	N/A	N/A

7. QUANTITY OF REGULATED ARTICLE TO BE INTRODUCED AND PROPOSED SCHECULE AND NUMBER OF INTRODUCTIONS

Multiple releases of 3,600 adults/wk for up to 20 wks for a total of 72,000 insects.

8. DATE (or inclusive dates of period) OF IMPORTATION, INTERSTATE MOVEMENT, OR RELEASE

For one year, intermittent year approval of permit

9. COUNTRY OR POINT OF ORIGIN OF THE REGULATED ARTICLE

Parent stock USDA-Phoenix,AZ transformed at the University of California or at Oxitech LTD, Oxford, UK.

10. PORT OF ARRIVAL, DESTINATION OF MOVEMENT, OR SPECIFIC LOCATION OF RELEASE

Contained Field Cage Trial in Phoenix, AZ

11. ANY BIOLOGICAL MATERIAL *(e.g., culture medium, or host material)* **ACCOMPANYING THE REGULATED ARTICLE DURING MOVEMENT**

N/A.

12. APPLICANTS FOR A COURTESY PERMIT - STATE WHY YOU BELIEVE THE ORGANISM OR PRODUCT DOES NOT COME WITHIN THE DEFINITION OF A REGULATED ARTICLE

N/A.

13. SEE REVERSE SIDE

I hereby certify that the information in the application and all attachments is complete and accurate to the best of my knowledge and belief.

False Statement: Falsification of any item on this application may result in a fine of not more than $10,000 or imprisonment for not more than 5 years or both. (18 U.S.C. 1001)

14. SIGNATURE OF RESPONSIBLE PERSON

[signature]

15. PRINTED NAME AND TITLE

Gregory S. Simmons, Ph.D.

16. DATE

04/27/2005

FOR APHIS USE ONLY

State Nofitication Letter Sent	State Review Received	Permit Issued
June 28, 2005	July 8, 2005	[x] Yes [ ] No

Date of Determination	Permit No.	No. of Permit Labels Issued	Supplemental Conditions Enclosed
August 2, 2005	05-118-01r	NA	[x] Yes [ ] No

Signature of BBEP Official	Date	Expiration Date
[signature] for RB<br>Rebecca Bech, Associate Deputy Admin., BRS	August 2, 2005	August 2, 2006

APHIS FORM 2000 (JUL 89) *Replaces PPQ Form 1001 which may be used.*

SUPPLEMENTAL PERMIT CONDITIONS
05-118-01R

Reviewed/Approved: 7/29/05 JPP
REVISED/CORRECTED: 8/6/2005 JPP

1. Studies with males and females will be conducted in 3.6 x 7.3 x 1.8m screen field cages placed over cotton plants at the CPHST rearing facility.

2. The site will be surrounded by an 8 foot chain link fence, topped with razor wire with locked gates, video surveillance and limited entry authorization. It is at least 3 miles from the nearest cultivated cotton. The structure of the field cages is 2.54 cm galvanized pipe covered with a 16 x 16 mesh (256 openings per square inch) fiberglass screen with reinforced corners to prevent tears. The cages also have a 30.5 cm plastic skirt running along the bottom that is buried in the soil to prevent moth escapes.

3. An alternative site for confined studies is the same site used under permit 01-029-01r. This alternative site is surrounded by a 6 foot chain link fence.

4. Eight pheromone traps baited with 2 mg of Gossyplure™ will be strategically distributed around the cage area to capture any males that might escape from the cages. All cotton plants exposed to transgenic moths will be contained in the cages. Once the experimental work is done, all fruiting forms on the cotton will be removed and frozen at -20° C for 24 hours to eliminate all PBW life stages.

5. PBW control strategies should be in place and ready for deployment. They will include pesticides and application equipment that have been used or are currently being used to contain and/or control PBW populations.

6. Transgenic PBW that are no longer needed will be disposed of by freezing at -20° C for 24 hours. This will not destroy any life stage of this insect. All plant fruiting forms in the release cages will be frozen at -20° C for 24 hours when the study is completed. This treatment will destroy PBW life stages that may infest the fruiting forms.

[illegible]
[illegible]

Supplemental Conditions for 05-118-01r and 05-115-01r

Studies with males and females will be conducted in 3.6 x 7.3 x 1.8 m screen field cages placed over cotton plants at the CPHST rearing facility.

The site will be surrounded by an 8 foot chain link fence, topped with razor wire with locked gates, video surveillance and limited entry authorization. It is at least 3 miles from the nearest cultivated cotton. The structure of the field cages is 2.54 cm galvanized pipe covered with a 16 x 16 mesh (256 openings per square inch) fiberglass screen with reinforced corners to prevent tears. The cages also have a 30.5 cm plastic skirt running along the bottom that is buried in the soil to prevent moth escapes.

An alternative site for confined studies is the same site used under permit 01-029-01R. This alternative site is surrounded by a 6 foot chain link fence.

Eight pheromone traps baited with 2 mg of Gossyplure™ will be strategically distributed around the cage area to capture any males that might escape from the cages.
All cotton plants exposed to transgenic moths will be contained in the cages.
Once the experimental work is done, all fruiting forms on the cotton will be removed and frozen at – 20°C for 24 hrs to eliminate all PBW life stages.

Adults, though capable of flight, will be contained in field cages. As an additional layer of containment, caged insects will have been irradiated with at least 10 kr of ^{60}Co gamma irradiation.

PBW control strategies should be in place and ready for deployment. They will include pesticides and application equipment that have been used or are currently being used to contain and/or control PBW populations.

Transgenic PBW that are no longer needed will be disposed of by freezing at -20° C for 24 hours. This will destroy any life stage of this insect. All plant fruiting forms in the release cages will be frozen at -20° C for 24 hours when the study is completed. This treatment will destroy PBW life stages that may infest the fruiting forms.

Reviewed|approved by JP 7|29|05

Standard Permit Conditions For the Introduction of a Regulated Article (7 CFR 340.4 (f))

Permit Conditions: A person who is issued a permit and his/her employees or agents shall comply with the following conditions, and any supplemental conditions which shall be listed on the permit, as deemed by the Deputy Administrator to be necessary to prevent the dissemination and establishment of plant pests:

(1) The regulated article shall be maintained and disposed of (when necessary) in a manner so as to prevent the dissemination and establishment of plant pests.

(2) All packaging material, shipping containers, and any other material accompanying the regulated article shall be treated or disposed of in such a manner so as to prevent the dissemination and establishment of plant pests.

(3) The regulated article shall be kept separate from other organisms, except as specifically allowed in the permit.

(4) The regulated article shall be maintained only in areas and premises specified in the permit.

(5) An inspector shall be allowed access, during regular business hours, to the place where the regulated article is located and to any records relating to the introduction of a regulated article.

(6) The regulated article shall, when possible, be kept identified with a label showing the name of the regulated article, and the date of importation.

(7) The regulated article shall be subject to the application of measures determined by the Administrator to be necessary to prevent the accidental or unauthorized release of the regulated article.

(8) The regulated article shall be subject to the application of remedial measures (including disposal) determined by the administrator to be necessary to prevent the spread of plant pests.

(9) A person who has been issued a permit shall submit to APHIS a field test report within 6 months after the termination of the field test. A field test report shall include the APHIS reference number, methods of observation, resulting data, and analysis regarding all deleterious effects on plants, nontarget organisms, or the environment.

(10) APHIS shall be notified within the time periods and manner specified below, in the event of the following occurrences:

(i) Orally notified immediately upon discovery and notify in writing and within 24 hours in the event of any accidental or unauthroized release of the regulated article;

(ii) In writing as soon as possible but not later than within 5 working days if the regulated article or associated host organism is found to have characteristcs substantially different from those listed in the application for a permit or suffers any unusual occurrence (excessive mortality or morbidity, or unanticipated effect on non-target organisms).

(11) A permittee or his/her agent and any person who seeks to import a regulated article into the United States shall:

(i) Import or offer the regulated article for entry only at a port of entry which is designated by an asterisk in 7 CFR 319.37-14 (b);

(ii) Notify APHIS promptly upon arrival of any regulated article at a port of entry, or its arrival by such means as a manifest, customs entry document, commercial invoice, waybill, a broker's document, or a notice form provided for such purpose: and

(iii) Mark and identify the regulated article in accordance with 7 CFR 340.7.

Rev. 3/2003

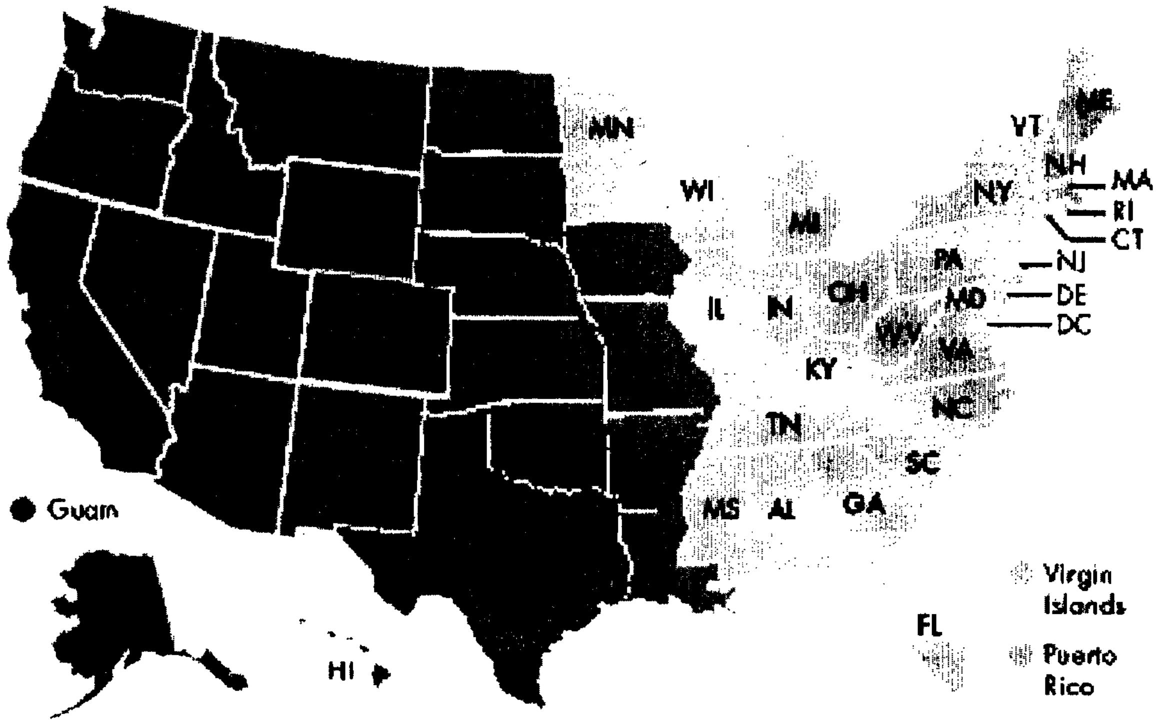

Western Region

Ralph Stoaks
USDA, APHIS, BRS
2150 Centre Avenue
Building B, 3E10
Fort Collins, CO 80526
Phone: 970-494-7573
Biotechnology Fax: 970-494-7576
Fax: 970-494-7501
E-Mail: ralph.d.stoaks@aphis.usda.gov

The western region includes the states shaded in green plus: Alaska, American Samoa, Guam, Hawaii, Mariana Islands, Marshall Islands, Micronesia, and Palau.

Eastern Region

Ashima Sengupta
USDA, APHIS, BRS
920 Main Campus Drive
Suite 200
Raleigh, NC 27606-5213
Phone: 919-855-7622
Fax: 919-855-7623
E-Mail: Ashima.Sengupta@aphis.usda.gov

The eastern region includes the states shaded in yellow plus: Virgin Islands and Puerto Rico.

	ENCLOSURES	ENCLOSED ("X")	IF PREVIOUSLY SUBMITTED, LIST DATE & PERMIT NO.
a.	Names, addresses, and telephone numbers of the persons who developed and/or supplied the regulated article.	X	
b.	A description of the anticipated or actual expression of the altered genetic material in the regulated article and how that expression differs from the expression in the nonmodified parental organism (e.g., morphological or structural characteristics, physiological activities and processes, number of copies of inserted genetic material and the physical state of this material inside the recipient organism (integrated or extrachromosomal), products and secretions, growth characteristics).	X	
c.	A detailed description of the molecular biology of the system (e.g., donor-recipient-vector) which is or will be used to produce the regulated article.	X	
d.	Country and locality where the donor organism, recipient organism, and vector or vector agent were collected, developed and produced.	X	
e.	A detailed description of the purpose of the introduction of the regulated article including a detailed description of the proposed experimental and/or production design.	X	
f.	A detailed description of the processes, procedures, and safeguards which have been used or will be used in the country of origin and in the United States toprevent contamination, release, and dissemination in the production of the: donor organism; recipient organism; vector or vector agent; constituent of each reglated article which is a product; and regulated article.	X	
g.	A detailed description of the intended destination (including final and all intermediate destinations), uses, and/or distribution of the regulated article (e.g., greenhouses, laboratory, or growth chamber location; field trial location, pilot project location; production, propagation, and manufacture location; proposed sale and distribution location).	X	
h.	A detailed description of the proposed procedures, processes, and safeguards which will be used to prevent escape and dissemination of the regulated article at each of the intended destinations.	X	
i.	A detailed description of the proposed method of final disposition of the regulated article.	X	

Public reporting burden for this collection of information is estimated to average 5 hours per response, including the time for reviewing instructions, searching existing data souces, gathering and maintaining the data needed, and completing and reviewing the collection of information. Send comments regarding this burden estimate or any other aspect of this collection of information, including suggestions for reducing this burden, to Department of Agriculture, Clearance Officer, OIRM, Room 404-W, Washington, D.C. 20250; and to the Office of Information and Regulatory Affairs, Office of Management and Budget, Washington, D.C. 20503.

ENCLOSURE A
Names, addresses, and telephone numbers of the persons who developed and/or supplied the regulated article.

(b)(6)
Department of Entomology
University of California
Riverside, CA 92521
(b)(6)

(b)(6)
Oxitec Ltd
71 Milton Park
Abingdon
Oxford, OX14 4RX
UK
(b)(6)

(b)(6)
Decision Support & Pest Management Systems Laboratory
3645 E Wier Avenue
Phoenix, AZ 85040
(b)(6)

Dr. Gregory Simmons
Entomologist
Decision Support & Pest Management Systems Laboratory
3645 E Wier Avenue
Phoenix, AZ 85040
602–437-1295 x223

(b)(6)
Pink Bollworm Rearing Facility
3645 East Chipman
Phoenix, AZ 85040
(b)(6)

ENCLOSURE B
A description of the anticipated or actual expression of the altered genetic material in the regulated article and how that expression differs from the expression in the nonmodified parental organism.

The additional genetic material in the pink bollworm comprises several protein coding regions:

1. The marker.
This allows the expression of a fluorescent protein (e.g. GFP, DsRed) originally derived from the jellyfish *Aequoria victoria* or from a coral (e.g. *Discósoma* sp.). The transgenic pink bollworm with the marker gene fluoresces when excited by illumination of the appropriate wavelength. These fluorescent proteins, which have been used as markers in a wide range of vertebrate and invertebrate species, confer no known competitive advantage or disadvantage to the recipient, and no ecological or other consequences resulting from incorporation of these markers into the transgenic pink bollworm can be envisioned. The unmodified pink bollworm is not strongly fluorescent, expression of a fluorescent protein therefore allows the modified pink bollworm to be distinguished from unmodified.

2. Tetracycline-repressible transcriptional activator (tTA).
tTA protein binds to and activates expression from the tetracycline response element (tRE), which includes multiple copies of the specific DNA sequence to which tTA binds (tetO). tTA also binds tetracycline with high affinity; the tetracycline bound form of tTA does not bind DNA. tTA therefore acts as a tetracycline regulated switch - in the absence of tetracycline it will induce expression from tRE, whereas in the presence of tetracycline it will not. High level expression of tTA is thought to be deleterious to cells as it can repress their normal transcription; low level expression has no known effect other than activation of tRE. tTA is a synthetic fusion of the tetR protein from *Escherichia coli* with VP16 from herpes simplex virus. TetR provides the tetracycline-repressible sequence-specific DNA binding property, while VP16 is a eukaryotic transcriptional activator. tTA has been used in fungi, plants, mice, mammalian culture cells, the vinegar fly *Drosophila melanogaster*, and the Mediterranean fruit fly *Ceratitis capitata*, with no known adverse effects on the environment or on human health. Unmodified pink bollworm do not have a tTA gene or similar activity.

3. Effector gene.
The effector gene encodes an insect protein or RNA, or fragment thereof, expression of which is predicted to be deleterious to the insect. For example, in the case of the LA476 construct Nipper is the central domain of the Drosophila melanogaster Nipp1Dm gene. This binds to and inhibits the catalytic subunit of type 1 serine/threonine protein phosphatase (PP1c). PP1c is an essential enzyme, therefore high level expression of Nipper (or Nipp1Dm) kills the cell. In the modified pink bollworm, Nipper is under the transcriptional control of tRE, and so is expressed when tTA is present and tetracycline is not. Unmodified pink bollworm are thought to have a Nipp1-like gene, as this protein is present in other insects, the nematode *C. elegans* and mammals.

In the case of the LA1124 construct, tTA is placed under the transcriptional control of tRE, here tTA may itself act as an effector protein. Basal expression of tTA in the modified pink bollworm is predicted to have no visible effect on the modified pink bollworm under normal laboratory rearing conditions in the presence of tetracycline. High level expression of tTA in the absence of tetracycline is predicted to be deleterious to the moth, leading to a competitive disadvantage.

No piggyBac transposase activity nor any antibiotic or pesticide resistance is conferred to the transgenic pink bollworm by the introduced genetic material.

ENCLOSURE C
A detailed description of the molecular biology of the system that was used to produce the regulated article.

PiggyBac is a DNA (deoxyribonucleic acid) transposable element that, only when its ITRs (Inverted Terminal Repeats) are intact, is capable of integrating DNA flanked by element specific DNA into other DNA through mediation of a transposase encoded by an ORF (Open Reading Frame) within the element. In the construct used for transformation of the pink bollworm, the transposase gene of the piggyBac element was irreversibly destroyed by deletion of a section of the transposase gene. Transformation was effected by introducing with the transforming construct a helper plasmid that supplied transposase activity but was itself unable to transpose into other DNA. This transposition defective helper plasmid has an ORF (Open Reading Frame) encoding piggyBac transposase under the control of the *Drosophila melanogaster* hsp70 promoter. One of the inverted terminal repeats that flank the wild type piggyBac transposase in piggyBac has been removed in the helper plasmid so that the helper plasmid cannot, itself, integrate even though it encodes for active piggyBac transposase.
The potential for instability and unwanted mobilization of piggyBac derived transforming constructs must be addressed as follows. It could be argued that if there were endogenous, piggyBac-like elements in pink bollworm, they might provide a source of transposase that could mobilize transgenes flanked by piggyBac derived ITRs. Demonstration of elements homologous to piggyBac in the recipient organism, pink bollworm, might then suggest caution regarding stability of the transgene. However, the DNA mediated element, Hermes, has been used to successfully transform *Aedes aegypti* with little or no evidence of instability of the transgenes over at least 10 generations, even though there are in *Aedes aegypti* endogenous elements (presumably hAt-like as is Hermes) with close enough homology to Hermes so that these endogenous hAt and Hermes-like elements are detected even in higher stringency Southern blots with a Hermes probe. Similarly, piggyBac has been used successfully to transform the Oriental fruit fly, *Bactrocera dorsalis*, with no evidence of instability of the transgenes, even though closely related piggyBac-like elements were later found to be present in that species.

In the case of pink bollworm, low stringency Southern blot experiments on pink bollworm DNA with radio labeled DNA probes derived from piggyBac, which would be even more likely to detect elements with low homology to piggyBac than the higher stringency methods used in Jasinskiene, et al., 1998, were unable to detect any endogenous piggyBac-like elements. This suggests that there are no elements in pink bollworm that might reasonably be expected to mobilize a piggyBac derived transgene. In addition, excision and transposition assays were performed in pink bollworm embryos with piggyBac. This was primarily to determine if piggyBac could integrate into the pink bollworm genome. However, our results showed no transposition of piggyBac in the absence of exogenous piggyBac transposase in these transposition assays, strongly suggesting there were no unknown piggyBac-like elements in the pink bollworm genome capable of mobilizing non-autonomous piggyBac elements. We can thus be reasonably certain there would not be unexpected interactions between the components of the pink bollworm genome and the transforming construct that would result in instability of the transgenes. In any event, experiments to be performed in Phoenix after transfer of the transformed pink bollworm strains will further demonstrate the stability of the transgenes.

REFERENCES

Jasinskiene, N., Coates, C.J., Benedict, M.Q., Conrel, A.J., Rafferty, C.S., James, A.A., Collins, F.H.: Stable transformation of the Yellow Fever Mosquito, *Aedes aegypti*, with the hermes element from the housefly. Proceedings of the National Academy of Sciences of the United States of America, 1998 March 31, 95(7):3743-7.

ENCLOSURE D

Country and locality where the donor organism, recipient organism, and vector or agent were collected, developed and produced:

The United Kingdom, Oxford, the University of Oxford is where all final engineering of the transforming constructs were performed. The genes used from the donor organism and the piggyBac derived portions of the vectors used to build the transforming construct were cloned at this location.

The recipient organism—the pink bollworm, *Pectinophora gossypiella*—is an invasive insect whose origin is uncertain. It is not a native species of the Western Hemisphere though it is now endemic to the southwestern United States and Mexico, associated with commercial cotton production. Introduction of the pink bollworm into the United States appears to have been via infected cottonseed. The pink bollworm appeared in Hearn, TX in 1917, and within a decade it had spread across western Texas, New Mexico, and into Arizona by 1929. The colonies transformed at the University of California Riverside and at Oxford University, Oxford UK originated from the Pink Bollworm Rearing Facility in Phoenix, Arizona.

ENCLOSURE E

A detailed description of the purpose for the introduction of the regulated article including a detailed description of the proposed experimental design.

Pink bollworm (PBW) infestations cost U.S. cotton producers $47 million per year for direct losses and control measures (National Cotton Council, March 2005, unpublished brief). APHIS is involved in two PBW control projects using the release of sterile (SIT) PBW, *Suppression* in the Central Valley of California, and *Eradication* along with *B.t.* cotton, pheromones, and pesticides. The use of SIT will expand to 90,000 acres in 2005 when sterile releases are made in eradication program areas in Texas, New Mexico and the Juarez Valley in Mexico.

The SIT suppression program has been effective and kept the San Joaquin Valley free of PBW for 30 years at low cost. However, increased cotton production costs, worldwide competition and the increasing demands for the expanded PBW eradication program requires a more effective and lower cost program. A major limit on the efficacy of SIT as a control measure is the effect of sterilizing radiation on insect performance. Radiation has a great effect on the competitiveness and effectiveness of lepidoptera used in SIT programs and has been associated with decreased quality, competitiveness, and dispersal ability in many species (North 1975, Carpenter 1997, Bloem 1999) and lower dose radiation is associated with increased mating ability and superior sperm competitiveness (Carpenter 1997). In pink bollworm, the effects of radiation include reduced longevity, decreased sperm transfer by males, decreased sperm

June 28, 2005

Mr. David Madison
Arizona Department of Agriculture
1688 W. Adams St.

Dear Mr. Madison:

This letter accompanies permit application No. 05-118-01r submitted by Dr. Gregory S. Simmons, USDA, AHIPS, PPQ, Center for Plant Health Science and Technology, DSPMSL, Phoenix Plant Protection Center, 3645 East Wiser Avenue, Phoenix, AZ in collaboration with [redacted] (b)(6) of the center for the purpose of doing cage-contained studies of transgenic pink bollworms (PBW) on cotton. This work is similar to the work done under USDA APHIS permits No. 03-104-01R and 01-029-01R. An Environmental Assessment (EA) under the national Environmental Policy Act was conducted and a Finding of No Significant Impact (FONSI) was reached regarding to make the decision to issue APHIS permits No. 03-104-01R and 01-029-01R. Because an EA has already been conducted for these preceding permit applications, and the permit research proposed in application No. 05-118-01r submitted by Dr. Simmons is similar and equivalently contained, criteria for an EA are no longer met under 7 CFR 372.5 (d) (4) "When a confined field release of genetically engineered organisms or products involves new species or organisms or novel modifications that raise new issues". It is therefore not required to conduct another EA for this current permit application. There are no claims of confidential business information in any of the documentation.

PBW is one of the most destructive pests of cotton in the world. It was first found in the United States in 1917 and has become a pest in Texas, New Mexico, Arizona and California. Costs relating to prevention, control and yield losses have been estimated by the National Cotton Council to exceed $24 million annually. The San Joaquin Valley of California remains the last cotton growing area in the Southwest that is not generally infested with PBW. Prevention of its establishment in this valley is attributed primarily to the ongoing Sterile-Insect Technique (SIT) program established jointly in 1968 by APHIS, California Department of Food and Agriculture, and the California cotton growers.

An objective of the proposed research of this permit application is to develop a strain of PBW expressing coelenterate-derived Fluorescent Protein (EGFP or DsRed) marker genes and an autocidal effector gene construct (from Oxitec, Oxford, UK). The latter transgene will fatally disrupt the development of insects carrying this gene (particularly

the progeny of mating between transgenic insects and wild type insects) when these insects are not supplied with a specific small molecule repressor (a tetracycline derivative). These cage studies are designed to test the function and effectiveness of autocidal transgenes and to determine the effectiveness of these autocidal insects in reducing experimental populations in a fully-contained experiment. Also, this experiment will compare the mating biology of these fluorescent-protein-producing, autocidal insects to that of wild type colony insects and irradiated wild type colony insects in a fully contained experimental environment. Implementation of genetically marked autocidal insects (with fluorescent proteins) into a PBW mass-rearing SIT program could provide a more effective alternative to irradiation or could reduce the necessary radiation dose to implement SIT, thereby increasing the effectiveness of an already demonstrably successful control program for the exotic pest, PBW. Finally, a genetically marked insect will be a useful monitoring tool for field managers to determine the distribution of treated PBW and to gauge the most efficient means of doing so.

Initial studies with males and females will be conducted in 3.6 x 7.3 x 1.8 m screen field cages placed over cotton plants at the CPHST rearing facility. The site is surrounded by an 8 foot chain link fence, topped with razor wire with locked gates, video surveillance and limited entry authorization. It is at least 3 miles from the nearest cultivated cotton. Adults, though capable of flight, will be contained in field cages. The structure of the field cages is 2.54 cm galvanized pipe covered with Lumite™ Saran™ 20 x 20 mesh fiberglass screen with reinforced corners to prevent tears. This mesh is tighter than mesh used in previously contained studies and as such is even less likely to allow escapes of contained moths than the materials used in USDA APHIS permits No. 03-104-01R and 01-029-01R. Though the adult moths cannot burrow, the cages also have a 30.5 cm plastic skirt running along the bottom that is buried in the soil to prevent moth escapes through soil cracks. An alternative site for confined studies is the same site used under permit 01-029-01R. This alternative site is surrounded by a 6 foot chain link fence.

Escape from such field cages is highly unlikely barring a major weather catastrophe, which itself is likely to destroy the contained insects. Eight pheromone traps baited with 2 mg of Gossyplure™ will be strategically distributed around the cage area to capture any males that might escape from the cages. All cotton plants in the area will be contained in the cages. Once the experimental work is done, all fruiting forms on the cotton will be removed and frozen at – 20°C for 24 hrs to eliminate all PBW life stages.

PBW control strategies will be in place and ready for deployment. They include pesticides and application equipment that have been used or are currently being used to contain and/or control PBW populations. The implementation of these strategies around the field cages and pheromone monitoring and control traps will make the risks negligible for the transgenic strain of PBW to transfer its genetic components to a field population of PBW or of this strain becoming established in the field. Risk is further minimized by research which has established that laboratory rearing of over 74 generations of the transgenic PBW strain give no indication that a transgenic EGFP strain has any competitive advantage over the strains currently maintained in the pink bollworm rearing facility.

Transgenic PBW that are no longer needed will be disposed of by freezing at -20° C for 48 hours. This will destroy any life stage of this insect. All plant fruiting forms in the release cages will be frozen at -20° C for 24 hours when the study is completed. This treatment will destroy PBW life stages that may infest the fruiting forms.

Previously conducted experiments demonstrated there are no transposases in the PBW genome that mobilize *piggyBac* transposon. This fact is addressed in the EA that has already been conducted resulting in the decision to issue APHIS permits No. 03-104-01R and 01-029-01R. Since there is no identifiable direct effect of this field test on any wild plant or animal species, there is no apparent risk to any threatened or endangered species. The proposed experiments are not expected to cause any adverse environmental effects due to their physical and biological containment. PWB also has no sexually compatible relatives in the United States with which it could reproduce or hybridize.

The application was submitted pursuant to regulations found in 7 CFR Part 340 which regulate the importation, interstate movement, or release into the environment of genetically engineered plant pests. The regulations require that a person obtain a permit from APHIS prior to introducing a regulated article. This letter serves to give notice to and affords the State of Arizona the opportunity to indicate concurrence or non-concurrence with APHIS' assessment that contained field testing of these genetically modified insects does not pose a plant risk. You may also provide any conditions that may be mandated by your State. Please review the enclosed documents and return acknowledgement, associated comments, or reasons for non-concurrence (if applicable) to APHIS within 30 days from the date of this letter or preferably sooner (please use the enclosed form; use additional sheets for response, if needed).

Please refer to permit No. 05-118-01r in your correspondence regarding this application. If you have any questions about this application, please contact me at (301) 734-5720, facsimile (301) 734-8669, or e-mail: john.j.peloquin@aphis.usda.gov.

APHIS hopes to maintain its excellent working relationship with your State and encourages your participation and comments prior to our final decision regarding this permit application.

Sincerely,

John J. Peloquin, Ph. D.
Supervisory Biotechnologist/Entomologist
Animals Branch Chief
Biotechnology Regulatory Services

2 Enclosures:
Permit Application No. 05-118-01r
State Response Form

receptivity, decreased female attractiveness and decreased control efficacy (Graham et al. 1972, Flint et al. 1973, Flint et al. 1974, Flint et al. 1977, Bartlett 1978, Miller et al. 1994).

Using genetic engineering to improve PBW control technology could achieve savings and greater program efficacy with the development of a PBW strain with an autocidal or conditionally lethal gene (Fryxell 1995, Miller et al. 1997), which would eliminate the need for irradiating released insects and greatly improve the performance and longevity of released moths.

The goal of this project is to develop a pink bollworm with a conditionally lethal gene using RIDL technology (**R**elease of **I**nsects with a **D**ominant **L**ethal gene, see Thomas 2000, Peng 2005) to make use in an innovative genetic control technique known as autocidal biological control. Progeny carrying a RIDL gene die when the antibiotic chlortetracycline (CTC) is absent. CTC is a normal ingredient in the PBW mass-rearing so a PBW strain with conditionally lethality controlled by CTC is a good choice for this rearing system. This is one of the most promising autocidal control systems in development.

The purpose of this experiment is to test the function of a conditionally lethal pink bollworm based on the RIDL technology in the more realistic conditions of the cotton plant within a quarantine field cage. Current RIDL strain pink bollworm express lethal phenotypes of 60-100% when reared without tetracycline on artificial diet in laboratory rearing conditions, this rate of mortality is expected to increase under the more challenging conditions of a real plant exposed to the stress of a changing environment with the extremes in temperature in the field that are unlike laboratory conditions.

The other goal of this experiment is to estimate the reduction of a wild pink bollworm population caused by release of a RIDL pink bollworm. This treatment will be compared to the reduction in wild pink bollworm caused by release of the standard APHIS strain moth irradiated at 20 kilorads.

Data from these experiments are needed for the next phase of development of a conditionally lethal pink bollworm moth. It is critical to determine if differences between the standard SIT release and RIDL release exist. If the RIDL insect is not as good as the SIT moth, is the difference within the range of improvement possible through outcrossing and strain improvement? If the two treatments have similar control efficacy, or if the RIDL insect is more efficacious, could RIDL release rates be reduced? Lastly, data from these experiments will provide key information about the differences between the two kinds of control technology (RIDL and irradiation) that will be needed for environmental analysis of the project that may be required under the National Environmental Policy Act (NEPA).

There are three experiments planned for testing the function of RIDL pink bollworm by releasing in quarantine field cages:

Experiment 1
This experiment is designed to simulate the season long release of RIDL and 20 KR irradiated moths against a native pink bollworm population within the range of densities that would be encountered within the eradication program.

There are three treatments:

1) Release of RIDL pink bollworm adults with the LA1124 construct, as heterozygotes, homozygotes or doubly homozygous for two separate insertions of the LA1124 construct (e.g., LA1124A & LA1124B).
2) Release of APHIS strain pink bollworm adults irradiated at the standard dose of 20 kilorads.
3) A no release control.

There are five replicates of treatments 1 and 2 and 4 replicates of treatment 3 arrayed in randomized incomplete blocks. Each replicate consists of a cotton plot of four rows of cotton grown in a plot measuring 7 m x 3.5 m wide. Cotton will be planted in mid to late April when soil temperatures are appropriate for germination. After the cotton has reached the 4-8 leaf stage, each plot will be covered with a 3.6 x 7.3 x 1.8 m tall screen cage (Lumite Saran 20 x 20 mesh) placed over a one inch diameter galvanized steel pipe frame. Each cage is fitted with a double door entry and each door is secured with both a heavy duty brass zipper and Velcro flap. The side of the cages are secured to the ground by burying 0.5 m of the side flap within a 0.5 m deep trench.

When the cotton reaches the pin square stage, 20 mating pairs of wild pink bollworm moths will be released into each cage to establish the test target population. Two days later, the first release of RIDL moths and 20KR APHIS moths will be released at the rate of 600 moths (1:1 sex ratio) per cage. No further releases will be made until just before the first generation (F1) of wild PBW moths emerges, this will be estimated by a degree day model and by sampling cotton flowers to determine developmental status. Upon emergence of the F1 generation releases of 600 moths per cage per week will be made until the end season.

Releases of moths will be made early in the morning before the sun is up. RIDL moths will be supplied by the quarantine rearing facility at the CPHST laboratory in Phoenix. For each release, adult moths or pupae will be carried from the quarantine building to quarantine field cages in a plastic vial within a closed small ice chest. The chest and the vials with the moths will be opened once inside the cage with the doors sealed.

Sampling will take place every three weeks, sampling either flowers or fully developed green cotton bolls. On each sample date, a random sample of 50 flowers or bolls will be collected from each cage. Samples will be placed inside plastic boll emergence boxes (37 x 25 x 16.5 cm high) fitted with tight sealing lids, which will then be sealed with tape. These will be brought into the laboratory to allow any larvae to cut out from the bolls to pupate onto hexcel material in the bottom of the boll-box. Once pupation has occurred, the boll boxes will be opened inside the quarantine laboratory and the collected pupae will be examined with fluorescence microscopy to determine if they are RIDL or wild pink bollworm, and to score for mortality. Collected data

will be used to estimate RIDL mortality and infestation rates. A sample of non-fluorescent moths will be collected for PCR screening to test for possible dissociation of the fluorescent marker from the RIDL construct. All transgenic insects collected from cages will be destroyed by freezing at 20 ± 5° C for 48 hrs.

At the conclusion of the experiment, all plant material and insects from transgenic release cages will be destroyed by either heat treatment at 65 ± 5° C for 48 hours or by freezing at 20 ± 5° C for 48 hrs.

Experiment 2

The purpose of this experiment is to compare RIDL mortality rates obtained from the small scale experiments conducted on single cotton plants in quarantine field cages to laboratory tests conducted with artificial diet. The data will be used to estimate the percent mortality of RIDL progeny after a RIDL moth mates with a wild PBW moth. Because of the harsher and more variable conditions of the outdoor environment and the differences between cotton plants and artificial diet, RIDL progeny mortality rates may be higher when reared on a cotton plant than on artificial diet. Total mortality from all sources will need to be evaluated carefully before progress on implementing RIDL system insect can be fully developed.

This experiment will take place within one large quarantine field cage (3.6 x 7.3 x 1.8 m tall) placed over 4 rows of cotton as described above. Each experimental unit will consist of a mesh sleeve cage that fits on a 0.5 m branch of one cotton plant. At the start of an experiment a branch with several bolls will be covered with the sleeve. Release of five male moths of LA1124 (tTa effector gene only) or five male moths produced by a laboratory cross of LA476 (Nipper effector gene) by LA1124 will be placed in the cage with ten APHIS or wild collected female moths and allowed to mate and oviposit on the plants. This experiment will be repeated four times with 20 replicates per experiment.

This cross will result in the production of two genotypes, heterozygote RIDL moths and homozygous wild type moths. The progeny of these moths will be allowed to develop on the cotton plant for approximately 16 d (exact time determined by degree day model) and then all bolls in the cage will be collected and placed in boll boxes as described above.

Experiment 3

This experiment is similar to Experiment 1, but will be conducted on a much smaller scale allowing greater replication. Instead of multiple releases, only a single release of RIDL and 20 KR irradiated moths will be released against a native pink bollworm population. Lastly, release rates will vary in a geometric series to allow better estimation of the shape of the pest population under the two different control techniques.

This experiment will be conducted on a small scale in sleeve cages within a larger cage as described in experiment 2.

There are three treatments:

1) Release of RIDL pink bollworm adults with the LA1124 construct , as heterozygotes, homozygotes or doubly homozygous for two separate insertions of the LA1124 construct (e.g., LA1124A & LA1124B). Release rates will range in geometric progression from 2-64, there will be four replicates of each release rate.

2) Release of APHIS strain pink bollworm adults irradiated at the standard dose of 20 kilorads. Release rates will range in geometric progression from 2-64, there will be four replicates of each release rate.

3) There will be six replicates of a no release control.

For each experiment, 30 cotton branches at pin square or first bloom will be covered with a sleeve cage. Into each cage, 4 mating pairs of wild PBW and RIDL moths or 20KR APHIS at release rates of 2, 4, 8, 16, 32, and 64 moths will be made into each release cage. The progeny of these moths will be allowed to develop on the cotton plant for approximately 16 d (exact time determined by degree day model) and then all bolls in the cage will be collected and placed in boll boxes and brought into the laboratory as described above.

REFERENCES

Bartlett, A. C. 1978. Radiation-induced sterility in the pink bollworm. U. S. Dept. Agric., Sci. Edu. Admin., Agric. Rev. Manuals, ARM-W-1, 25 pp.

Bloem, S., Bloem,K. A., Carpenter, .J.,E., Calkins, C., O. 1999. Inherited sterility in codling moth (Lepidoptera: Tortricidae): effect of substerilizing doses of radiation on insect fecundity, fertility, and control. Annals-of-the-Entomological-Society-of-America. 1999; 92(2): 222-229 92: 222-229.

Carpenter, J. E., Hidrayani, N. Nelley,and B.G. Mullinix. 1997. Effect of substerilizing doses of radiation on sperm precedence in fall armyworm (Lepidoptera: Noctuidae). Journal of economic entomology. J. econ. entomol. Apr 90: 444-448.

Flint, H. M., R. T. Staten, and B. Wright. 1977. Irradiation of pink bollworm with substerilizing doses: Production of F1 progeny. Southwest. Entomol. 2: 16-19.

Flint, H. M., R. T. Staten, L. A. Bariola, and D. L. Palmer. 1973. Gamma-irradiated pink bollworms: Attractiveness, mating, and longevity of females. Environ. Entomol. 2: 97-100.

Flint, H. M., D. L. Palmer, L. A. Bariola, and B. Horn. 1974. Suppression of populations of native pink bollworm in field cages by the release of irradiated moths. J. Econ. Entomol. 67: 55-57.

Fryxell, K. J., Miller, T.A. 1995. Autocidal biological control: A general strategy for insect control based on genetic transformation with a highly conserved gene. Journal of economic entomology. 88: 1221-1232.

Graham, H. M., M. T. Ouye, R. D. Garcia, and H. H. de la Rosa. 1972. Dosages of gamma irradiation for full and inherited sterility in adult pink bollworms. J. Econ. Entomol. 65: 645-650.

Miller, E., D. Keaveny, R. T. Staten, A. Lowe, and J. Bomberg. 1994. Changes in pink bollworm (Lepidoptera: Gelechiidae) sooty mutant under animal and plant health inspection service mass-rearing methodology. J. Econ. Entomol. 87: 1659-1664.

Miller, T. A., M. Robertson, and S. Thibault. 1997. Conditional lethal genes to control cotton pests, pp. 1340-1341. National Cotton Council, Memphis, TN.
North, D. T. 1975. Inherited sterility in Lepidoptera. Annual Review of Entomology 20: 167-182.
Peng, G., Epton, J.E., Fu, G., Scaife, S., Hiscox, A., Condon, K.C, Condon, G.C, Morrison, I.N., Kelly, D.W., Dafa'alla, T., Coleman, P.G. & Alphey, L. 2005. A dominant lethal genetic system for autocidal control of the Mediterranean fruitfly. Nature Biotechnology.
Thomas, D. T., Donnelly, C.A., Wood, R.J. & Alphey, L.S. 2000. Insect Population control using a dominant repressible, lethal genetic system. Science 287: 2474-2476.

Enclosure F

A detailed description of the processes, procedures and safeguards which have been used or will be used in the country of origin and in the US to prevent contamination, release, and dissemination in the production of the donor organism; recipient organism; vector or vector agent; constituent of each regulated article which is a product and regulated article.

Movement of transgenic pink bollworm from the quarantine rearing facility to the field quarantine release cages will be in 40 dram molded plastic vials fitted with plastic snap caps or in 1/2 liter paper food serving cartons fitted with a tight sealing plastic lid. Insects will be loaded into these containers inside the quarantine facility then placed into an insulated Styrofoam or plastic ice chest with a tight sealing lid to make a double sealed system for transport of the transgenic insects.

The containers holding the insects will only be opened inside the sealed quarantine field cages over the cotton plots. The cages are placed over 4 rows of cotton and measure 3.6 x 7.3 x 1.8 m tall made of Lumite Saran 20 x 20 mesh. Cage covers are supported by a one inch diameter galvanized steel pipe frame. Each cage is fitted with a double door entry and each door is secured with both a heavy duty brass zipper and Velcro flap. The side of the cages are secured to the ground by burying 0.5 m of the side flap within a 0.5 m deep trench.

Only authorized personnel are allowed entry to a cage. Entry will be through the double door system, which will be operated to eliminate accidental escape by sealing the first entry door (or exit) before opening the next door. Before opening the second door to enter or leave the caged cotton, the door will be inspected to ensure that there are no moths resting on or near the cage door. Personnel will inspect and shake their clothing before leaving to the cotton area to make sure no moths hitchhike on clothing. The same procedures will be performed before leaving the cage through the door to the outside, inspecting the flap of the door, cage walls, roof and the space around the door before exiting.

The site will be surrounded by a 9-foot chain link fence, topped with razor wire with locked gates, limited entry authorization, and televised security monitoring. Besides the physical containment and security, biological containment will include the procedures outlined below to minimize escape or dispersal of EGFP-altered PBW.

The probability of escape from field cages will be negligible, barring a major weather catastrophe. Eight pheromone traps (sticky Delta™ traps) baited with 2 mg of gossyplure will be

strategically distributed around the cage area to capture any males that might escape from the cages. Huber et al. 1979 using 11 traps/hectare, reported mass trapping an effective tool for suppressing the adult male population of PBW in a cotton field. All cotton plants will be contained inside the cages. Once the experimental work is completed, all fruiting forms on the cotton plants will be removed and frozen for 48 h at -20 ± 5° C to eliminate all PBW life forms.

In place will be several PBW control strategies including pesticides and their application equipment that have been used or are currently being used to contain and/or control PBW populations. The implementation of these strategies around our field release cages and pheromone monitoring and control traps will make the risks negligible for the transgenic strain of PBW to transfer its genetic components to a field population of PBW or becoming established in the field.

Finally, RIDL insects are designed to die without tetracycline. Laboratory testing of over 12,000 individuals has shown that mortality of RIDL insects when reared without tetracycline express lethality as high as 100% with a range of 60-100%. These are rates of mortality seen in the favorable environment of the laboratory, it is expected that mortality rates will be much higher under field cage conditions. In the unlikely event of an escape from a cage, the probability of a fertile adult female moth finding a cotton plant, and any of the progeny surviving to reproduce, is very low.

REFERENCES

Huber, R. T., L. Moore, and M. P. Hoffman. 1979. Feasibility study of area-wide pheromone trapping of male pink bollworm moths in a cotton insect pest management program. J. Econ. Entomol. 72: 222-227.

ENCLOSURE G

A detailed description of the intended destination (including final and all intermediate destinations), uses, and/or distribution of the regulated article (e.g., greenhouses, laboratory, or growth chamber location; field trial location; pilot project location; production, propagation, and manufacture location; proposed sale and distribution location).

The LA1124 strains are currently reared in a quarantine laboratory in Phoenix AZ., which is operated under APHIS permit 98–244–02m.

Transgenic insects reared in quarantine facility for field release are released in quarantine field cages placed over four rows of cotton plants. The field is located within the city of Phoenix Arizona. Each plot will be covered with a 3.6 x 7.3 x 1.8 m tall screen cage (Lumite Saran 20 x 20 mesh) placed over a one inch diameter galvanized steel pipe frame. Each cage is fitted with a double door entry and each door is secured with both a heavy duty brass zipper and Velcro flap. The side of the cages are secured to the ground by burying 0.5 m of the side flap within a 0.5 m deep trench. Access to the test plots is controlled by a locked 9 foot high security fence. The test plot area is monitored by closed circuit security cameras.

At the conclusion of the test, all transgenic pink bollworm life forms will be destroyed.

ENCLOSURE H

A detailed description of the proposed procedures, processes, and safeguards which will be used to prevent escape and dissemination of the regulated article at each of the intended destinations.

Movement of transgenic pink bollworm from the quarantine rearing facility to the field quarantine release cages will be in 40 dram molded plastic vials fitted with plastic snap caps or in 1/2 liter paper food serving cartons fitted with a tight sealing plastic lid. Insects will be loaded into these containers inside the quarantine facility then placed into an insulated Styrofoam or plastic ice chest with a tight sealing lid to make a double sealed system for transport of the transgenic insects.

The containers holding the insects will only be opened inside the sealed quarantine field cages over the cotton plots. The cages are placed over 4 rows of cotton and measure 3.6 x 7.3 x 1.8 m tall made of Lumite Saran 20 x 20 mesh. Cage covers are supported by a one inch diameter galvanized steel pipe frame. Each cage is fitted with a double door entry and each door is secured with both a heavy duty brass zipper and Velcro flap. The side of the cages are secured to the ground by burying 0.5 m of the side flap within a 0.5 m deep trench.
Only authorized personnel are allowed entry to a cage. Entry will be through the double door system, which will be operated to eliminate accidental escape by sealing the first entry door (or exit) before opening the next door. Before opening the second door to enter or leave the caged cotton, the door will be inspected to ensure that there are no moths resting on or near the cage door. Personnel will inspect and shake their clothing before leaving to the cotton area to make sure no moths hitchhike on clothing. The same procedures will be performed before leaving the cage through the door to the outside, inspecting the flap of the door, cage walls, roof and the space around the door before exiting.

Cages will be monitored daily to ensure that the structure remains intact and all closures are sealed.

ENCLOSURE I

A detailed description of the proposed method of final disposition of the regulated article.

Transgenic PBW in the laboratory that are no longer needed will be disposed of by freezing at $-20^{\circ} \pm 5^{\circ}$ C for 48 hours. This will destroy any life stage of this insect. Transgenic PBW recaptured in the field cage trails will be disposed of by freezing at $-20^{\circ} \pm 5^{\circ}$ C for 48 hours. All plant fruiting forms in the release cages will be disposed of by freezing at $-20^{\circ} \pm 5^{\circ}$C for 48 hours when the study is completed. This will destroy life stages that may infest the fruiting forms.

ENCLOSURE J
A description of the field trial location.

Transgenic insects reared in the Phoenix Pink Bollworm Genetic Rearing Facility selected for field release will be released in secure screened cages (3.6 × 7.3 × 1.8 m) placed over cotton plants. This location is in a highly secure security fenced area within an urban area in Phoenix, Arizona under the control of USDA authorities. No commercial cotton fields are within three miles of this field.

05-118-01R
add to permit

BRS Permits
Sent by: Linda Lightle

08/03/2005 11:27 AM

To: Gregory S Simmons/AZ/APHIS/USDA@USDA
cc: dmadison@azda.gov, John J Peloquin/MD/APHIS/USDA@USDA, Ingrid E Berlanger/MD/APHIS/USDA@USDA
Subject: Permit issuance for 05-118-01r

Dear Greg

Attached for your files is the official electronic copy of your approved permit. Please call if you have any questions or concerns.

Sincerely,

Linda

05_11801r_pil.pdf

August 2, 2005

Dr. Gregory S. Simmons
USDA, APHIS, PPQ, CPHST, DSPMSL
3645 E. Wier Avenue
Phoenix, AZ 85040

Dear Dr. Simmons:

Subject: Biotechnology Permit Number 05-118-01r to Conduct a Planned Release of Genetically Engineered *Pectinophora gossypiella*

The above permit has been approved and you must adhere to the standard and supplemental conditions enclosed.

This permit should not be taken as any type of efficacy determination of the genetically engineered organisms.

Rebecca Bech
Associate Deputy Administrator
Biotechnology Regulatory Services

Enclosures:
Permit 05-118-01r
Supplemental Permit Conditions
Standard Permit Conditions
Map - Regional Biotechnologists

cc:
D. Madison, Arizona Department of Agriculture, Phoenix, AZ 85007
S. Wellstood, Compliance, BRS, Riverdale, MD 20737
File 05-118-01r

APHIS:BRS:JP:hll:8/1/2005:0511801r

Linda Lightle

08/02/2005 05:24 PM

To: Neil E Hoffman/MD/APHIS/USDA,
cc:
Subject: Hi Neil - I have the permit and issuance letter ready for the pink bollworm release in AZ John was working on. Did you want to review the conditions and sign off for him? Thanks

APPLICATION NO.: 05-118-01R
DATE: JUNE 28, 2005

APHIS'S RESPONSE TO APHIS' INITIAL REVIEW OF AN APPLICATION FOR THE INTRODUCTION OF A REGULATED ARTICLE UNDER 7 CFR 340

- [x] State concurs with APHIS' initial review.

- [ ] State concurs with APHIS' initial review and offers the following comments (use additional sheets if necessary):

- [ ] State *does not concur* with APHIS' initial review and offers the following reasons for nonconcurrence (use additional sheets if necessary):

Name of State Official: **G. John Caravetta**

Title: **Associate Director**

Agency or Department: ARIZONA

City, State, Zip Code: **Phoenix AZ 85007**

Date: 7/5/05

PLEASE RETURN THIS FORM WITHIN 30 DAYS OF THE DATE LISTED ABOVE TO:

Linda Lightle
USDA, APHIS, PPQ, PRA
Biotechnology Evaluation
4700 River Road, Unit 147
Riverdale, MD 20737
301/734-5787/8231/Fax: 301/734-8910

JUL 8 2005
KLN

BRS Permits
Sent by: Linda Lightle

06/28/2005 04:02 PM

To: dmadison@azda.gov
cc: John J Peloquin/MD/APHIS/USDA@USDA
Subject: State letter for Permit 05-118-01r 0 PPQ - Pink Bollworm

Dear Mr. Madison

Attached for your review and approval is the state letter, permit application 05-118-01r (No CBI), and the state concurrence form for the subject permit.

Please either fax back or email your concurrence once you have reviewed the permit application and state letter. If you have any questions regarding the electronic submission of these documents please contact me on 301/734-8231.

Thank you for your patience as we continue to work through these new electronic processes.

Sincerely

Linda Lightle No. 05_11801r_scf.doc 05_11801r_sl.pdf 05_11801r_.pdf

Gregory S Simmons
07/27/2005 03:42 PM

To: Linda Lightle/MD/APHIS/USDA@USDA
cc: John J Peloquin/MD/APHIS/USDA@USDA, Ernie D Miller/AZ/APHIS/USDA
Subject: status of Permit 05-118-01r

Hello Linda,

We have heard from State of Arizona Dept of AG that they have processed my permit application 05-118-01r for release of transgenic pink bollworm into quarantine field cages and sent back to BRS. This is the pink bollworm with the RIDL gene. Can you report on the status of this permit application?

On another note, we also have heard from Arizona that permit application 05-115-01r from Ernie Miller for releases of pink bollworm with the EGFP marker gene into quarantine cages has not been reported as received by them. We understand that application was sent to the State and we are wondering about the status of this permit application as well.

Thank you,

Greg Simmons

****

Gregory Simmons, Ph.D.
USDA-APHIS-PPQ-CPHST
Decision Support & Pest Management Systems Laboratory
3645 E. Wier Avenue
Phoenix, AZ 85040
Tel: 602-437-1295 x 223
Fax: 602-437-2121
gregory.s.simmons@aphis.usda.gov
****

Linda Lightle

07/25/2005 01:14 PM

To: John J Peloquin/MD/APHIS/USDA,
cc:
Subject: 05-118-01r for APHIS Pk Bollworm - I have received state concurrence.
can I go ahead and issue or is this the permit that requires the EA?
Thanks much - Linda

USDA

United States Department of Agriculture

Marketing and Regulatory Programs

Animal and Plant Health Inspection Service

4700 River Road
Riverdale, MD 20737

June 28, 2005

Mr. David Madison
Arizona Department of Agriculture
1688 W. Adams St.
Phoenix, AZ 85007

Dear Mr. Madison:

This letter accompanies permit application No. 05-118-01r submitted by Dr. Gregory S. Simmons, USDA, AHIPS, PPQ, Center for Plant Health Science and Technology, DSPMSL, Phoenix Plant Protection Center, 3645 East Wiser Avenue, Phoenix, Arizona, in collaboration with (b)(6) of the Center for the purpose of doing cage-contained studies of transgenic pink bollworms (PBW) on cotton. This work is similar to the work done under USDA APHIS permits No. 03-104-01r and 01-029-01r. An Environmental Assessment (EA) under the national Environmental Policy Act was conducted and a Finding of No Significant Impact (FONSI) was reached regarding to make the decision to issue APHIS permits No. 03-104-01r and 01-029-01r. Because an EA has already been conducted for these preceding permit applications, and the permit research proposed in application No. 05-118-01r submitted by Dr. Simmons is similar and equivalently contained, criteria for an EA are no longer met under 7 CFR 372.5 (d) (4) "When a confined field release of genetically engineered organisms or products involves new species or organisms or novel modifications that raise new issues". It is therefore not required to conduct another EA for this current permit application. There are no claims of confidential business information in any of the documentation.

PBW is one of the most destructive pests of cotton in the world. It was first found in the United States in 1917 and has become a pest in Texas, New Mexico, Arizona and California. Costs relating to prevention, control and yield losses have been estimated by the National Cotton Council to exceed $24 million annually. The San Joaquin Valley of California remains the last cotton growing area in the Southwest that is not generally infested with PBW. Prevention of its establishment in this valley is attributed primarily to the ongoing Sterile-Insect Technique (SIT) program established jointly in 1968 by APHIS, California Department of Food and Agriculture, and the California cotton growers.

An objective of the proposed research of this permit application is to develop a strain of PBW expressing coelenterate-derived Fluorescent Protein (EGFP or DsRed) marker genes and an autocidal effector gene construct (from Oxitec, Oxford, UK). The latter transgene will fatally disrupt the development of insects carrying this gene (particularly the progeny of mating between transgenic insects and wild type insects) when these insects are not supplied with a specific small molecule repressor (a tetracycline derivative). These cage studies are designed to test the function and effectiveness of autocidal transgenes and to determine the effectiveness of these autocidal insects in reducing experimental populations in a fully-contained experiment. Also, this experiment will compare the mating biology of these fluorescent-protein-producing, autocidal insects

5 6/28/05

APPLICATION NO.: 05-118-01R
DATE: JUNE 28, 2005

APHIS'S RESPONSE TO APHIS' INITIAL REVIEW OF AN APPLICATION FOR THE INTRODUCTION OF A REGULATED ARTICLE UNDER 7 CFR 340

☐ State concurs with APHIS' initial review.

☐ State concurs with APHIS' initial review and offers the following comments (use additional sheets if necessary):

☐ State *does not concur* with APHIS' initial review and offers the following reasons for nonconcurrence (use additional sheets if necessary):

Name of State Official:

Title:

Agency or Department: ARIZONA

City, State, Zip Code:

Date:

PLEASE RETURN THIS FORM WITHIN 30 DAYS OF THE DATE LISTED ABOVE TO:

Linda Lightle
USDA, APHIS, PPQ, PRA
Biotechnology Evaluation
4700 River Road, Unit 147
Riverdale, MD 20737
301/734-5787/8231/Fax: 301/734-8910

5
6/28/05

to that of wild type colony insects and irradiated wild type colony insects in a fully contained experimental environment. Implementation of genetically marked autocidal insects (with fluorescent proteins) into a PBW mass-rearing SIT program could provide a more effective alternative to irradiation or could reduce the necessary radiation dose to implement SIT, thereby increasing the effectiveness of an already demonstrably successful control program for the exotic pest, PBW. Finally, a genetically marked insect will be a useful monitoring tool for field managers to determine the distribution of treated PBW and to gauge the most efficient means of doing so.

Initial studies with males and females will be conducted in 3.6 x 7.3 x 1.8 m screen field cages placed over cotton plants at the CPHST rearing facility. The site is surrounded by an 8 foot chain link fence, topped with razor wire with locked gates, video surveillance and limited entry authorization. It is at least 3 miles from the nearest cultivated cotton. Adults, though capable of flight, will be contained in field cages. The structure of the field cages is 2.54 cm galvanized pipe covered with Lumite™ Saran™ 20 x 20 mesh fiberglass screen with reinforced corners to prevent tears. This mesh is tighter than mesh used in previously contained studies and as such is even less likely to allow escapes of contained moths than the materials used in USDA APHIS permits No. 03-104-01R and 01-029-01R. Though the adult moths cannot burrow, the cages also have a 30.5 cm plastic skirt running along the bottom that is buried in the soil to prevent moth escapes through soil cracks. An alternative site for confined studies is the same site used under permit 01-029-01R. This alternative site is surrounded by a 6 foot chain link fence.

Escape from such field cages is highly unlikely barring a major weather catastrophe, which itself is likely to destroy the contained insects. Eight pheromone traps baited with 2 mg of Gossyplure™ will be strategically distributed around the cage area to capture any males that might escape from the cages. All cotton plants in the area will be contained in the cages. Once the experimental work is done, all fruiting forms on the cotton will be removed and frozen at – 20°C for 24 hrs to eliminate all PBW life stages.

PBW control strategies will be in place and ready for deployment. They include pesticides and application equipment that have been used or are currently being used to contain and/or control PBW populations. The implementation of these strategies around the field cages and pheromone monitoring and control traps will make the risks negligible for the transgenic strain of PBW to transfer its genetic components to a field population of PBW or of this strain becoming established in the field. Risk is further minimized by research which has established that laboratory rearing of over 74 generations of the transgenic PBW strain give no indication that a transgenic EGFP strain has any competitive advantage over the strains currently maintained in the pink bollworm rearing facility.

Transgenic PBW that are no longer needed will be disposed of by freezing at -20° C for 48 hours. This will destroy any life stage of this insect. All plant fruiting forms in the release cages

will be frozen at -20° C for 24 hours when the study is completed. This treatment will destroy PBW life stages that may infest the fruiting forms.

Previously conducted experiments demonstrated there are no transposases in the PBW genome that mobilize *piggyBac* transposon. This fact is addressed in the EA that has already been conducted resulting in the decision to issue APHIS permits No. 03-104-01R and 01-029-01R. Since there is no identifiable direct effect of this field test on any wild plant or animal species, there is no apparent risk to any threatened or endangered species. The proposed experiments are not expected to cause any adverse environmental effects due to their physical and biological containment. PWB also has no sexually compatible relatives in the United States with which it could reproduce or hybridize.

The application was submitted pursuant to regulations found in 7 CFR Part 340 which regulate the importation, interstate movement, or release into the environment of geneticat1y engineered plant pests. The regulations require that a person obtain a permit from APHIS prior to introducing a regulated article. This letter serves to give notice to and affords the State of Arizona the opportunity to indicate concurrence or non-concurrence with APHIS' assessment that contained field testing of these genetically modified insects does not pose a plant risk. You may also provide any conditions that may be mandated by your State. Please review the enclosed documents and return acknowledgement, associated comments, or reasons for non-concurrence (if applicable) to APHIS within 30 days from the date of this letter or preferably sooner (please use the enclosed form; use additional sheets for response, if needed).

Please refer to permit No. 05-118-01r in your correspondence regarding this application. If you have any questions about this application, please contact me at (301) 734-5720, facsimile (301) 734-8669, or e-mail: john.j.peloquin@aphis.usda.gov.

APHIS hopes to maintain its excellent working relationship with your State and encourages your participation and comments prior to our final decision regarding this permit application.

Sincerely,

John J. Peloquin, Ph.D.
Supervisory Biotechnologist/Entomologist
Animals Branch Chief
Biotechnology Regulatory Services

Enclosures:
Permit Application No. 05-118-01r
State Response Form

Cc:
S. Wellstood, Compliance Branch, Rivderdale, MD 20737
File 05-118-01r

APHIS:BRS:JP:hll:x8231:6/27/2005:0511801r

5/09/05 9:46 am Release Tracking Sheet

============================
Bp number: 05-118-01r
============================

Intro type: Release Renewal Permit:
Received: 4/28/05 Clock stop: Clock start: Due:
Institution: APHIS
Resp person: Simmons, Gregory
Recipient: Pink bollworm
Status: Pending Sites: 1 Acre:
Reviewer: JP
CBI status:
Phenotype:
Comments: Contained field cage trial
Parsed name: Dr. Gregory Simmons Entomologist
Address1: USDA,APHIS, PPQ
Address2: Center for Plant Health Science and Technology
Address3: 3645 E. Wier Avenue
Address4:
City/State: Phoenix, AZ 85040
Telephone: 602-437-1295 Fax:

		Initial	Date
1.	[✓] Assign Bp number and initial data entry	[illegible]	5/9
2.	[ ] Letter of notification to Applicant	[ ]	[ ]
3.	[✓] File and this tracking sheet to reviewer	[illegible]	5/9
4.	[ ] Letter of preliminary assessment to State	[ ]	[ ]
5.	[ ] Reviewer checks data entries and returns this sheet to permit unit	[ ]	[ ]
6.	[ ] State response listed by State/Region/Sites		
	AZ WR 1 State Response	[ ]	[ ]
7.	[ ] Permit issued or denied	[ ]	[ ]
8.	[ ] Enter final data into database	[ ]	[ ]

SUPPLEMENTAL PERMIT CONDITIONS
05-118-01R

Reviewed/Approved: 7/29/05 JPP
REVISED/CORRECTED: 8/6/2005 JPP

1. Studies with males and females will be conducted in 3.6 x 7.3 x 1.8m screen field cages placed over cotton plants at the CPHST rearing facility.

2. The site will be surrounded by an 8 foot chain link fence, topped with razor wire with locked gates, video surveillance and limited entry authorization. It is at least 3 miles from the nearest cultivated cotton. The structure of the field cages is 2.54 cm galvanized pipe covered with a 16 x 16 mesh (256 openings per square inch) fiberglass screen with reinforced corners to prevent tears. The cages also have a 30.5 cm plastic skirt running along the bottom that is buried in the soil to prevent moth escapes.

3. An alternative site for confined studies is the same site used under permit 01-029-01r. This alternative site is surrounded by a 6 foot chain link fence.

4. Eight pheromone traps baited with 2 mg of Gossyplure™ will be strategically distributed around the cage area to capture any males that might escape from the cages. All cotton plants exposed to transgenic moths will be contained in the cages. Once the experimental work is done, all fruiting forms on the cotton will be removed and frozen at -20◦ C for 24 hours to eliminate all PBW life stages.

5. PBW control strategies should be in place and ready for deployment. They will include pesticides and application equipment that have been used or are currently being used to contain and/or control PBW populations.

6. Transgenic PBW that are no longer needed will be disposed of by freezing at -20◦ C for 24 hours. This will not destroy any life stage of this insect. All plant fruiting forms in the release cages will be frozen at -20◦ C for 24 hours when the study is completed. This treatment will destroy PBW life stages that may infest the fruiting forms.

Gregory S Simmons
08/08/2005 02:04 PM

To: [redacted]comcast.net
cc: brspermits@aphis.usda.gov, dmadison@azda.gov, Ingrid.E.Berlanger@aphis.usda.gov, John.J.Peloquin@aphis.usda.gov, Linda.Lightle@aphis.usda.gov
Subject: Re: Permit issuance for 05-118-01r

Thank you John,

Do you have any idea on how long this will take to get done? We were hoping to start our experiment next week.
Thanks,

Greg Simmons

****
Gregory Simmons, Ph.D.
USDA-APHIS-PPQ-CPHST
Decision Support & Pest Management Systems Laboratory
3645 E. Wier Avenue
Phoenix, AZ 85040
Tel: 602-437-1295 x 223
Fax: 602-437-2121
gregory.s.simmons@aphis.usda.gov
****

[redacted]@comcast.net
08/06/2005 08:54 AM
To: Gregory.S.Simmons@aphis.usda.gov, brspermits@aphis.usda.gov
cc: dmadison@azda.gov, Ingrid.E.Berlanger@aphis.usda.gov, John.J.Peloquin@aphis.usda.gov, Linda.Lightle@aphis.usda.gov
Subject: Re: Permit issuance for 05-118-01r

Dear Greg and Linda.

It appears that I have made an error in the supplemental conditions for Gregg's work. There should not have been a requirement for irradiation of the cage contained RIDL PBW. Again, confusion due produced by similar permit application numbers contributed to this mistake. Could the supplemental condition requirement for irradiation of Gregg's PBW be removed from his permit?
-------------- Original message --------------

Linda/John:

I've received the permit for the work with the RIDL pink bollworm moths, thank you. Though there is a small problem in one of the supplemental conditions, that is the requirement for 10KR radiation treatment of the RIDL LA1124 pink bollworm (PBW) moths. RIDL-PBW moths are made to be biochemically sterile when reared without tetracycline, treatment with 10KR of radiation will make them sterile by causing lethal mutations by damaging the chromosomes. The purpose of this experiment is to measure the
stage and degree of mortality of the progeny of a RIDL-PBW moth when crossed to a wild type PBW and when self crossed. Treatment with radiation defeats the purpose of this experiment.
The attached supplemental conditions appear to be similar to the conditions that were applied to the work with pink bollworm with EGFP as a
genetic marker. In that case, sterilization of the moths is appropriate as
those insects are not designed to be biochemically sterile and their intended use is as part of a standard sterile insect release program, where insects are sterilized by radiation.
I'm wondering if the application if the same supplemental conditions for release of EGFP pink bollworm in cages and to a RIDL pink bollworm is an oversight or intentional? I 'd very much like to discuss this issue at your earliest convenience as we have about 6 weeks remaining in this season to conduct this experiment. There may be other measures that could
be applied that would satisfy any risk of escape, e.g., the double caging of moths that is part of the design for experiments numbers 2-3 in Enclosure E on pages 7 and 8 of the permit.

Sincerely,

Greg Simmons

****
Gregory Simmons, Ph.D.
USDA-APHIS-PPQ-CPHST
Decision Support & Pest Management Systems Laboratory
3645 E. Wier Avenue
Phoenix, AZ 85040
Tel: 602-437-1295 x 223
Fax: 602-437-2121
gregory.s.simmons@aphis.usda.gov
****
*

BRS Permits
Sent by: Linda Lightle
08/03/2005 08:27 AM
To: Gregory S Simmons/AZ/APHIS/USDA@USDA
cc: dmadison@azda.gov, John J Peloquin/MD/APHIS/USDA@USDA, Ingrid E Berlanger/MD/APHIS/USDA@USDA
Subject: Permit issuance for 05-118-01r

Dear Greg

Attached for your files is the official electronic copy of your approved permit. Please call if you have any questions or concerns.
Sincerely,

Linda

----- Message from Gregory.S.Simmons@aphis.usda.gov on Wed, 3 Aug 2005 17:17:03 +0000 ----- To: brspermits@aphis.usda.gov
cc: dmadison@azda.gov, Ingrid.E.Berlanger@aphis.usda.gov, John.J.Peloquin@aphis.usda.gov, Linda.Lightle@aphis.usda.gov
Subject: Re: Permit issuance for 05-118-01r

Gregory S Simmons
08/03/2005 01:16 PM

To: BRS Permits@USDA
cc: dmadison@azda.gov, Ingrid E Berlanger/MD/APHIS/USDA@USDA, John J Peloquin/MD/APHIS/USDA@USDA, Linda Lightle/MD/APHIS/USDA@USDA
Subject: Re: Permit issuance for 05-118-01r

Linda/John:

I've received the permit for the work with the RIDL pink bollworm moths, thank you. Though there is a small problem in one of the supplemental conditions, that is the requirement for 10KR radiation treatment of the RIDL LA1124 pink bollworm (PBW) moths. RIDL-PBW moths are made to be biochemically sterile when reared without tetracycline, treatment with 10KR of radiation will make them sterile by causing lethal mutations by damaging the chromosomes. The purpose of this experiment is to measure the
stage and degree of mortality of the progeny of a RIDL-PBW moth when crossed to a wild type PBW and when self crossed. Treatment with radiation defeats the purpose of this experiment.

The attached supplemental conditions appear to be similar to the conditions that were applied to the work with pink bollworm with EGFP as a
genetic marker. In that case, sterilization of the moths is appropriate as
those insects are not designed to be biochemically sterile and their intended use is as part of a standard sterile insect release program, where insects are sterilized by radiation.

I'm wondering if the application if the same supplemental conditions for release of EGFP pink bollworm in cages and to a RIDL pink bollworm is an oversight or intentional? I 'd very much like to discuss this issue at your earliest convenience as we have about 6 weeks remaining in this season to conduct this experiment. There may be other measures that could
be applied that would satisfy any risk of escape, e.g., the double caging of moths that is part of the design for experiments numbers 2-3 in Enclosure E on pages 7 and 8 of the permit.

Sincerely,

Greg Simmons

****
Gregory Simmons, Ph.D.
USDA-APHIS-PPQ-CPHST
Decision Support & Pest Management Systems Laboratory
3645 E. Wier Avenue
Phoenix, AZ 85040
Tel: 602-437-1295 x 223

Fax: 602-437-2121
gregory.s.simmons@aphis.usda.gov
****
*

BRS Permits
Sent by: Linda Lightle
08/03/2005 08:27 AM

To: Gregory S Simmons/AZ/APHIS/USDA@USDA
cc: dmadison@azda.gov, John J Peloquin/MD/APHIS/USDA@USDA, Ingrid E Berlanger/MD/APHIS/USDA@USDA
Subject: Permit issuance for 05-118-01r

Dear Greg

Attached for your files is the official electronic copy of your approved permit. Please call if you have any questions or concerns.

Sincerely,

Linda

(b)(6)comcast.net
08/06/2005 11:54 AM

To: Gregory.S.Simmons@aphis.usda.gov, brspermits@aphis.usda.gov
cc: dmadison@azda.gov, Ingrid.E.Berlanger@aphis.usda.gov, John.J.Peloquin@aphis.usda.gov, Linda.Lightle@aphis.usda.gov
Subject: Re: Permit issuance for 05-118-01r

Dear Greg and Linda.

It appears that I have made an error in the supplemental conditions for Gregg's work. There should not have been a requirement for irradiation of the cage contained RIDL PBW. Again, confusion due produced by similar permit application numbers contributed to this mistake. Could the supplemental condition requirement for irradiation of Gregg's PBW be removed from his permit?

-------------- Original message --------------

Linda/John:

I've received the permit for the work with the RIDL pink bollworm moths, thank you. Though there is a small problem in one of the supplemental conditions, that is the requirement for 10KR radiation treatment of the RIDL LA1124 pink bollworm (PBW) moths. RIDL-PBW moths are made to be biochemically sterile when reared without tetracycline, treatment with 10KR of radiation will make them sterile by causing lethal mutations by damaging the chromosomes. The purpose of this experiment is to measure the
stage and degree of mortality of the progeny of a RIDL-PBW moth when crossed to a wild type PBW and when self crossed. Treatment with radiation defeats the purpose of this experiment.
The attached supplemental conditions appear to be similar to the conditions that were applied to the work with pink bollworm with EGFP as a
genetic marker. In that case, sterilization of the moths is appropriate as
those insects are not designed to be biochemically sterile and their intended use is as part of a standard sterile insect release program, where insects are sterilized by radiation.
I'm wondering if the application if the same supplemental conditions for release of EGFP pink bollworm in cages and to a RIDL pink bollworm is an oversight or intentional? I 'd very much like to discuss this issue at your earliest convenience as we have about 6 weeks remaining in this season to conduct this experiment. There may be other measures that could
be applied that would satisfy any risk of escape, e.g., the double caging of moths that is part of the design for experiments numbers 2-3 in Enclosure E on pages 7 and 8 of the permit.

Sincerely,

Greg Simmons

****
Gregory Simmons, Ph.D.
USDA-APHIS-PPQ-CPHST
Decision Support & Pest Management Systems Laboratory
3645 E. Wier Avenue
Phoenix, AZ 85040
Tel: 602-437-1295 x 223
Fax: 602-437-2121
gregory.s.simmons@aphis.usda.gov
****
*

BRS Permits
Sent by: Linda Lightle
08/03/2005 08:27 AM
To: Gregory S Simmons/AZ/APHIS/USDA@USDA
cc: dmadison@azda.gov, John J Peloquin/MD/APHIS/USDA@USDA, Ingrid E Berlanger/MD/APHIS/USDA@USDA
Subject: Permit issuance for 05-118-01r

Dear Greg

Attached for your files is the official electronic copy of your approved permit. Please call if you have any questions or concerns.
Sincerely,

Linda

----- Message from Gregory.S.Simmons@aphis.usda.gov on Wed, 3 Aug 2005 17:17:03 +0000 -----
To: brspermits@aphis.usda.gov
cc: dmadison@azda.gov, Ingrid.E.Berlanger@aphis.usda.gov, John.J.Peloquin@aphis.usda.gov, Linda.Lightle@aphis.usda.gov
Subject: Re: Permit issuance for 05-118-01r

Gregory S Simmons/AZ/APHIS/USDA
08/08/2005 02:04 PM
To
[redacted]@comcast.net
cc
brspermits@aphis.usda.gov, dmadison@azda.gov,
Ingrid.E.Berlanger@aphis.usda.gov, John.J.Peloquin@aphis.usda.gov,
Linda.Lightle@aphis.usda.gov
bcc

Subject
Re: Permit issuance for 05-118-01r

Thank you John,

Do you have any idea on how long this will take to get done? We were hoping to start our experiment next week.
Thanks,

Greg Simmons

****
Gregory Simmons, Ph.D.
USDA-APHIS-PPQ-CPHST
Decision Support & Pest Management Systems Laboratory
3645 E. Wier Avenue
Phoenix, AZ 85040
Tel: 602-437-1295 x 223
Fax: 602-437-2121
gregory.s.simmons@aphis.usda.gov
****
*

[redacted]@comcast.net
08/06/2005 08:54 AM
To: Gregory.S.Simmons@aphis.usda.gov, brspermits@aphis.usda.gov cc: dmadison@azda.gov, Ingrid.E.Berlanger@aphis.usda.gov, John.J.Peloquin@aphis.usda.gov, Linda.Lightle@aphis.usda.gov Subject: Re: Permit issuance for 05-118-01r

Dear Greg and Linda.

It appears that I have made an error in the supplemental conditions for Gregg's work. There should not have been a requirement for irradiation of the cage contained RIDL PBW. Again, confusion due produced by similar

permit application numbers contributed to this mistake. Could the supplemental condition requirement for irradiation of Gregg's PBW be removed from his permit?
-------------- Original message --------------

Linda/John:

I've received the permit for the work with the RIDL pink bollworm moths, thank you. Though there is a small problem in one of the supplemental conditions, that is the requirement for 10KR radiation treatment of the RIDL LA1124 pink bollworm (PBW) moths. RIDL-PBW moths are made to be biochemically sterile when reared without tetracycline, treatment with 10KR of radiation will make them sterile by causing lethal mutations by damaging the chromosomes. The purpose of this experiment is to measure the
stage and degree of mortality of the progeny of a RIDL-PBW moth when crossed to a wild type PBW and when self crossed. Treatment with radiation defeats the purpose of this experiment.
The attached supplemental conditions appear to be similar to the conditions that were applied to the work with pink bollworm with EGFP as a
genetic marker. In that case, sterilization of the moths is appropriate as
those insects are not designed to be biochemically sterile and their intended use is as part of a standard sterile insect release program, where insects are sterilized by radiation.
I'm wondering if the application if the same supplemental conditions for release of EGFP pink bollworm in cages and to a RIDL pink bollworm is an oversight or intentional? I 'd very much like to discuss this issue at your earliest convenience as we have about 6 weeks remaining in this season to conduct this experiment. There may be other measures that could
be applied that would satisfy any risk of escape, e.g., the double caging of moths that is part of the design for experiments numbers 2-3 in Enclosure E on pages 7 and 8 of the permit.

Sincerely,

Greg Simmons

****
Gregory Simmons, Ph.D.
USDA-APHIS-PPQ-CPHST
Decision Support & Pest Management Systems Laboratory
3645 E. Wier Avenue
Phoenix, AZ 85040
Tel: 602-437-1295 x 223
Fax: 602-437-2121
gregory.s.simmons@aphis.usda.gov
****

BRS Permits

Sent by: Linda Lightle
08/03/2005 08:27 AM
To: Gregory S Simmons/AZ/APHIS/USDA@USDA
cc: dmadison@azda.gov, John J Peloquin/MD/APHIS/USDA@USDA, Ingrid E Berlanger/MD/APHIS/USDA@USDA Subject: Permit issuance for 05-118-01r

Dear Greg

Attached for your files is the official electronic copy of your approved permit. Please call if you have any questions or concerns.
Sincerely,

Linda

----- Message from Gregory.S.Simmons@aphis.usda.gov on Wed, 3 Aug 2005 17:17:03 +0000 ----- To: brspermits@aphis.usda.gov
cc: dmadison@azda.gov, Ingrid.E.Berlanger@aphis.usda.gov, John.J.Peloquin@aphis.usda.gov, Linda.Lightle@aphis.usda.gov
Subject: Re: Permit issuance for 05-118-01r

BRS Permits
Sent by: Linda Lightle

06/28/2005 04:02 PM

To: dmadison@azda.gov
cc: John J Peloquin/MD/APHIS/USDA@USDA
Subject: State letter for Permit 05-118-01r 0 PPQ - Pink Bollworm

Dear Mr. Madison

Attached for your review and approval is the state letter, permit application 05-118-01r (No CBI), and the state concurrence form for the subject permit.

Please either fax back or email your concurrence once you have reviewed the permit application and state letter. If you have any questions regarding the electronic submission of these documents please contact me on 301/734-8231.

Thank you for your patience as we continue to work through these new electronic processes.

Sincerely

Linda Lightle No 05_113D1r_scf doc

05_113D1r_si.pdf

05_113D1r_ pdf

BRS Permits
Sent by Linda Lightle

08/03/2005 11 27 AM

To Gregory S Simmons/AZ/APHIS/USDA@USDA
cc dmadison@azda gov, John J Peloquin/MD/APHIS/USDA@USDA, Ingrid E Berlanger/MD/APHIS/USDA@USDA
Subject Permit issuance for 05-118-01r

Dear Greg

Attached for your files is the official electronic copy of your approved permit Please call if you have any questions or concerns

Sincerely,

Linda

05_11801r_pi.pdf

BRS Permits
Sent by: Linda Lightle

08/11/2005 04:15 PM

To: Gregory S Simmons/AZ/APHIS/USDA@USDA
cc: John J Peloquin/MD/APHIS/USDA@USDA, dmadison@azda.gov, Ingrid E Berlanger/MD/APHIS/USDA@USDA
Subject: Revised/corrected permit conditions for release 05-118-01r effective 8/6/2005

Dear Dr. Simmons:

Attached for your file is the corrected version of your supplemental permit conditions revised effective 8/6/2005 by John Peloquin.

Please attach a copy of this electronic submission to your original file.

Sincerely

Linda

05-118D1r_sc_AMEND-revised 2-11-05.doc

APPLICATION NO.: 05-118-01R
DATE: JUNE 28, 2005

APHIS'S RESPONSE TO APHIS' INITIAL REVIEW OF AN APPLICATION FOR THE INTRODUCTION OF A REGULATED ARTICLE UNDER 7 CFR 340

☐ State concurs with APHIS' initial review.

☐ State concurs with APHIS' initial review and offers the following comments (use additional sheets if necessary):

☐ State *does not concur* with APHIS' initial review and offers the following reasons for nonconcurrence (use additional sheets if necessary):

Name of State Official:

Title:

Agency or Department: ARIZONA

City, State, Zip Code:

Date:

.

PLEASE RETURN THIS FORM WITHIN 30 DAYS OF THE DATE LISTED ABOVE TO:

Linda Lightle
USDA, APHIS, PPQ, PRA
Biotechnology Evaluation
4700 River Road, Unit 147
Riverdale, MD 20737
301/734-5787/8231/Fax: 301/734-8910

June 28, 2005

Mr. David Madison
Arizona Department of Agriculture
1688 W. Adams St.
Phoenix, AZ 85007

Dear Mr. Madison:

This letter accompanies permit application No. 05-118-01r submitted by Dr. Gregory S. Simmons, USDA, AHIPS, PPQ, Center for Plant Health Science and Technology, DSPMSL, Phoenix Plant Protection Center, 3645 East Wiser Avenue, Phoenix, Arizona, in collaboration with (b)(6) of the Center for the purpose of doing cage-contained studies of transgenic pink bollworms (PBW) on cotton. This work is similar to the work done under USDA APHIS permits No. 03-104-01r and 01-029-01r. An Environmental Assessment (EA) under the national Environmental Policy Act was conducted and a Finding of No Significant Impact (FONSI) was reached regarding to make the decision to issue APHIS permits No. 03-104-01r and 01-029-01r. Because an EA has already been conducted for these preceding permit applications, and the permit research proposed in application No. 05-118-01r submitted by Dr. Simmons is similar and equivalently contained, criteria for an EA are no longer met under 7 CFR 372.5 (d) (4) "When a confined field release of genetically engineered organisms or products involves new species or organisms or novel modifications that raise new issues". It is therefore not required to conduct another EA for this current permit application. There are no claims of confidential business information in any of the documentation.

PBW is one of the most destructive pests of cotton in the world. It was first found in the United States in 1917 and has become a pest in Texas, New Mexico, Arizona and California. Costs relating to prevention, control and yield losses have been estimated by the National Cotton Council to exceed $24 million annually. The San Joaquin Valley of California remains the last cotton growing area in the Southwest that is not generally infested with PBW. Prevention of its establishment in this valley is attributed primarily to the ongoing Sterile-Insect Technique (SIT) program established jointly in 1968 by APHIS, California Department of Food and Agriculture, and the California cotton growers.

An objective of the proposed research of this permit application is to develop a strain of PBW expressing coelenterate-derived Fluorescent Protein (EGFP or DsRed) marker genes and an autocidal effector gene construct (from Oxitec, Oxford, UK). The latter transgene will fatally disrupt the development of insects carrying this gene (particularly the progeny of mating between transgenic insects and wild type insects) when these insects are not supplied with a specific small molecule repressor (a tetracycline derivative). These cage studies are designed to test the function and effectiveness of autocidal transgenes and to determine the effectiveness of these autocidal insects in reducing experimental populations in a fully-contained experiment. Also, this experiment

will compare the mating biology of these fluorescent-protein-producing, autocidal insects to that of wild type colony insects and irradiated wild type colony insects in a fully contained experimental environment. Implementation of genetically marked autocidal insects (with fluorescent proteins) into a PBW mass-rearing SIT program could provide a more effective alternative to irradiation or could reduce the necessary radiation dose to implement SIT, thereby increasing the effectiveness of an already demonstrably successful control program for the exotic pest, PBW. Finally, a genetically marked insect will be a useful monitoring tool for field managers to determine the distribution of treated PBW and to gauge the most efficient means of doing so.

Initial studies with males and females will be conducted in 3.6 x 7.3 x 1.8 m screen field cages placed over cotton plants at the CPHST rearing facility. The site is surrounded by an 8 foot chain link fence, topped with razor wire with locked gates, video surveillance and limited entry authorization. It is at least 3 miles from the nearest cultivated cotton. Adults, though capable of flight, will be contained in field cages. The structure of the field cages is 2.54 cm galvanized pipe covered with Lumite™ Saran™ 20 x 20 mesh fiberglass screen with reinforced corners to prevent tears. This mesh is tighter than mesh used in previously contained studies and as such is even less likely to allow escapes of contained moths than the materials used in USDA APHIS permits No. 03-104-01R and 01-029-01R. Though the adult moths cannot burrow, the cages also have a 30.5 cm plastic skirt running along the bottom that is buried in the soil to prevent moth escapes through soil cracks. An alternative site for confined studies is the same site used under permit 01-029-01R. This alternative site is surrounded by a 6 foot chain link fence.

Escape from such field cages is highly unlikely barring a major weather catastrophe, which itself is likely to destroy the contained insects. Eight pheromone traps baited with 2 mg of Gossyplure™ will be strategically distributed around the cage area to capture any males that might escape from the cages. All cotton plants in the area will be contained in the cages. Once the experimental work is done, all fruiting forms on the cotton will be removed and frozen at – 20°C for 24 hrs to eliminate all PBW life stages.

PBW control strategies will be in place and ready for deployment. They include pesticides and application equipment that have been used or are currently being used to contain and/or control PBW populations. The implementation of these strategies around the field cages and pheromone monitoring and control traps will make the risks negligible for the transgenic strain of PBW to transfer its genetic components to a field population of PBW or of this strain becoming established in the field. Risk is further minimized by research which has established that laboratory rearing of over 74 generations of the transgenic PBW strain give no indication that a transgenic EGFP strain has any competitive advantage over the strains currently maintained in the pink bollworm rearing facility.

Transgenic PBW that are no longer needed will be disposed of by freezing at -20° C for 48 hours. This will destroy any life stage of this insect. All plant fruiting forms in the release cages will be frozen at -20° C for 24 hours when the study is completed. This treatment will destroy PBW life stages that may infest the fruiting forms.

Previously conducted experiments demonstrated there are no transposases in the PBW genome that mobilize *piggyBac* transposon. This fact is addressed in the EA that has already been conducted resulting in the decision to issue APHIS permits No. 03-104-01R and 01-029-01R. Since there is no identifiable direct effect of this field test on any wild plant or animal species, there is no apparent risk to any threatened or endangered species. The proposed experiments are not expected to cause any adverse environmental effects due to their physical and biological containment. PWB also has no sexually compatible relatives in the United States with which it could reproduce or hybridize.

The application was submitted pursuant to regulations found in 7 CFR Part 340 which regulate the importation, interstate movement, or release into the environment of geneticat1y engineered plant pests. The regulations require that a person obtain a permit from APHIS prior to introducing a regulated article. This letter serves to give notice to and affords the State of Arizona the opportunity to indicate concurrence or non-concurrence with APHIS' assessment that contained field testing of these genetically modified insects does not pose a plant risk. You may also provide any conditions that may be mandated by your State. Please review the enclosed documents and return acknowledgement, associated comments, or reasons for non-concurrence (if applicable) to APHIS within 30 days from the date of this letter or preferably sooner (please use the enclosed form; use additional sheets for response, if needed).

Please refer to permit No. 05-118-01r in your correspondence regarding this application. If you have any questions about this application, please contact me at (301) 734-5720, facsimile (301) 734-8669, or e-mail: john.j.peloquin@aphis.usda.gov.

APHIS hopes to maintain its excellent working relationship with your State and encourages your participation and comments prior to our final decision regarding this permit application.

Sincerely,

John J. Peloquin, Ph.D.
Supervisory Biotechnologist/Entomoligst
Animals Branch Chief
Biotechnology Regulatory Services

Enclosures:
Permit Application No. 05-118-01r
State Response Form

Cc:
S. Wellstood
File 05-118-01r

APHIS:BRS:JP:hll:x8231:6/29/05:0511801rAZ

Gregory S Simmons
08/08/2005 02:04 PM

To: (b)(6)@comcast.net
cc: brspermits@aphis.usda.gov, dmadison@azda.gov, Ingrid.E.Berlanger@aphis.usda.gov, John.J.Peloquin@aphis.usda.gov, Linda.Lightle@aphis.usda.gov
Subject: Re: Permit issuance for 05-118-01r

Thank you John,

Do you have any idea on how long this will take to get done? We were hoping to start our experiment next week.
Thanks,

Greg Simmons

****
Gregory Simmons, Ph.D.
USDA-APHIS-PPQ-CPHST
Decision Support & Pest Management Systems Laboratory
3645 E. Wier Avenue
Phoenix, AZ 85040
Tel: 602-437-1295 x 223
Fax: 602-437-2121
gregory.s.simmons@aphis.usda.gov
****

(b)(6)@comcast.net
08/06/2005 08:54 AM
To: Gregory.S.Simmons@aphis.usda.gov, brspermits@aphis.usda.gov
cc: dmadison@azda.gov, Ingrid.E.Berlanger@aphis.usda.gov, John.J.Peloquin@aphis.usda.gov, Linda.Lightle@aphis.usda.gov
Subject: Re: Permit issuance for 05-118-01r

Dear Greg and Linda.

It appears that I have made an error in the supplemental conditions for Gregg's work. There should not have been a requirement for irradiation of the cage contained RIDL PBW. Again, confusion due produced by similar permit application numbers contributed to this mistake. Could the supplemental condition requirement for irradiation of Gregg's PBW be removed from his permit?
-------------- Original message --------------

Linda/John:

I've received the permit for the work with the RIDL pink bollworm moths, thank you. Though there is a small problem in one of the supplemental conditions, that is the requirement for 10KR radiation treatment of the RIDL LA1124 pink bollworm (PBW) moths. RIDL-PBW moths are made to be biochemically sterile when reared without tetracycline, treatment with 10KR of radiation will make them sterile by causing lethal mutations by damaging the chromosomes. The purpose of this experiment is to measure the
stage and degree of mortality of the progeny of a RIDL-PBW moth when crossed to a wild type PBW and when self crossed. Treatment with radiation defeats the purpose of this experiment.
The attached supplemental conditions appear to be similar to the conditions that were applied to the work with pink bollworm with EGFP as a
genetic marker. In that case, sterilization of the moths is appropriate as
those insects are not designed to be biochemically sterile and their intended use is as part of a standard sterile insect release program, where insects are sterilized by radiation.
I'm wondering if the application if the same supplemental conditions for release of EGFP pink bollworm in cages and to a RIDL pink bollworm is an oversight or intentional? I 'd very much like to discuss this issue at your earliest convenience as we have about 6 weeks remaining in this season to conduct this experiment. There may be other measures that could
be applied that would satisfy any risk of escape, e.g., the double caging of moths that is part of the design for experiments numbers 2-3 in Enclosure E on pages 7 and 8 of the permit.

Sincerely,

Greg Simmons

****
Gregory Simmons, Ph.D.
USDA-APHIS-PPQ-CPHST
Decision Support & Pest Management Systems Laboratory
3645 E. Wier Avenue
Phoenix, AZ 85040
Tel: 602-437-1295 x 223
Fax: 602-437-2121
gregory.s.simmons@aphis.usda.gov
****

BRS Permits
Sent by: Linda Lightle
08/03/2005 08:27 AM
To: Gregory S Simmons/AZ/APHIS/USDA@USDA
cc: dmadison@azda.gov, John J Peloquin/MD/APHIS/USDA@USDA, Ingrid E Berlanger/MD/APHIS/USDA@USDA
Subject: Permit issuance for 05-118-01r

Dear Greg

Attached for your files is the official electronic copy of your approved permit. Please call if you have any questions or concerns.
Sincerely,

Linda

----- Message from Gregory.S.Simmons@aphis.usda.gov on Wed, 3 Aug 2005 17:17:03 +0000 ----- To: brspermits@aphis.usda.gov
cc: dmadison@azda.gov, Ingrid.E.Berlanger@aphis.usda.gov, John.J.Peloquin@aphis.usda.gov, Linda.Lightle@aphis.usda.gov
Subject: Re: Permit issuance for 05-118-01r

Gregory S Simmons
08/03/2005 01:16 PM

To: BRS Permits@USDA
cc: dmadison@azda.gov, Ingrid E Berlanger/MD/APHIS/USDA@USDA, John J Peloquin/MD/APHIS/USDA@USDA, Linda Lightle/MD/APHIS/USDA@USDA
Subject: Re: Permit issuance for 05-118-01r

Linda/John:

I've received the permit for the work with the RIDL pink bollworm moths, thank you. Though there is a small problem in one of the supplemental conditions, that is the requirement for 10KR radiation treatment of the RIDL LA1124 pink bollworm (PBW) moths. RIDL-PBW moths are made to be biochemically sterile when reared without tetracycline, treatment with 10KR of radiation will make them sterile by causing lethal mutations by damaging the chromosomes. The purpose of this experiment is to measure the stage and degree of mortality of the progeny of a RIDL-PBW moth when crossed to a wild type PBW and when self crossed. Treatment with radiation defeats the purpose of this experiment.

The attached supplemental conditions appear to be similar to the conditions that were applied to the work with pink bollworm with EGFP as a genetic marker. In that case, sterilization of the moths is appropriate as those insects are not designed to be biochemically sterile and their intended use is as part of a standard sterile insect release program, where insects are sterilized by radiation.

I'm wondering if the application if the same supplemental conditions for release of EGFP pink bollworm in cages and to a RIDL pink bollworm is an oversight or intentional? I 'd very much like to discuss this issue at your earliest convenience as we have about 6 weeks remaining in this season to conduct this experiment. There may be other measures that could be applied that would satisfy any risk of escape, e.g., the double caging of moths that is part of the design for experiments numbers 2-3 in Enclosure E on pages 7 and 8 of the permit.

Sincerely,

Greg Simmons

****
Gregory Simmons, Ph.D.
USDA-APHIS-PPQ-CPHST
Decision Support & Pest Management Systems Laboratory
3645 E. Wier Avenue
Phoenix, AZ 85040
Tel: 602-437-1295 x 223

Fax: 602-437-2121
gregory.s.simmons@aphis.usda.gov
****
*

BRS Permits
Sent by: Linda Lightle
08/03/2005 08:27 AM

To: Gregory S Simmons/AZ/APHIS/USDA@USDA
cc: dmadison@azda.gov, John J Peloquin/MD/APHIS/USDA@USDA, Ingrid E Berlanger/MD/APHIS/USDA@USDA
Subject: Permit issuance for 05-118-01r

Dear Greg

Attached for your files is the official electronic copy of your approved permit. Please call if you have any questions or concerns.

Sincerely,

Linda

(b)(6)comcast.net
08/06/2005 11:54 AM

To: Gregory.S.Simmons@aphis.usda.gov, brspermits@aphis.usda.gov
cc: dmadison@azda.gov, Ingrid.E.Berlanger@aphis.usda.gov, John.J.Peloquin@aphis.usda.gov, Linda.Lightle@aphis.usda.gov
Subject: Re: Permit issuance for 05-118-01r

Dear Greg and Linda.

It appears that I have made an error in the supplemental conditions for Gregg's work. There should not have been a requirement for irradiation of the cage contained RIDL PBW. Again, confusion due produced by similar permit application numbers contributed to this mistake. Could the supplemental condition requirement for irradiation of Gregg's PBW be removed from his permit?

-------------- Original message --------------

Linda/John:

I've received the permit for the work with the RIDL pink bollworm moths, thank you. Though there is a small problem in one of the supplemental conditions, that is the requirement for 10KR radiation treatment of the RIDL LA1124 pink bollworm (PBW) moths. RIDL-PBW moths are made to be biochemically sterile when reared without tetracycline, treatment with 10KR of radiation will make them sterile by causing lethal mutations by damaging the chromosomes. The purpose of this experiment is to measure the stage and degree of mortality of the progeny of a RIDL-PBW moth when crossed to a wild type PBW and when self crossed. Treatment with radiation defeats the purpose of this experiment.
The attached supplemental conditions appear to be similar to the conditions that were applied to the work with pink bollworm with EGFP as a genetic marker. In that case, sterilization of the moths is appropriate as those insects are not designed to be biochemically sterile and their intended use is as part of a standard sterile insect release program, where insects are sterilized by radiation.
I'm wondering if the application if the same supplemental conditions for release of EGFP pink bollworm in cages and to a RIDL pink bollworm is an oversight or intentional? I 'd very much like to discuss this issue at your earliest convenience as we have about 6 weeks remaining in this season to conduct this experiment. There may be other measures that could be applied that would satisfy any risk of escape, e.g., the double caging of moths that is part of the design for experiments numbers 2-3 in Enclosure E on pages 7 and 8 of the permit.

Sincerely,

Greg Simmons

****
Gregory Simmons, Ph.D.
USDA-APHIS-PPQ-CPHST
Decision Support & Pest Management Systems Laboratory
3645 E. Wier Avenue
Phoenix, AZ 85040
Tel: 602-437-1295 x 223
Fax: 602-437-2121
gregory.s.simmons@aphis.usda.gov
****
*

BRS Permits
Sent by: Linda Lightle
08/03/2005 08:27 AM
To: Gregory S Simmons/AZ/APHIS/USDA@USDA
cc: dmadison@azda.gov, John J Peloquin/MD/APHIS/USDA@USDA, Ingrid E Berlanger/MD/APHIS/USDA@USDA Subject: Permit issuance for 05-118-01r

Dear Greg

Attached for your files is the official electronic copy of your approved permit. Please call if you have any questions or concerns.
Sincerely,

Linda

----- Message from Gregory.S.Simmons@aphis.usda.gov on Wed, 3 Aug 2005 17:17:03 +0000 -----
To: brspermits@aphis.usda.gov
cc: dmadison@azda.gov, Ingrid.E.Berlanger@aphis.usda.gov, John.J.Peloquin@aphis.usda.gov, Linda.Lightle@aphis.usda.gov
Subject: Re: Permit issuance for 05-118-01r

Gregory S Simmons/AZ/APHIS/USDA
08/08/2005 02:04 PM
To
[redacted]comcast.net
cc
brspermits@aphis.usda.gov, dmadison@azda.gov,
Ingrid.E.Berlanger@aphis.usda.gov, John.J.Peloquin@aphis.usda.gov,
Linda.Lightle@aphis.usda.gov
bcc

Subject
Re: Permit issuance for 05-118-01r

Thank you John,

Do you have any idea on how long this will take to get done? We were hoping to start our experiment next week.
Thanks,

Greg Simmons

****
Gregory Simmons, Ph.D.
USDA-APHIS-PPQ-CPHST
Decision Support & Pest Management Systems Laboratory
3645 E. Wier Avenue
Phoenix, AZ 85040
Tel: 602-437-1295 x 223
Fax: 602-437-2121
gregory.s.simmons@aphis.usda.gov
****
*

[redacted]@comcast.net
08/06/2005 08:54 AM
To: Gregory.S.Simmons@aphis.usda.gov, brspermits@aphis.usda.gov cc: dmadison@azda.gov, Ingrid.E.Berlanger@aphis.usda.gov, John.J.Peloquin@aphis.usda.gov, Linda.Lightle@aphis.usda.gov Subject: Re: Permit issuance for 05-118-01r

Dear Greg and Linda.

It appears that I have made an error in the supplemental conditions for Gregg's work. There should not have been a requirement for irradiation of the cage contained RIDL PBW. Again, confusion due produced by similar

permit application numbers contributed to this mistake. Could the supplemental condition requirement for irradiation of Gregg's PBW be removed from his permit?
-------------- Original message --------------

Linda/John:

I've received the permit for the work with the RIDL pink bollworm moths, thank you. Though there is a small problem in one of the supplemental conditions, that is the requirement for 10KR radiation treatment of the RIDL LA1124 pink bollworm (PBW) moths. RIDL-PBW moths are made to be biochemically sterile when reared without tetracycline, treatment with 10KR of radiation will make them sterile by causing lethal mutations by damaging the chromosomes. The purpose of this experiment is to measure the
stage and degree of mortality of the progeny of a RIDL-PBW moth when crossed to a wild type PBW and when self crossed. Treatment with radiation defeats the purpose of this experiment.
The attached supplemental conditions appear to be similar to the conditions that were applied to the work with pink bollworm with EGFP as a
genetic marker. In that case, sterilization of the moths is appropriate as
those insects are not designed to be biochemically sterile and their intended use is as part of a standard sterile insect release program, where insects are sterilized by radiation.
I'm wondering if the application if the same supplemental conditions for release of EGFP pink bollworm in cages and to a RIDL pink bollworm is an oversight or intentional? I 'd very much like to discuss this issue at your earliest convenience as we have about 6 weeks remaining in this season to conduct this experiment. There may be other measures that could
be applied that would satisfy any risk of escape, e.g., the double caging of moths that is part of the design for experiments numbers 2-3 in Enclosure E on pages 7 and 8 of the permit.

Sincerely,

Greg Simmons

****
Gregory Simmons, Ph.D.
USDA-APHIS-PPQ-CPHST
Decision Support & Pest Management Systems Laboratory
3645 E. Wier Avenue
Phoenix, AZ 85040
Tel: 602-437-1295 x 223
Fax: 602-437-2121
gregory.s.simmons@aphis.usda.gov
****

BRS Permits

Sent by: Linda Lightle
08/03/2005 08:27 AM
To: Gregory S Simmons/AZ/APHIS/USDA@USDA
cc: dmadison@azda.gov, John J Peloquin/MD/APHIS/USDA@USDA, Ingrid E Berlanger/MD/APHIS/USDA@USDA Subject: Permit issuance for 05-118-01r

Dear Greg

Attached for your files is the official electronic copy of your approved permit. Please call if you have any questions or concerns.
Sincerely,

Linda

----- Message from Gregory.S.Simmons@aphis.usda.gov on Wed, 3 Aug 2005 17:17:03 +0000 ----- To: brspermits@aphis.usda.gov
cc: dmadison@azda.gov, Ingrid.E.Berlanger@aphis.usda.gov, John.J.Peloquin@aphis.usda.gov, Linda.Lightle@aphis.usda.gov
Subject: Re: Permit issuance for 05-118-01r

BRS Permits
Sent by: Linda Lightle

06/28/2005 04:02 PM

To: dmadison@azda.gov
cc: John J Peloquin/MD/APHIS/USDA@USDA
Subject: State letter for Permit 05-118-01r 0 PPQ - Pink Bollworm

Dear Mr. Madison

Attached for your review and approval is the state letter, permit application 05-118-01r (No CBI), and the state concurrence form for the subject permit.

Please either fax back or email your concurrence once you have reviewed the permit application and state letter. If you have any questions regarding the electronic submission of these documents please contact me on 301/734-8231.

Thank you for your patience as we continue to work through these new electronic processes.

Sincerely

Linda Lightle No 05_113D1r_scf doc 05_113D1r_si.pdf 05_113D1r_pdf

BRS Permits
Sent by: Linda Lightle

08/03/2005 11:27 AM

To: Gregory S Simmons/AZ/APHIS/USDA@USDA
cc: dmadison@azda.gov, John J Peloquin/MD/APHIS/USDA@USDA, Ingrid E Berlanger/MD/APHIS/USDA@USDA
Subject: Permit issuance for 05-118-01r

Dear Greg

Attached for your files is the official electronic copy of your approved permit. Please call if you have any questions or concerns.

Sincerely,

Linda

05_11801r_pii.pdf

BRS Permits
Sent by: Linda Lightle

08/11/2005 04:15 PM

To: Gregory S Simmons/AZ/APHIS/USDA@USDA
cc: John J Peloquin/MD/APHIS/USDA@USDA, dmadison@azda.gov, Ingrid E Berlanger/MD/APHIS/USDA@USDA
Subject: Revised/corrected permit conditions for release 05-118-01r effective 8/6/2005

Dear Dr. Simmons:

Attached for your file is the corrected version of your supplemental permit conditions revised effective 8/6/2005 by John Peloquin.

Please attach a copy of this electronic submission to your original file.

Sincerely

Linda

05-118D1r_sc_AMEND-revised 8-11-05.doc

APPLICATION NO.: 05-118-01R
DATE:JUNE 28, 2005

APHIS'S RESPONSE TO APHIS' INITIAL REVIEW OF AN APPLICATION FOR THE INTRODUCTION OF A REGULATED ARTICLE UNDER 7 CFR 340

☐ State concurs with APHIS' initial review.

☐ State concurs with APHIS' initial review and offers the following comments (use additional sheets if necessary):

☐ State *does not concur* with APHIS' initial review and offers the following reasons for nonconcurrence (use additional sheets if necessary):

Name of State Official:

Title:

Agency or Department: ARIZONA

City, State, Zip Code:

Date:

.

PLEASE RETURN THIS FORM WITHIN 30 DAYS OF THE DATE LISTED ABOVE TO:

Linda Lightle
USDA, APHIS, PPQ, PRA
Biotechnology Evaluation
4700 River Road, Unit 147
Riverdale, MD 20737
301/734-5787/8231/Fax: 301/734-8910

June 28, 2005

Mr. David Madison
Arizona Department of Agriculture
1688 W. Adams St.
Phoenix, AZ 85007

Dear Mr. Madison:

This letter accompanies permit application No. 05-118-01r submitted by Dr. Gregory S. Simmons, USDA, AHIPS, PPQ, Center for Plant Health Science and Technology, DSPMSL, Phoenix Plant Protection Center, 3645 East Wiser Avenue, Phoenix, Arizona, in collaboration with (b)(6) of the Center for the purpose of doing cage-contained studies of transgenic pink bollworms (PBW) on cotton. This work is similar to the work done under USDA APHIS permits No. 03-104-01r and 01-029-01r. An Environmental Assessment (EA) under the national Environmental Policy Act was conducted and a Finding of No Significant Impact (FONSI) was reached regarding to make the decision to issue APHIS permits No. 03-104-01r and 01-029-01r. Because an EA has already been conducted for these preceding permit applications, and the permit research proposed in application No. 05-118-01r submitted by Dr. Simmons is similar and equivalently contained, criteria for an EA are no longer met under 7 CFR 372.5 (d) (4) "When a confined field release of genetically engineered organisms or products involves new species or organisms or novel modifications that raise new issues". It is therefore not required to conduct another EA for this current permit application. There are no claims of confidential business information in any of the documentation.

PBW is one of the most destructive pests of cotton in the world. It was first found in the United States in 1917 and has become a pest in Texas, New Mexico, Arizona and California. Costs relating to prevention, control and yield losses have been estimated by the National Cotton Council to exceed $24 million annually. The San Joaquin Valley of California remains the last cotton growing area in the Southwest that is not generally infested with PBW. Prevention of its establishment in this valley is attributed primarily to the ongoing Sterile-Insect Technique (SIT) program established jointly in 1968 by APHIS, California Department of Food and Agriculture, and the California cotton growers.

An objective of the proposed research of this permit application is to develop a strain of PBW expressing coelenterate-derived Fluorescent Protein (EGFP or DsRed) marker genes and an autocidal effector gene construct (from Oxitec, Oxford, UK). The latter transgene will fatally disrupt the development of insects carrying this gene (particularly the progeny of mating between transgenic insects and wild type insects) when these insects are not supplied with a specific small molecule repressor (a tetracycline derivative). These cage studies are designed to test the function and effectiveness of autocidal transgenes and to determine the effectiveness of these autocidal insects in reducing experimental populations in a fully-contained experiment. Also, this experiment

will compare the mating biology of these fluorescent-protein-producing, autocidal insects to that of wild type colony insects and irradiated wild type colony insects in a fully contained experimental environment. Implementation of genetically marked autocidal insects (with fluorescent proteins) into a PBW mass-rearing SIT program could provide a more effective alternative to irradiation or could reduce the necessary radiation dose to implement SIT, thereby increasing the effectiveness of an already demonstrably successful control program for the exotic pest, PBW. Finally, a genetically marked insect will be a useful monitoring tool for field managers to determine the distribution of treated PBW and to gauge the most efficient means of doing so.

Initial studies with males and females will be conducted in 3.6 x 7.3 x 1.8 m screen field cages placed over cotton plants at the CPHST rearing facility. The site is surrounded by an 8 foot chain link fence, topped with razor wire with locked gates, video surveillance and limited entry authorization. It is at least 3 miles from the nearest cultivated cotton. Adults, though capable of flight, will be contained in field cages. The structure of the field cages is 2.54 cm galvanized pipe covered with Lumite™ Saran™ 20 x 20 mesh fiberglass screen with reinforced corners to prevent tears. This mesh is tighter than mesh used in previously contained studies and as such is even less likely to allow escapes of contained moths than the materials used in USDA APHIS permits No. 03-104-01R and 01-029-01R. Though the adult moths cannot burrow, the cages also have a 30.5 cm plastic skirt running along the bottom that is buried in the soil to prevent moth escapes through soil cracks. An alternative site for confined studies is the same site used under permit 01-029-01R. This alternative site is surrounded by a 6 foot chain link fence.

Escape from such field cages is highly unlikely barring a major weather catastrophe, which itself is likely to destroy the contained insects. Eight pheromone traps baited with 2 mg of Gossyplure™ will be strategically distributed around the cage area to capture any males that might escape from the cages. All cotton plants in the area will be contained in the cages. Once the experimental work is done, all fruiting forms on the cotton will be removed and frozen at – 20°C for 24 hrs to eliminate all PBW life stages.

PBW control strategies will be in place and ready for deployment. They include pesticides and application equipment that have been used or are currently being used to contain and/or control PBW populations. The implementation of these strategies around the field cages and pheromone monitoring and control traps will make the risks negligible for the transgenic strain of PBW to transfer its genetic components to a field population of PBW or of this strain becoming established in the field. Risk is further minimized by research which has established that laboratory rearing of over 74 generations of the transgenic PBW strain give no indication that a transgenic EGFP strain has any competitive advantage over the strains currently maintained in the pink bollworm rearing facility.

Transgenic PBW that are no longer needed will be disposed of by freezing at -20° C for 48 hours. This will destroy any life stage of this insect. All plant fruiting forms in the release cages will be frozen at -20° C for 24 hours when the study is completed. This treatment will destroy PBW life stages that may infest the fruiting forms.

Previously conducted experiments demonstrated there are no transposases in the PBW genome that mobilize *piggyBac* transposon. This fact is addressed in the EA that has already been conducted resulting in the decision to issue APHIS permits No. 03-104-01R and 01-029-01R. Since there is no identifiable direct effect of this field test on any wild plant or animal species, there is no apparent risk to any threatened or endangered species. The proposed experiments are not expected to cause any adverse environmental effects due to their physical and biological containment. PWB also has no sexually compatible relatives in the United States with which it could reproduce or hybridize.

The application was submitted pursuant to regulations found in 7 CFR Part 340 which regulate the importation, interstate movement, or release into the environment of geneticat1y engineered plant pests. The regulations require that a person obtain a permit from APHIS prior to introducing a regulated article. This letter serves to give notice to and affords the State of Arizona the opportunity to indicate concurrence or non-concurrence with APHIS' assessment that contained field testing of these genetically modified insects does not pose a plant risk. You may also provide any conditions that may be mandated by your State. Please review the enclosed documents and return acknowledgement, associated comments, or reasons for non-concurrence (if applicable) to APHIS within 30 days from the date of this letter or preferably sooner (please use the enclosed form; use additional sheets for response, if needed).

Please refer to permit No. 05-118-01r in your correspondence regarding this application. If you have any questions about this application, please contact me at (301) 734-5720, facsimile (301) 734-8669, or e-mail: john.j.peloquin@aphis.usda.gov.

APHIS hopes to maintain its excellent working relationship with your State and encourages your participation and comments prior to our final decision regarding this permit application.

Sincerely,

John J. Peloquin, Ph.D.
Supervisory Biotechnologist/Entomoligst
Animals Branch Chief
Biotechnology Regulatory Services

Enclosures:
Permit Application No. 05-118-01r
State Response Form

Cc:
S. Wellstood
File 05-118-01r

APHIS:BRS:JP:hll:x8231:6/29/05:0511801rAZ

June 28, 2005

Mr. David Madison
Arizona Department of Agriculture
1688 W. Adams St.

Dear Mr. Madison:

This letter accompanies permit application No. 05-118-01r submitted by Dr. Gregory S. Simmons, USDA, AHIPS, PPQ, Center for Plant Health Science and Technology, DSPMSL, Phoenix Plant Protection Center, 3645 East Wiser Avenue, Phoenix, Arizona, in collaboration with (b)(6) of the Center for the purpose of doing cage-contained studies of transgenic pink bollworms (PBW) on cotton. This work is similar to the work done under USDA APHIS permits No. 03-104-01r and 01-029-01r. An Environmental Assessment (EA) under the national Environmental Policy Act was conducted and a Finding of No Significant Impact (FONSI) was reached regarding to make the decision to issue APHIS permits No. 03-104-01r and 01-029-01r. Because an EA has already been conducted for these preceding permit applications, and the permit research proposed in application No. 05-118-01r submitted by Dr. Simmons is similar and equivalently contained, criteria for an EA are no longer met under 7 CFR 372.5 (d) (4) “When a confined field release of genetically engineered organisms or products involves new species or organisms or novel modifications that raise new issues”. It is therefore not required to conduct another EA for this current permit application. There are no claims of confidential business information in any of the documentation.

PBW is one of the most destructive pests of cotton in the world. It was first found in the United States in 1917 and has become a pest in Texas, New Mexico, Arizona and California. Costs relating to prevention, control and yield losses have been estimated by the National Cotton Council to exceed $24 million annually. The San Joaquin Valley of California remains the last cotton growing area in the Southwest that is not generally infested with PBW. Prevention of its establishment in this valley is attributed primarily to the ongoing Sterile-Insect Technique (SIT) program established jointly in 1968 by APHIS, California Department of Food and Agriculture, and the California cotton growers.

An objective of the proposed research of this permit application is to develop a strain of PBW expressing coelenterate-derived Fluorescent Protein (EGFP or DsRed) marker genes and an autocidal effector gene construct (from Oxitec, Oxford, UK). The latter transgene will fatally disrupt the development of insects carrying this gene (particularly

the progeny of mating between transgenic insects and wild type insects) when these insects are not supplied with a specific small molecule repressor (a tetracycline derivative). These cage studies are designed to test the function and effectiveness of autocidal transgenes and to determine the effectiveness of these autocidal insects in reducing experimental populations in a fully-contained experiment. Also, this experiment will compare the mating biology of these fluorescent-protein-producing, autocidal insects to that of wild type colony insects and irradiated wild type colony insects in a fully contained experimental environment. Implementation of genetically marked autocidal insects (with fluorescent proteins) into a PBW mass-rearing SIT program could provide a more effective alternative to irradiation or could reduce the necessary radiation dose to implement SIT, thereby increasing the effectiveness of an already demonstrably successful control program for the exotic pest, PBW. Finally, a genetically marked insect will be a useful monitoring tool for field managers to determine the distribution of treated PBW and to gauge the most efficient means of doing so.

Initial studies with males and females will be conducted in 3.6 x 7.3 x 1.8 m screen field cages placed over cotton plants at the CPHST rearing facility. The site is surrounded by an 8 foot chain link fence, topped with razor wire with locked gates, video surveillance and limited entry authorization. It is at least 3 miles from the nearest cultivated cotton. Adults, though capable of flight, will be contained in field cages. The structure of the field cages is 2.54 cm galvanized pipe covered with Lumite™ Saran™ 20 x 20 mesh fiberglass screen with reinforced corners to prevent tears. This mesh is tighter than mesh used in previously contained studies and as such is even less likely to allow escapes of contained moths than the materials used in USDA APHIS permits No. 03-104-01R and 01-029-01R. Though the adult moths cannot burrow, the cages also have a 30.5 cm plastic skirt running along the bottom that is buried in the soil to prevent moth escapes through soil cracks. An alternative site for confined studies is the same site used under permit 01-029-01R. This alternative site is surrounded by a 6 foot chain link fence.

Escape from such field cages is highly unlikely barring a major weather catastrophe, which itself is likely to destroy the contained insects. Eight pheromone traps baited with 2 mg of Gossyplure™ will be strategically distributed around the cage area to capture any males that might escape from the cages. All cotton plants in the area will be contained in the cages. Once the experimental work is done, all fruiting forms on the cotton will be removed and frozen at – 20°C for 24 hrs to eliminate all PBW life stages.

PBW control strategies will be in place and ready for deployment. They include pesticides and application equipment that have been used or are currently being used to contain and/or control PBW populations. The implementation of these strategies around the field cages and pheromone monitoring and control traps will make the risks negligible for the transgenic strain of PBW to transfer its genetic components to a field population of PBW or of this strain becoming established in the field. Risk is further minimized by research which has established that laboratory rearing of over 74 generations of the transgenic PBW strain give no indication that a transgenic EGFP strain has any competitive advantage over the strains currently maintained in the pink bollworm rearing facility.

Transgenic PBW that are no longer needed will be disposed of by freezing at -20° C for 48 hours. This will destroy any life stage of this insect. All plant fruiting forms in the release cages will be frozen at -20° C for 24 hours when the study is completed. This treatment will destroy PBW life stages that may infest the fruiting forms.

Previously conducted experiments demonstrated there are no transposases in the PBW genome that mobilize *piggyBac* transposon. This fact is addressed in the EA that has already been conducted resulting in the decision to issue APHIS permits No. 03-104-01R and 01-029-01R. Since there is no identifiable direct effect of this field test on any wild plant or animal species, there is no apparent risk to any threatened or endangered species. The proposed experiments are not expected to cause any adverse environmental effects due to their physical and biological containment. PWB also has no sexually compatible relatives in the United States with which it could reproduce or hybridize.

The application was submitted pursuant to regulations found in 7 CFR Part 340 which regulate the importation, interstate movement, or release into the environment of geneticat1y engineered plant pests. The regulations require that a person obtain a permit from APHIS prior to introducing a regulated article. This letter serves to give notice to and affords the State of Arizona the opportunity to indicate concurrence or non-concurrence with APHIS' assessment that contained field testing of these genetically modified insects does not pose a plant risk. You may also provide any conditions that may be mandated by your State. Please review the enclosed documents and return acknowledgement, associated comments, or reasons for non-concurrence (if applicable) to APHIS within 30 days from the date of this letter or preferably sooner (please use the enclosed form; use additional sheets for response, if needed).

Please refer to permit No. 05-118-01r in your correspondence regarding this application. If you have any questions about this application, please contact me at (301) 734-5720, facsimile (301) 734-8669, or e-mail: john.j.peloquin@aphis.usda.gov.

APHIS hopes to maintain its excellent working relationship with your State and encourages your participation and comments prior to our final decision regarding this permit application.

Sincerely,

John J. Peloquin, Ph.D.
Supervisory Biotechnologist/Entomoligst
Animals Branch Chief
Biotechnology Regulatory Services

Enclosures:
Permit Application No. 05-118-01r
State Response Form
