# Supplementary material for: Scientific Standards and the Regulation of Genetically Modified Insects
Source: PLoS Negl Trop Dis. 2012 Jan 31;6(1):e1502. doi: 10.1371/journal.pntd.0001502 (PMC3269433; doi:10.1371/journal.pntd.0001502)
Supplement: Supporting File S5 — US Environmental Assessment 2001-EA. (PDF) [file pntd.0001502.s005.pdf]

Confined Field Study of a Transgenic Pink Bollworm, Pectinophora gossypiella (Lepidoptera: Gelechiidae

Environmental Assessment

I. SUMMARY

This Environmental Assessment was prepared to asssess any potential environmental effects of a confined field study of a transgenic pink bollworm, Pectinophora gossypiella.

The application for a permit was submitted January 17, 2001 by the U.S. Department of Agriculture (USDA), Animal and Plant Health Insection Service (APHIS), Plant Protection Center, Phoenix, Arizona. The application is located at: http://www.aphis.usda.gov/biotech/arthropod/

The pink bollworm (PBW) is one of the most destructive pests of cotton in the world. It was first found in the United States in 1917 and it has become a pest in Texas, New Mexico, Arizona, and California. Costs relating to prevention, control, and yield losses have been estimated by the National Cotton Council to be more than $24 million annually. The San Joaquin Valley of California remains the last cotton growing area in the Southwest that is not generally infested with PBW. Prevention of its establishment in this valley is attributed primarily to the ongoing Sterile-Insect Technique (SIT) program established jointly in 1968 by the APHIS, California Department of Food and Agriculture, and the California cotton growers.

The objective of the proposed research associated with this permit application is to genetically engineer a strain of pink bollworm with an enhanced green fluorescent protein (GFP) marker gene derived from a jellyfish for field experimentation and performance studies. The use of a genetically marked insect in a PBW-SIT program would provide an additional tool for field managers to use in decisions involving efficient distribution of sterile PBW. The ability to identify the origin of native moth captures in the San Joaquin Valley of California is paramount to optimizing release strategies for this program.

The multiple levels of physical and biological confinement in the proposed field tests are: (1) isolation by distance; (2) isolation by screen cages; (3) reproductive sterilization; (4) removing wings of females and placing them in secondary cages; (5) male pheromone traps; (6) destruction of the cotton that may contain bollworms; (7) flooding the area with a high-ratio of sterilized bollworms; and (8) insecticide treatment, if required.

The stability of the transgene was demonstrated by rearing at the APHIS Phoenix Plant Protection quarantine rearing facility of 20 generations of enhanced GFP strain PBW with no evidence of instability of the enhanced GFP transgene. The only discernable

difference found in the biology of the enhanced GFP strain PBW, when compared to its non-genetically modified parental strain, was that the enhanced GFP female moths produced 19.8% fewer eggs than non-transformed PBW and their successful egg hatch rate was 26% lower.

II. REGULATORY AUTHORITY

The United States Department of Agriculture (USDA), Animal and Plant Health Inspection Service (APHIS) regulations under 7 CFR Part 340, Introduction of Organisms and Products Altered or Produced Through Genetic Engineering Which are Plant Pests or Which There is Reason to Believe are Plant Pests, were promulgated pursuant to authority granted by the Plant Protection Act (7 U.S.C. 7701-7772). These regulate the importation, interstate movement, or release into the environment of certain genetically engineered organisms and products. A genetically engineered organism is considered a regulated article if the donor organism, recipient organism, vector or vector agent used in engineering the organism belongs to one of the taxa listed in the regulation and is also a plant pest, or if there is reason to believe it is a plant pest. The pink bollworm, Pectinophora gossypiella (Saunders), is the recipient organism and a plant pest. The donor source of the transposon, piggyBac, is the cabbage looper, Trichoplusia ni (Huebner), which is also a plant pest.

The authority for 7 CFR Part 372, National Environmental Policy Act (NEPA) Implementing Procedures, is 42 U.S.C. 4321. Under 7 CFR § 372.5, Classification of actions, (c) Categorically excluded actions include (ii) Permitting, or acknowledgment of notifications for, confined field releases of genetically engineered organisms and products, except (4) when a confined field release of genetically modified organisms or products involves new species or organisms or novel modifications that raise new issues. A genetically modified pink bollworm field trial outside, even though it is highly confined, may meet this exception to NEPA categorically excluded actions, and, therefore, be subject to preparation of an Environmental Assessment.

III. DESCRIPTION OF REGULATED ARTICLE

A. The pink bollworm

The pink bollworm was described from larvae recovered from infested cotton bolls in India in 1843. It has since become one of the most destructive pests of cotton in many of the major cotton-growing regions of the world and may be the most destructive pest of cotton worldwide. The first reported cotton infestation in North America occurred in 1911 in Mexico, presumably from Egyptian cotton seed shipments (Nobel 1969). In the United States, the pink bollworm was detected first in Robertson County, Texas in 1917 (Scholl, 1919). By 1926, the pest had spread from Texas through New Mexico and into eastern Arizona, and it became a major economic pest of cotton in Arizona, southern

California, and northwestern Mexico by 1965 (Burrows et al., 1982). This insect is now a pest in Texas, New Mexico, Arizona and California. It has also occurred in Oklahoma, Arkansas, Mississippi, Missouri, Tennessee, Louisiana, and in southern Florida. It prefers cotton, but will feed on okra, kenaf, and hibiscus. The USDA, APHIS assists States in controlling the pest and preventing its spread to other States. APHIS enforces a quarantine in infested areas, requiring certification for the interstate movement of regulated articles. From: October 1995, USDA, APHIS, Plant Protection and Quarantine. URL at: http://www.aphis.usda.gov/oa/pubs/fspbw.htm

Pink bollworm larvae feed inside the growing cotton boll, destroying the cotton. Costs relating to prevention, control, and yield losses have been estimated by the National Cotton Council of America to be more than $24 million annually. In Egypt, China, and Brazil, it commonly causes cotton losses of up to 20 %, although losses can be much higher.

The PBW has four stages of development: egg, larva, pupa, and adult. In early June, female moths lay 100 to 200 eggs on young cotton bolls. The eggs hatch in about five days and develop into larvae, the stage that damages cotton. Pink bollworm larvae are 7 to 10 mm long (1/4 to 3/8 inch). First and second instar larval bodies are ivory in color with dark heads. Late instar larvae have bodies with pink bands. Adult moths are grayish brown and about the same length. Their wingspan is 15 to 20 mm (5/8 to 7/8 inch). Larvae bore into the cotton bolls and feed from 10 to 14 days on the seed. One larva eats a whole seed or parts of several seeds. When larvae finish feeding, they either drop to the ground or remain in the boll to pupate. Pupation can also take place under ground trash. Pupae emerge as moths in 8 to 10 days. The female moths mate and start laying eggs 1 to 3 days later. Adults are active only at night and live about 10 days. In warmer areas, most larvae overwinter in cotton or okra pods left in the field after harvest. In colder climates, larvae may form cocoons in the soil for overwintering. Larvae can also remain in cotton seed after the cotton is ginned, and if the seed is not fumigated, some of the larvae can emerge from the stored seed the next spring. The pink bollworm is well adapted to the long growing seasons prevalent in the desert valleys of the southwest where 5 to 6 generations develop each year. Egg-to-adult development takes 26-32 days during the cotton-growing season.

Pink bollworm Sterile-Insect Technique (SIT) program

The San Joaquin Valley of California remains the last cotton growing area in the Southwest that is not generally infested with PBW. Prevention of its establishment in this valley is attributed primarily to the ongoing Sterile-Insect Technique program established in 1968 jointly by APHIS, California Department of Food and Agriculture, and the California cotton growers (Miller et al.1984). All sterile PBW used in this project are mass-reared in a facility located in Phoenix, AZ. Sterile release occurs from May to mid-October, when pink bollworms are most active in cotton. For the sterile release method to succeed, a ratio of 60 sterile moths to one wild moth is needed. This high ratio of sterile

to fertile moths makes sterile releases impractical in heavily infested areas. One of the premises of a program employing SIT is that the mass-reared and sterilized insects can compete successfully for mates with their native counterparts. Van Steenwyk et al. (1979) reported that mass-reared irradiated PBW males were less competitive than their native counterparts and that mass-reared and irradiated PBW females were equal to or more competitive than native females. However, he also indicated that the combined release of both male and female PBW provided a sterile population that was as competitive as native males and females in mating ability. The current PBW-SIT program releases both sexes. Miller et al. (1994)b reported that native and sterile PBW females were comparable in attracting and successfully mating with native males when confined in field stations. The authors also indicated that sterile male PBW entered commercial pheromone traps during the same time interval as native PBW males. Male and female PBW both mate more than once, requiring the PBW-SIT program to maintain relatively high ratios of sterile-to-native insects. The dynamics of the sterile insect release strategy and its correlation to an insect's mating behavior is discussed by Davidson (1974). The strategy used in the PBW-SIT program is to release moths season long (average of 160 release-days per year) on approximately 25,000 hectares of the 350,000 hectares of cotton planted yearly in the San Joaquin Valley of California. The objective of the program is to prevent PBW moths blown into the San Joaquin Valley on storm systems originating in Mexico and the Southern California cotton growing regions from establishing infestations (Staten et al. 1992).

Besides the sterile release program, other PBW integrated pest management (IPM) measures are discussed in the Appendix.

B. Development of an engineered pink bollworm

1. Transformation system

The genes used from the donor organism and the piggyBac-derived portions of the vectors used to build the transforming construct were cloned off site. Specifically, Escherichia coli was the immediate host for the plasmids carrying the cloned genes used to make the transforming constructs. The piggyBac transposable element was discovered in cabbage looper cell culture at the University of Notre Dame (Fraser et al., 1995; Fraser et al., 1996; Wang and Fraser 1993). The Bombyx mori actin A3 promoter was cloned and modified by Thibault at the UCR, using polymerase chain reaction (PCR) from the embryos of Bombyx mori purchased from Carolina Biological Supply Company.

The transformed PBW strain produced at the University of California, Riverside (UCR) originated from the mass-reared "C" stock of the Pink Bollworm Rearing Facility, (PBWRF) in Phoenix, AZ. The origin of this PBWRF stock is from commercial cotton fields located in the Colorado River basin of California and Arizona. The PBW strains maintained in the PBWRF have been in existence since at least 1970. However, the colonies are periodically outcrossed with endemic field populations of PBW. The

parental strain that was transformed was last outcrossed with wild-type PBW in 1996. All final engineering of the transforming constructs were performed at UCR. Of the transgenic PBW strains produced by UCR scientists (Peloquin, Thibault et al. 2000), one strain (#35) was transferred to the APHIS, Plant Protection Laboratory in Phoenix, Arizona under USDA/APHIS permit No. 98-244-02m for movement of transformed insects between laboratories in Riverside and Phoenix.

2. Green fluorescent protein and piggyBac

Green fluorescent protein and the ability of its derivatives to function as dominant, visible, nondestructive markers of insects (Brand 1995), mammalian (Pines 1995), and plant systems (Haseloff et al., 1997) were indicators of its potential use in PBW.

The Enhanced Green Fluorescent Protein (EGFP) gene is an enhanced version of the green fluorescent protein (GFP) gene cloned by Prasher, USDA/APHIS Otis AFB, MA, from the jellyfish, Aequora victoria (Cubitt et al., 1995; Heim et al., 1994; Heim and Tsien, 1996; Prasher, 1995). The plasmid source of GFP was purchased from Clontech, Inc. Previous plasmid-based mobility assays demonstrated that piggyBac or elements producing piggyBac-like transposase are not present, but when piggyBac is introduced as a donor/helper system, it is mobile in PBW embryos (Thibault et al., 1999). Therefore, a piggyBac vector was constructed containing enhanced green fluorescent protein as a marker for transformation.

The piggyBac element is a deoxyribonucleic acid (DNA) transposable element capable of integrating into other DNA through mediation of a transposase encoded by a transposase Open Reading Frame (ORF) within the element, only when its Inverted Terminal Repeats (ITR) are intact. In the construct used for transformation of the PBW, the transposase gene of the piggyBac element was destroyed by insertion of an expression cassette containing EGFP ORF driven by a single copy of the Bombyx mori-derived BmA3 promoter. This manipulation destroys the ability of the transformation construct to move on its own. Transformation was done by co-injecting a transposition and integration incompetent helper plasmid along with a donor plasmid into early stage PBW embryos. The donor plasmid contains the transforming construct flanked by piggyBac ITRs. The helper plasmid encodes an intact piggyBac transposase ORF. The gene product of this piggyBac transposase ORF is under the control of a promoter, that directs insect cells to express piggyBac transposase after injection. Importantly, the helper plasmid lacks the downstream piggyBac ITR. These ITRs are absolutely essential for piggyBac transposase mediated integration. Therefore, the helper plasmid lacking one or the other of the ITS cannot integrate itself into target DNA in a transposase-mediated event.

The potential for instability and unwanted mobilization of piggyBac-derived transforming constructs is addressed in the following: Although there is no evidence for

any piggyBac transposase activity in the PBW genom, it could be argued that, if there were endogenous piggyBac-like elements undetected in the applicants screen for these elements in PBW, they might provide a source of transposases that could mobilize EGFP transgenes flanked by piggyBac-derived ITRs. Demonstration of elements homologous to piggyBac in the recipient PBW might then imply some instability of the EGFP transgene. However, there is no evidence for piggyBac-like transposases in PBW (Thibault et al., 1999). Additionally, the DNA-mediated element, Hermes, has been used to successfully transform Aedes aegypti with little or no evidence of instability of the transgenes over at least 10 generations, even though there are endogenous elements (hAt-like, as is Hermes) in Aedes aegypti with close enough homology to Hermes, so that these endogenous hAt and Hermes-like elements are detected in higher stringency Southern blots with a Hermes probe (Jasinskiene et al 1998). In the case of pink bollworm, low stringency Southern blot experiments on pink bollworm DNA with radiolabeled DNA probes derived from piggyBac, which would be even more likely to detect elements with low homology to piggyBac than the higher stringency methods used in Jasinskiene, et al., 1998, were unable to detect any endogenous piggyBac-like elements. This indicates there are no elements in the PBW that might reasonably be expected to mobilize a piggyBac-derived transgene. In addition, excision and transposition assays were performed in PBW embryos with piggyBac. This was primarily to determine if piggyBac could integrate into the PBW genome. However, results of transposition assays did not show transposition of piggyBac in the absence of exogenous piggyBac transposes, indicating there were no unknown piggyBac-like elements in the PBW genome (Thibault et. al. 1999). Thus, there should not be unexpected interactions between the components of the PBW genome and the transforming construct that could result in instability of the transgenes.

3. Characterization of engineered pink bollworms

a. Molecular

Insertion of the piggyBac element into genomic DNA was detected by Southern blot analysis using one of the positive lines. The presence of at least two insertions was detected in this line with the probe recognizing approximate 1.9-kb and a 2.3-kb bands. Individuals examined contained either one of the inserts, or both. Based on inverse PCR, the piggyBac integration appears to have been a singular event which occurred in a transposase-dependent manner resulting in a target site duplication with no plasmid sequences flanking the transposon ends. Immunoblot analysis using a green fluorescent protein-specific antibody was also used to differentiate expression of EGFP from autofluorescence in wild-type animals and establish that the EGFP protein produced was the expected size showing that no additional sequence was being translated into protein fused to the EGFP.

The helper plasmid contained a piggyBac transposase gene driven by the Drosophilia hsp70 heat-shock promoter instead of the endogenous piggyBac promoter that was obliterated by introduction of the Drosophilia hsp70 promoter. Integration of the Bombyx

mori A3 EGFP construct, which lacks sequences found in the original element, also demonstrates that the whole piggyBac element is not essential for transposase-mediated transposition. The complete element is 2.5 kb in length. Construction of the vector resulted in deletion of approximately 1 kb within the original piggyBac transposase open reading frame, resulting in inactivation.

b. Genetics; Stability of integration

The green fluorescent protein positive lines were maintained as heterozygotes at the insertion locus by serial backcrosses to the wild-type strain. At the time of backcross analysis, the lines had been backcrossed for four generations. This would likely separate any transformed loci that were not tightly linked. Thus, the EGFP-positive parental insects used in the diagnostic backcrosses were expected to be heterozygous for a single copy of the transgene. At the time of backcross analysis of the heterozygote lines, the first line produced 191 positive and 207 negative progeny and the second line produced 555 positive and 616 negative progeny. These were not significantly different from the expected 1:1 ratio by 2 statistical analysis. Therefore, a relatively close 1:1 ratio of EGFP versus wild-type supports the hypothesis that the EGFP was transmitted as a single-locus, dominant gene.

c. Fitness compared to wild-type

The stability of the transgene was demonstrated further by the rearing at the Phoenix Quarantine Facility of 20 generations of EGFP strain PBW with no evidence of change of the EGFP transgene. This study found no differences in length of time spent in larval instars, and the pupal stage in EGFP PBW compared to non-transformed PBW. However, the EGFP female moths produced 19.8 % fewer eggs than non-transformed PBW and their successfrul egg hatch rate was 26% lower (Miller et al., 2001).

IV. DESCRIPTION OF RESEARCH PROPOSED BY APPLICANTS

The experiments would be conducted on nonresidential land used used to grow cotton. Arrangements will be made to protect the experimental area against accidental pesticide treatment and vandalism that could disrupt the intended experiments.

Before genetically transformed insects can be considered for use in SIT programs, more must be known about their behavior and performance under field conditions. Such testing will require field releases, which would employ confinement measures. The application for a permit to release the EGFP strain of PBW has been submitted by the APHIS, Phoenix Plant Protection Laboratory. The releases will be made in field cages located in a cotton field in Maricopa County, Arizona.

Since the existing PBW SIT program strategy is effective only at low population densities, the goal of the research is to improve SIT. One of the first improvements in SIT offered by genetic transformation is to provide a reliable marker that can be detected in the field. The native moths captured in the San Joaquin Valley of California as part of the SIT program are distinguished now from the mass-reared adults by the presence of a dye incorporated in the diet in the rearing facility.

Miller et al. (1994)a indicated that native catches in the San Joaquin originated from possibly four sources. They are listed here in descending order of likelihood: (1) Migrant populations from the heavily infested, more southern desert valleys of California; (2) Small native populations that do not reach detectable levels until late in the cotton growing season; (3) F1 progeny developing from an out-cross of a native or migrant population and mass-reared release moths; and (4) F1 progeny from a self-cross of irradiated release moths.

The presence of the EGFP genetic marker in PBW would provide a means to identify F1 progeny from mating between the released insects and natives. These "native" catches (F1 progeny of released insects) would not require increased releases of sterile moths because of the high degree of inheritied sterility of such crosses (Miller et al. 1984). Current program policy requires that fields with native finds receive higher release rates than those without native finds. Genetically marked insects can be distinguished from a native pink bollworm by screening with a fluorescent microscope and/or PCR (Peloquin, J. J. and T. A. Miller, 2000).

Transgenic insects reared in the Phoenix Plant Protection Quarantine Laboratory and selected for field release will be released in screen cages (3.6 by 7.3 by 1.8 m) placed over cotton in a field in Maricopa County, AZ. The structure of the field cages consist of a 2.54-cm galvanized pipe frame covered with a 16 x 16 mesh (256 openings per square inch) fiberglass screen with reinforced corners to prevent tears. The cage also has a plastic skirt 30.5 cm in width along the bottom, which is buried in the soil to prevent moth escapes. The selected cotton field is about three acres in size and located in a nonresidential urban area. No other cotton fields are within three miles of this field. Although there are a few ornamental hibiscus around residences within a mile of the testing site, the permit applicants have found PBW will not complete its biological development on the contemporary ornamental cultivars available from nurseries and other retailers.

All released insects will be irradiated with 20 kilorad (kr) of Cobalt60 before release to insure sterility. This treatment produces virtually 100% sterility. A previous study by Miller et al. (1984) indicated under laboratory conditions, that a population of 720,000 PBW produced one normal adult per 1000 parent females. Under field conditions, no F1 progeny were produced from the release of 2.25 million PBW in field cages. Only male moths of the genetically modified strain of PBW have the potential to be dispersed into

the environment. If this were to happen, the males are sterile. Therefore, any mating that might occur between an escaped male and a wild-type female would result in no offspring.

All released females will have their wings clipped so they cannot fly. They will also be contained inside the primary screen cages in paper 1-gallon bucket secondary cages with an open top and a barrier of axle grease to prevent the non-flying females from crawling out of the containers.

Pheromone traps will be placed in a grid pattern in the field surrounding the cages to capture any males that might escape the cage. If a catastrophic event should occur to the field cages, all fruiting forms under the cages will be collected and destroyed and the field will be treated with a high-ratio of sterilized PBW. Insecticides may also be used as a final measure of control, if required.

Transgenic PBW that are no longer needed will be disposed of by freezing at -20° C for 24 hours. This will destroy all life stages of this insect. Transgenic PBW recaptured in the field trails will be disposed of by freezing at -20° C for 24 hours. All plant fruiting forms in the release cages will be disposed of by freezing at -20° C for 24 hours when the study is completed. This will destroy life stages that may infest the fruiting forms.

The purpose of the cage releases will be to determine field fitness of the EGFP strain of PBW and compare its performance to its mass-reared nontransgenic counterparts.

Fitness tests to be performed in the field cages will include the following tests:

TEST 1--This test will compare male response to pheromone traps of EGFP strain and non-EGFP strain PBW. This test will be replicated six times using two cages with 2-week intervals between releases using 50 males per replicate per treatment. All EGFP strain males will be marked externally with a pink florescent dye. Control (non-EGFP) insects will be marked with a blue florescent dye. Two Delta™ traps baited with 2 mg of gossyplure PBW pheromone impregnated in a rubber septum will be placed in each cage 24 h after the moths are released. Traps will be examined daily for moth captures. All moths will be sterilized with a radiation dose of 20-kr of Cobalt60 for an additional measure of biological confinement.

TEST 2--This test compares male longevity in the field of EGFP and non-EGFP strain PBW. Each of three field cages will receive 100 EGFP strain PBW males and 100 non-EGFP strain males. Each PBW strain will be marked with a different color fluorescent dye as described in Test 1. Following moth release, one of the three cages will be

randomly assigned to have two Delta traps baited with 2 mg of gossyplure placed in the cage five days after the moths are released. Of the remaining two cages, one will have two traps placed in the cage on day-10-post release and the remaining cage will have the two traps placed in the cage on day-15-post release. The test will be replicated five times using 300 males per treatment, per replicate. All moths will be sterilized with a radiation dose of 20-kr of Cobalt60 for an additional measure of biological confinement.

TEST 3--This test compares the EGFP and non-EGFP females' ability to solicit and mate with their male counterparts and males from the different strain. Each female moth will have the wings on the left side surgically removed while under cold anesthesia in the laboratory. Pairs of females, consisting of one EGFP and one non-EGFP strain, will then be placed for 24 h in a 9-dram vial fitted with 0.5-dram feeder vial containing a 6% sucrose solution. These vials will be used to transport the moths to the field mating stations located in the screen field cages. Pairs of female moths will be placed in each of ten mating stations at dusk along with a cotton leaf and exposed to 100 released males. At dawn on the following morning, the females will be removed from the stations and returned to the laboratory. Each female will then be dissected to determine mating by the presence of a spermatophore in the bursa copulatrix. If mating has occurred, the type of male she mated with will be determined. Males from the non-EGFP strain will be mass-reared on a larval diet that contains a fat-soluble dye that internally marks the insect. The dye is also retained in the spermatophore that the male transfers to the female. Thus, the spermatophore from non-EGFP strain males can be differentiated from those of the EGFP strain when examined under a microscope. All moths will be sterilized with a radiation dose of 20-kr of Cobalt60 for an additional measure of biological confinement.

V. ALTERNATIVES

A. Deny the Permit Application. This alternative would provide no improved means of monitoring the effectiveness of the SIT program. Two other ways to mark pink bollworms are described as follows: Miller et al. (1994)a reported on a genetically marked mass-reared strain of PBW. Although the PBW-SIT program has implemented the use of genetic markers through mutants produced by classical genetic technique, homozygosity was not maintained in these genetically marked strains of PBW when exposed to the highly selective processes of mass- rearing. Bollworms can also be reared on a larval diet that contains a fat-soluble dye that internally marks the insect, but this method of marking is not inherited and is limited to the immediate generation. Therefore, both methods are of much less value compared to EGFP genetic marking.

B. Issuance of the Permit. This use of EGFP marked strain of the pink bollworms is for the purpose of making the SIT program more effective. In addition, biological fitness data will be obtained on the genetically modified strain of PBW.

VI. POTENTIAL IMPACTS

A. Deny the Permit Application. The denial alternative consists of the unimproved SIT program described above, or if that program is discontinued or does not succeed, then the remaining alternatives are the use of multiple organophosphate, carbamate, and synthetic pyrethroid insecticides, cultural control, transgenic Bacillus thuringinesis toxin expressing cotton, and other IPM measures such as described in the Appendix. Both the classical genetic marker system and the fat-soluble dye marker incorporated in the reared insects' diet are insufficient to monitor PBW populations for the SIT program.

B. Issuance of the Permit. The proposed action is not expected to have any adverse environmental impacts for the following biological and physical reasons:

No ecological or other consequences are expected from incorporation of this marker into the transgenic pink bollworm. The unmodified pink bollworm has no EGFP gene; therefore, it does not fluoresce characteristically green when illuminated. Neither piggyBac transposase replication activity, nor any antibiotic resistance is conferred to the transgenic PBW by the introduced genetic material as genetic material encoding these proteins was not integrated into the PBW genome.

Possibility that the genetically modified organism will undergo some form of unanticipated genetic transformation that may effect the environment

The possibility of the genetically modified organism reverting to or undergoing some form of unanticipated genetic transformation are exceedingly low. The proposed field tests are designed to prevent reproduction and to prevent any unexpected traits from being transmitted to offspring.

Persistence compared to wild-type

It is highly unlikely that the EGFP gene would persist in the environment because it provides no fitness advantage to the PBW. A 20-generation study of EGFP PBW showed that there was a loss of fitness evidenced in the female's ability to produce eggs and egg survivability was also reduced in the EGFP strain. The enhanced EGFP female moths produced 19.8% fewer eggs than non-transformed PBW and their successful egg hatch rate was 26% lower (Miller et al., 2001). Even if the EGFP gene were present in a field population of the pink bollworm, it would not confer a selective advantage and the insects would likely lose the gene and revert to wild-type insects over time.

Physical and biological confinement

The multiple levels of physical and biological confinement in the proposed research are: (1) isolation by distance; (2) isolation by screen cages; (3) reproductive sterilization; (4) removing wings of females and placing them in secondary cages; (5) male pheromone traps; (6) destruction of the cotton that may contain bollworms; (7) treating the area with a high ratio of sterilized bollworms; and (8) insecticide treatment, if required.

Gene transfer to related species

The PBW is not native to the United States and there are no known sexually compatible species in North America.

Gene transfer to predators, saprophytes, or parasites

Pink bollworms may be eaten by other predatory insects, birds, or mammals that venture into cotton fields. However, only the adult stage will be tested which further reduces any chance of predation or parasitism since larvae are typically preferred more than adults. The green fluorescent protein is a naturally occurring protein, not known to cause adverse effects. The gene has been found in nature only in the jellyfish from which it is derived. Jellyfish have been prey or subject to saprophytic digestion by other organisms since their origin. There is no current evidence that this gene has been transferred through predation, natural decay, or parasitism. The normal digestive process of predators would preclude transfer of functional genetic material to the predator and this phenomenon is not expected with insectivores. Pink bollworms may also serve as hosts for parasitic insects, nematodes, and various microorganisms. These parasitic and infectious organisms are unrelated to the PBW and would not be expected to assimilate functional DNA from their hosts leading to modification of the parasite or microorganism.

Potential impacts on humans, including minorities, low income populations, and children

These requirements are specified in Executive Orders 13045 and 12898 and address the identification of health or safety risks that might disproportionately affect children or have adverse impacts on minorities and low income populations. The proposed actions are not expected to adversely affect any of these groups and may benefit them by contributing to the reduction of pesticide exposure from habitation near cotton fields and occupational pesticide exposure of cotton workers. Lepidoptera were also specifically excluded from consideration as human health hazards by a working group of the American Society of Tropical Medicine and Hygiene on biosafety of transgenic arthropods.

Effects on chemical (pesticide, herbicide, fungicide) load on the environment

US Environmental Protection Agency registered pesticides, primarily insecticides, are used more intensively on cotton than most other crops. The use of the EGFP marker gene to facilitate the pink bollworm SIT program contributes to lowering the pesticide load on the environment.

Risks to nontarget plants and animals including threatened and endangered species

The testing is both biologically and physically highly confined and there are no threatened or endangered species in proximity to the test cages and the surrounding three-acre cotton field in Maricopa County, Arizona. There is no apparent risk to any threatened or endangered species because there is no identifiable direct effect of this field test on any wild plant or animal species. Cotton is usually subject to intensive pesticide treatment due to its many pests. Endangered or threatened species would be at much higher risks due to pesticides used on cotton than by the nature of these experiments that evaluate EGFP marking of pink bollworms to facilitate the SIT program. The proposed alternative would help decrease the need for pesticide use and thereby lower the likelihood of spray drift or runoff to environmentally sensitive habitats. The corresponding decrease in PBW associated with the SIT program results in less need for pesticide applications and less risk to nontarget species in general. The decreased pesticide use under the proposed alternative would also be expected to contribute to less pesticide contamination of soil, water, and air.

Likelihood of controversy of effects on environmental quality

The effects on environmental quality are expected to be nonexistant or negligible because the EGFP marker gene is not known or expected to negatively impact the environment. Additionally, the experiment is biologically and physically highly confined. However, some controversy is expected because the experiments involve a genetically modified insect and many people inordinately fear or dislike insects and other arthropods such as spiders. This experiment was specifically suggested by a working group sponsored by the Food and Agriculture Organization of the United Nations as a first release experiment using transgenic arthropods (Ashburner et al. 1998).

Degree of possible uncertain effects on the environment and unique or unknown risks

The experiments are biologically and physically highly confined and the EGFP gene is not known or expected to affect the environment, therefore, the degree of uncertainty is negligible. Furthermore, Miller et al. (2001) have shown that the EGFP gene provides no selective advantage over 20 generations of the PBW. Unique or unknown risks in the proposed project are expected to be negligible.

Consistency of proposal with other environmental requirements

The proposal is believed to be consistent with other environmental requirements and is intended to be part of an IPM program that reduces the use, costs and risks from broad spectrum toxic insecticides that are capable, when misused, of causing severe adverse effects on nontarget organisms and human health.

VII. CONCLUSION

Potential environmental risks of the proposed action and its alternatives are discussed with emphasis on the following levels of biological and physical confinement: (1) isolation by distance; (2) isolation by screen cages; (3) reproductive sterilization; (4) removing wings of females and placing them in secondary cages; (5) male pheromone traps; (6) destruction of the cotton that may contain bollworms; (7) treating the area with a high-ratio of sterilized bollworms; and (8) insecticide treatment, if required.

The stability of the construct has been addressed in a 20 generation pink bollworm study that determined no competitive advantage and in a four generation backcross study that verified the EFGP gene was inherited in a 1:1 ratio as a single-locus, dominant gene. Even if the EGFP gene were present in a field population of the pink bollworm, it would not confer a selective advantage and the insects would likely lose the gene and revert to wild-type insects over time.

Experiments that were conducted prior to the proposed confined field tests demonstrated that there are no elements in the PBW genome that might mobilize a piggyBac-derived transgene.

Since there is no identifiable direct effect of this field test on any wild plant or animal species, there is no apparent risk to any threatened or endangered species. The proposed experiments described in this Environmental Assessment are not expected to cause any adverse environmental effects.

VII. LITERATURE CITED

Ashburner, M., M.A. Hoy, and J.J. Peloquin. 1998. Prospects for the genetic transformation of arthropods. Insect Mol Biol 7: 201-213.

Brand, A. 1995. GFP in Drosophila. Trends in Genetics 11 (8): 34-5.

Burrows, T.M., V. Sevacherian, H. Browning, and J. Baritelle. 1982. History and cost of the pink bollworm (Lepidoptera: Gelechiidae) in the Imperial Valley. Bull. Entomol Soc Am 28: 286-290.

Cubitt, A.B., R. Heim, S.R. Adams, A.E. Boyd, L.A. Gross, and R.Y. Tsien. 1995. Understanding, improving and using green fluorescent proteins. Trends Biochem Sci 20: 448-455.

Davidson,G. 1974. The genetic control of insect pests. Academic Press Inc. New York. pages 17-30.

Flint, H.M., D.L. Palmer, L.A. Bariola, and B Horn. 1974. Suppression of populations of native pink bollworm in field cages by release of irradiated moths. J Econ Entomol 67: 55-57.

Fraser, M.J., L. Cary, K. Boonvisudhi, and H.G. Wang. 1995. Assay for movement of lepidopteran transposon IFP2 in insect cells using a baculovirus genome as a target DNA. Virology 211: 397-407.

Fraser, M.J., T. Ciszczon, T. Elick, and C. Bauser. 1996. Precise excision of TTAA-specific lepidopteran transposons piggyBac (IFP2) and tagalong (TFP3) from the baculovirus genome in cell lines from two species of Lepidoptera. Insect Mol Biol 5: 141-151.

Haseloff, J., K.R. Siemering, D.C. Prasher, and S. Hodge. 1997. Removal of a cryptic intron and subcellular localization of green fluorescent protein are required to mark transgenic Arabidopsis plants brightly. Proc Nat Acad Sci 94 (6): 2122-7.

Heim, R., D.C. Prasher, and R.Y. Tsien. 1994. Wavelength mutations and posttranslational autoxidation of green fluorescent protein. Proc Nat Acad Sci 91: 12501-4.

Heim, R., and R.Y. Tsien. 1996. Engineering green fluorescent protein for improved brightness, longer wavelengths, and fluorescence resonance energy transfer. Curr Biol 6: 178-182.

Janinskiene N., C.J. Coates, M..Q. Benedict, A.J. Conrel, C.S. Rafferty, A.A. James, and F.H. Collins F.H. 1998. Stable transformation of the yellow fever mosquito, Aedes aegypti, with the Hermes element from the housefly. Proc Nat Acad Sci 95(7): 3743-7.

Miller, E., R.T. Staten, E. Jones, and J. Pozzi. 1984. Effects of 20 krad of gamma irradiation on reproduction of pink bollworm (Lepidoptera:Gelechiidae) and their F1 progeny: potential impact on the identification of trap catches. J Econ Entomol (77): 304-307.

Miller, E., D. Keaveny, R.T. Staten, A. Lowe, and J. Boomberg. 1994a. Changes in PBW (Lepidoptera: Gelechiidae) sooty mutant under APHIS mass-rearing methodology. J Econ Entomol 87 (6): 1659-64.

Miller, T., E. Miller, R Staten and K. Middleham.1994b. Mating response behavior of sterile pink bollworms (Lepidoptera:Gelechiidae) compared with natives. J Econ Entomol 87 (3): 680-6.

Miller, E., R.T. Staten, J. Claus, M. Sledge, J. Peloquin, T. Miller. 2001. A multiple generation life history study on rearing a genetically altered (EGFP) strain of pink bollworm (Lepidoptera:Gelechiidae) Proc Belt Wide Cotton Conf, January 9-13, Anaheim, CA

Noble, L.W., 1969. Fifty years of research on the pink bollworm in the United States USDA-ARS, Agric Handbook No. 357, March 1969.

Peloquin, J. J. and T. A. Miller. 2000. Enhanced green fluorescent protein DNA detection in pink Bollworm (Pectinophora gossypiella) through polymerase chain reaction amplification. J Cotton Sci 4:19-27.

Peloquin, J.J., S.T. Thibault, R. Staten, and T.A. Miller. 2000. Germ-line transformation of pink bollworm (Lepidoptera: Gelechiidae) mediated by the piggyBac transposable element. Insect Mol Biol 9(3): 323-333.

Pines, J., 1995. GFP in mammalian cells. Trends in Genetics 11 (8): 326-7.

Prasher, D.C. and V.K. Eckenrode. 1992. Primary structure of the Aequorea victoria green-florescent protein. Gene 11 (2):229-33.

Prasher, D.C. 1995. Using GFP to see the light. Trends Genet 11: 320-323.

Scholl, E.E. 1919. Report of the pink bollworm of cotton. Tex Dept Agr Bul 65. 459 pp.

Staten, R.T., R.W. Rosander, and D.F. Keaveny. 1992. Genetic control of cotton insects; The PBW as a working programme. Proc Internat Symp On Management of Insect Pests. Vienna Oct 19-23. 269-283. Pub International Atomic Energy Agency, Vienna,1993.

Thibault, S.T., H.T. Luu, N. Vann, and T.A. Miller. 1999. Precise excision and transposition of piggyBac in pink bollworm embryos. Insect Mol Biol 8(1): 119-23.

Van Steenwyk, R.A., Henneberry, T.J., Ballmer, G.R., W.W. Wolf, and V. Sevacherian. 1979. Mating competitiveness of laboratory-cultured and sterilized PBW for use in a sterile moth release program. J Econ Entomol (72): 50.

Wang, H.G., and J.J. Fraser. 1993. TTAA serves as the target site for TFP3 lepidopteran transposon insertions in both nuclear polyhedrosis virus and Trichoplusia ni geneomes. Insect Mol Biol (1): 109-16.

X. PREPARERS

Robert I. Rose, Ph.D.

Arthropod Biotechnologist

USDA, APHIS, PPQ, Unit 147

Riverdale, MD 20737

Phone: (301) 734-8723

Fax: (301) 734-8669

e-mail: bob.i.rose@aphis.usda.gov

Thomas Miller, Ph.D.

Professor of Entomology

Department of Entomology

University of California

Riverside, CA 92521

(909) 787-3886

e-mail: thomas.miller@ucr.edu

John J. Peloquin, Ph.D.

Assistant ResearchEntomologist IV

Department of Entomology

Riverside, CA 92521

(909) 787-4680

e-mail: peloquin@cache.ucr.edu

Robert T. Staten, Ph.D.

Center Director

APHIS Phoenix Plant Protection Center

3645 E. Wier Avenue

Phoenix, AZ 85040-2931

(602) 437-1295 Ext. 222

e-mail: r.t.staten@usda.gov

Ernest Miller

Entomologist

APHIS Phoenix Plant Protection Center

3645 E. Wier Avenue

Phoenix, AZ 85040-2931

(602) 379-4828 Ext. 233

e-mail: ernie.d.miller@usda.gov

IX. AGENCY CONTACT

Robert I. Rose, Ph.D.

Arthropod Biotechnologist

USDA APHIS PPQ Unit 147

Riverdale, MD 20737

Phone: (301) 734-8723

Fax: (301) 734-8669

e-mail: bob.i.rose@aphis.usda.gov

APPENDIX

Pink Bollworm Integrated Pest Management

Cultural control

The University of California Statewide Integrated Pest Management Project, Updated 12/98, at URL: http://www.ipm.ucdavis.edu/PMG/r114301511.html includes the following PBW recommendations: Eliminate the food supply for PBW by cutting off irrigation early enough to stop production of green bolls by early September. Regardless of when the crop is terminated, immediately shred the cotton plants following harvest. Shredding destroys some larvae directly and promotes rapid drying of unharvested bolls. If fall temperatures are high during September and October, leave crop debris on the soil surface for two or more weeks after the shredding operation to further destroy larvae. Be sure to comply with plow-down requirements and cross- disc or plow to a depth of at least 6 inches (15 cm). Winter irrigations can reduce populations of overwintering PBW by as much as 50 to 70% and flooding in December is more effective than flooding in November or January. Take advantage of PBW mortality afforded by winter irrigations and rotate to small grains or newly seeded alfalfa. In spring, irrigations can also be used to promote early emergence of PBW. If cotton is being followed with cotton, pre-irrigate in February and plant as early as possible, following guidelines to ensure adequate soil temperature for germination and emergence. Plan irrigations of the crop to prevent even slight moisture stress and to promote maximum emergence of moths in advance of susceptible squares.

Pheromones , Bacillus thuringinesis Modified Cotton, Nematodes, Parasites

The use of gossyplure, a sex pheromone attractant that disrupts mating when distributed throughout the field, may be effective against PBW when it is supplemented with cultural control practices (Staten et al. 1987). Genetically modified cotton that expresses Bacillus thuringiensis toxin is also effective to suppress PBW infestation and damage since the PBW is one of the more susceptible cotton pests to the Bt proteinaceous toxin produced in the cotton plant (Flint et al. 1995). Three insect parasitic nematodes are of some interest for biological control of PBW (Henneberry et al. 1995). They are Steinernema riobravis, S. carpocapsae and Heterorhabditis bacteriophora. Steinernema carpocapsae has been available commercially, but costs are high and there have been product storage and transit viability problems in the past. Several species of exotic insect parasites have been introduced in California and Arizona for biological control of PBW, however, none have become established or have had any significant effect on pest suppression.

Table: Pesticides recommended for use in California Pink Bollworm IPM
Pesticide Name Selectivity for PBW Persistence on Natural Enemies
Gossyplure High None

Chlorpyrifos (OP) Moderate Short
Cypermethrin (SP) Low Moderate
Esfenvalerate (SP) Low Moderate

OP = organophosphate, SP = synthetic pyrethroid

Other insecticides that have been used against the pink bollworm include methamidophos (OP), encapsulated methyl parathion (OP), azinphos methyl (OP), methomyl (carbamate), permethrin (SP), and lambda-cyhalothrin (SP). Most of these pesticides are broad spectrum in toxic activity and can affect nontarget organisms if exposed in sufficient quantities.

Monitoring

Sampling bolls is the most reliable way to monitor high PBW populations. The University of California Statewide Integrated Pest Management Project, Second Edition of Integrated Pest Management for Cotton in the Western Region of the United States, 1996, Publication 3305 contains recommendations. See URL: http://169.237.210.130/IPMPROJECT/ADS/manual_cotton.html for availability. This publication provides detailed sampling methods and thresholds for control. Another University of California Statewide Integrated Pest Management Project, Phenology Model Database, provides a method of predicting PBW development that may be useful for timing insecticide applications and cultural control measures. See URL: http://www.ipm.ucdavis.edu/PHENOLOFY/pinkbollworm.html for availability.

References

Flint, H.M., T.J. Henneberry and F.D. Wilson. 1995. The effects of transgenic cotton, Gossypium hirsutum L., containing Bacillus thuringiensis toxin genes for the control of the pink bollworm (Saunders) and other arthropods. Southwestern Entomol 20 (3): 281-292.

Henneberry, T.J., J.E. Lindgren, L. Forlow Jech, and R.A. Burke. 1995. Pink bollworm (Lepidoptera: Gelechiidae): Effects of Steinernematid nematodes on larval mortality. Southwestern Entomol 20 (1): 25-32

Staten, R.T., H.M. Flint, R.C. Weddle, E. Quintero, R.E. Zarate, C.M. Finnell, M. Hernandes, and Y. Yamamoto. 1987. Pink bollworm (Lepidoptera: Gelechiidae): large-scale field trials with a high-rate gossyplure formulation. J Econ Entomol 80 (6): 1267-71
